# Size-Dependent Target Engagement of Covalent Probes

**DOI:** 10.1021/acs.jmedchem.5c00017

**Published:** 2025-03-18

**Authors:** László Petri, Ronen Gabizon, György G. Ferenczy, Nikolett Péczka, Attila Egyed, Péter Ábrányi-Balogh, Tamás Takács, György M. Keserű

**Affiliations:** † Medicinal Chemistry Research Group and National Drug Discovery and Development Laboratory, HUN-REN Research Centre for Natural Sciences, 2 Magyar tudósok krt, Budapest 1117, Hungary; ‡ Department of Chemical and Structural Biology, Weizmann Institute of Science, Helen and Milton A. Kimmelman bldg, Rehovot 76100, Israel; § Department of Organic Chemistry and Technology, Budapest University of Technology and Economics, 8 Budafoki út, Budapest 1111, Hungary; ∥ HUN-REN Research Centre for Natural Sciences, Signal Transduction and Functional Genomics Research Group, 2 Magyar tudósok krt, Budapest 1117, Hungary; ⊥ Doctoral School of Biology, Institute of Biology, ELTE Eötvös Loránd University, Pázmány Péter sétány 1/A, Budapest 1117, Hungary

## Abstract

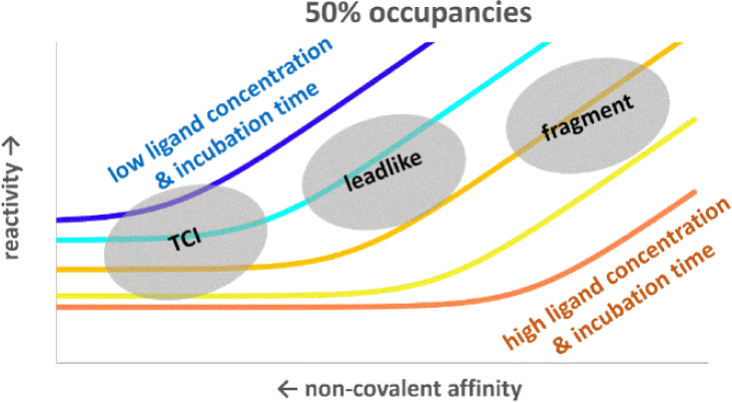

Labeling proteins with covalent ligands is finding increasing use in proteomics applications, including identifying nucleophilic residues amenable for labeling and in the development of targeted covalent inhibitors (TCIs). Labeling efficiency is measured by the covalent occupancy of the target or by biochemical activity. Here, we investigate how these observed quantities relate to the intrinsic parameters of complex formation, namely, noncovalent affinity and covalent reactivity, and to experimental conditions, including incubation time and ligand concentration. It is shown that target engagement is beneficially driven by noncovalent recognition for lead-like compounds, which are appropriate starting points for targeted covalent inhibitors owing to their easily detectable occupancy and fixed binding mode, facilitating optimization. In contrast, labeling by fragment-sized compounds is inevitably reactivity-driven as their small size limits noncovalent affinity. They are well-suited for exploring ligandable nucleophilic residues, while small fragments are less appropriate starting points for TCI development.

## Introduction

Recent applications of covalent fragments involve both target and ligand discovery. Chemoproteomic discovery of targetable proteins for therapeutic applications uses fragment-sized compounds that bind covalently to their protein targets. Fragments with modest reactivity can overcome the limitations of reversible noncovalent ligands and are able to efficiently recognize tractable proteins of the proteome. They can be used in native biological systems, and the resulting ligand-protein interactions can be analyzed by chemoproteomics methods.^1^ The screening of fragments against specific proteins can be used to identify chemical starting points for covalent ligand discovery.^2,3^ The most common targets of covalent labeling are cysteine residues, owing to their enhanced nucleophilicity and low abundance, which alleviates promiscuity issues when cysteine-specific warheads are applied. Targeting residues beyond cysteine extends the potential of protein labeling, as many relevant drug targets lack accessible cysteines. This has prompted the development of warheads and labeling chemistries for targeting other residues, like lysine, histidine, tryptophan, methionine, tyrosine, serine, threonine, aspartate, and glutamate.^4,5^ While cysteine-labeling warheads are most often Michael acceptors, a wide range of warheads and chemistries, like other nucleophilic addition, addition–elimination, nucleophilic substitution, and oxidation reactions, have been shown to be suitable for covalent protein modification^6^ Labeling chemistries and warheads impact the reactivity and selectivity of the covalent fragments. Surrogate reactivity and selectivity models can be used to characterize and select the appropriate warheads. The limitation of this approach, however, is that reactivity is determined by prereaction and transition state geometries and energies, which may differ in the surrogate reaction compared to the nucleophile labeling in the protein environment.

**Figure 1:**
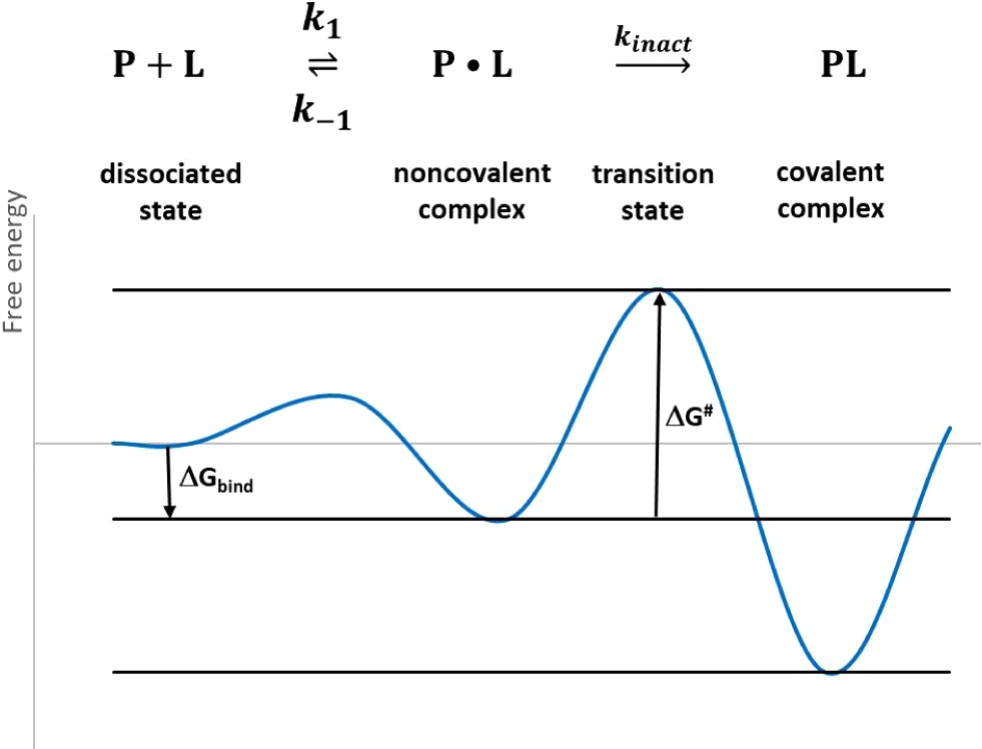
Two steps of covalent ligand binding and its schematic free energy profile.

The binding of covalent ligands to proteins is most often described as a two-step process (Figure 1). First, the ligand (L) associates with the protein (P) through noncovalent interactions. This recognition process results in a complex (P·L) where the warhead of the ligand and the reactive group of the protein are in proximity, which is necessary for the subsequent second step, the chemical reaction that leads to the covalent complex (PL).

The schematic free energy diagram of the binding is also shown in Figure 1. The noncovalent recognition step can be characterized by the dissociation constant of the complex , which relates to the noncovalent binding free energy . Alternatively, the *K*_I_ inhibition constant is used, which is defined as  when a constant concentration of the noncovalent complex is assumed (). *K*_*I*_ is well approximated by *K*_d_ (often designated by *K*_i_) when the dissociation rate constant of the noncovalent complex, *k*_–1_, is much higher than the inactivation rate constant, *k*_inact_, or, in other words, the noncovalent complex formation is a fast equilibrium process preceding the slower chemical reaction. The chemical bond formation may be either reversible or irreversible, and the latter is far more common among the covalent ligands applied. The irreversible chemical reaction is characterized by the inactivation rate constant, *k*_inact_, which relates to the free energy barrier of the covalent step . Both *k*_inact_ and *K*_I_ can be experimentally determined, and they can be calculated as discussed in detail later. The rate of covalent modification can be characterized for small ligand concentrations by *k*_inact_*/K*_I_ (see below).

The IC_50_s of covalent inhibitors are time-dependent and are not considered as an optimal measure of activity. However, IC_50_s measured in identical conditions are proportional to  and, therefore, they can be used to compare covalent ligands assayed in a drug discovery project (eqs S5 and S8).^7^ Covalent labeling can be more precisely characterized by *k*_inact_ and *K*_I_ that are obtained by fitting to experimentally determined data, like occupancy or IC_50_ (eqs S1, S4, and S7). The fitting is typically performed without investigating if the covalent binding follows the two-step model (Figure 1). However, both the noncovalent binding and the subsequent chemical reaction may follow mechanisms that deviate from the two-step model. When slow binding or tight binding occurs in the noncovalent step, neither equilibrium between the noncovalent complex and the dissociated species nor constant concentration of the noncovalent complex can be assumed to define *K*_I_. Moreover, the labeling reaction may follow complex mechanisms, and the rate-determining step or steps may not be easily identified. Multilevel calculations reported for several systems have suggested multistep chemical reactions for the covalent bond formation involving Michael acceptors.^8^ Depending on the investigated system, either direct addition of the cysteine thiol group^9^ or deprotonation prior to thiolate addition^10^ has been proposed. In some other calculations, the deprotonation step was not explicitly considered; rather, the deprotonated thiolate form was assumed a *priori*.^11−13^ When the labeling reaction proceeds via multiple steps with comparable free energy barriers, the rate constants of the elementary steps contribute to the observed apparent *k*_inact_. Still, apparent *k*_inact_ and *K*_I_ can be used to fit experimental data (eqs S1, S2, S4, and S7) and to characterize the irreversible inhibition. The situation is different when *k*_inact_ and *K*_I_ are not derived from experiments but are calculated from an assumed mechanism. Focusing on the chemical reaction, we have to identify the rate-determining step or steps in order to calculate expected observables like occupancy, which may depend on the rate constants of multiple steps.

The effectiveness of both target and ligand discovery processes is typically measured by their hit rate, which is directly connected to the labeling efficiency of the screened covalent fragments. Our objective, therefore, is to characterize the activity of covalent ligands toward a protein target so that their labeling efficiency can be foreseen. We investigate how detectable labeling depends on the recognition process and the chemical bond formation, together with the properties of the labeled residue and the detection method. The results presented here are useful for designing effective covalent libraries for both target and ligand discovery applications.

## Results and Discussion

### Mechanistic Enzymology of Covalent Labeling

Although covalent labeling is most intensively used in the field of enzyme inhibition, focusing on cysteine residues, the labeling of a wide range of other proteins^14,15^ and other residues^4,5^ has also been reported. Therefore, the results below are relevant for both covalent enzyme inhibition and the labeling of proteins with other functions. In the following discussion, we focus on cysteine targeting; however, any targeted protein nucleophile can be treated in a similar way.

We performed simulations (eqs S1 and S2) to show how occupancy is affected by *k*_inact_, *K*_I_, and [*L*] for the two-step mechanism (Figure 1) and a selected three-step mechanism, where ligand binding is followed by the activation of the complex. An example of such a three-step process is the ligand binding to the protein to form a noncovalent complex (1st step), the deprotonation of the cysteine thiol to form a reactive thiolate (2nd step), and the attack of the thiolate on the Michael acceptor ligand (3rd step). This may be followed by other steps that are fast enough to not affect the overall reaction rate.

**Figure 2:**
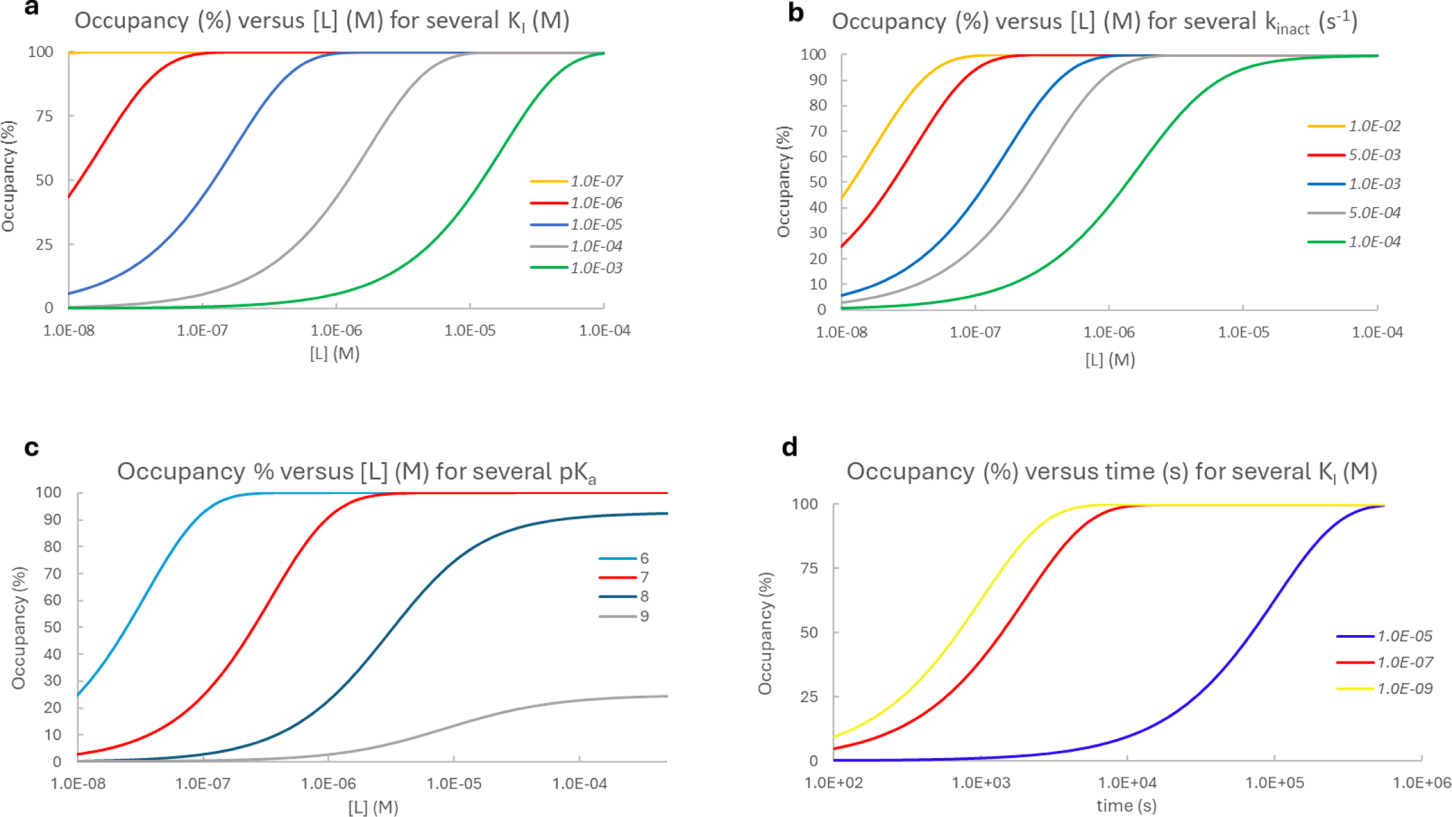
Simulation of occupancy % versus ligand concentration ([*L*]) or incubation time (*t*): (a) Occupancy % versus ligand concentration ([*L*]) for two-step mechanism with *k*_inact_ = 10^–3^ s^–1^, *t* = 16 h, and several *K*_I_ (M). (b) Occupancy % versus ligand concentration ([*L*]) for two-step mechanism with *K*_I_ = 10^–5^ M, *t* = 16 h, and several *k*_inact_ (s^–1^). (c) Occupancy % versus ligand concentration ([*L*]) for three-step mechanism where ligand binding precedes deprotonation of the reactive residue. Curves calculated for several p*K*_a_ values using *k*_inact_ = 5·10^–4^ s^–1^, *K*_1_ = 10^–5^ M, *t* = 16 h, and pH = 7. (d) Occupancy % versus incubation time (*t*) for two-step mechanism with *k*_inact_ = 10^–3^ M, [*L*] = 10^–7^ M, and several *K*_I_ (M).

The simulations show that increased occupancy is achieved with a certain combination of parameters. For example, Figure 2a shows that an inhibitor with *k*_*inact*_ = 10^–3^ s^–1^ and a noncovalent affinity (*K*_I_) of 100 μM requires micromolar concentrations to achieve around 50% occupancy with 16 h incubation, while a 100 nM concentration reduces the occupancy to a few percent (Figure 2a gray curve). The effect of reactivity is illustrated in Figure 2b, showing that an inhibitor with 10 μM noncovalent affinity needs increasing concentrations as reactivity decreases. 50% occupancy is achieved with a concentration above 1 μM for an inhibitor having *k*_inact_ = 10^–4^ s^–1^ (Figure 2b green curve), and the inhibitor concentration can be reduced to the 100 nM range with increased reactivity of *k*_inact_ = 10^–3^ s^–1^ (Figure 2b blue curve). When ligand binding is followed by the deprotonation of the targeted cysteine residue, the p*K*_a_ of the cysteine affects the occupancy, as illustrated in Figure 2c. 50% occupancy cannot be achieved even with micromolar inhibitor concentrations with p*K*_a_ = 8 or 9, typical for noncatalytic cysteines^16^ (Figure 2c gray and blue curves) under the applied conditions (*k*_inact_ = 5·10^–4^ s^–1^, *K*_I_ = 10^–5^ M, pH = 7, and *t* = 16 h). In contrast, when p*K*_a_ is reduced to 6, almost full occupancy is achieved with a 100 nM concentration (Figure 2c gold curve). Besides ligand concentration, incubation time can also be varied, and its effect on occupancy is shown in Figure 2d. 50% occupancy can be achieved within a few minutes of incubation for compounds with high noncovalent affinity (K_*I*_ = 10^–9^ M, achievable with drug-like compounds), while the required incubation time increases with decreasing affinity and amounts to several hours for K_*I*_ = 10^–5^ M, typical for fragment-sized compounds.

Occupancy depends on multiple parameters even in the simplest model, including two steps and no competing ligand. These parameters are the inhibition constant, *K*_I_, inactivation rate constant, *k*_inact_, ligand concentration, [*L*], and incubation time, *t*. Various combinations of these parameters may lead to significant labeling, as shown in Figure 2a,b. While incubation time and ligand concentration can typically be varied over a wide range, *K*_I_ and *k*_inact_ are characteristics of the system. In the following sections, we analyze how experimental data and design can support the selection of compounds with an optimal range of *K*_I_ and *k*_inact_ to compile compound collections well-suited for target identification proteomic studies and ligand discovery.

### Optimization of Target Occupancy through the Inactivation Rate Constant, *k*_inact_, of the Covalent Step

The rate constant of the chemical reaction between the ligand and the protein can be estimated by both computational and experimental tools. The computational evaluation of the free energy barrier of covalent bond formation can be performed by mixed quantum mechanical and molecular mechanical simulations when the mechanism of the chemical reaction is known. Alternatively, the quantum mechanical free energy barrier of a model reaction mimicking that between the ligand and the targeted residue can be used to estimate the inactivation rate constant. The experimental determination of the rate constant of a model reaction is also a widely applied option. Considering cysteine as a target, reactivity assays against various thiol surrogates, like cysteamine, cysteine, glutathione (GSH), and others, have been used to characterize the intrinsic reactivity of ligands. Among these, GSH is the most frequently used, justified by its human relevance and its ability to provide a good estimate of reactivity against proteins.^17^ In addition, GSH reactivities have been shown to be well estimated by quantum chemical methods.^18−20^ An alternative approach is the high throughput reactivity characterization using TNB^2–^, reduced DTNB (Ellman’s reagent; 5,5-dithio-bis-2-nitrobenzoic acid), as applied to a library of nearly 1000 electrophilic compounds.^2^ The labeling of several proteins by compounds characterized by either GSH or TNB^2–^ reactivities has been investigated, and no correlation between reactivity and overall protein labeling was reported.^2,21^ By contrast, an enrichment of high-reactivity compounds among covalent hits was reported in a screening of fragment-sized molecules.^22^

Although reactivity is expected to increase the labeling efficiency, there are multiple factors that may obscure the relationship between compound reactivity and labeling. These factors include the potentially different mechanisms of the model reactions compared to the protein labeling reactions and the context-dependent reactivity of the nucleophilic residues. Even if the mechanism of the model reaction and the labeling reaction agrees, as is expected for nucleophilic substitution between cysteine and a chloroacetamide warhead, both the enthalpic and entropic components of the reaction free energy barrier may differ, as the reactant state for the ligand-protein system is the noncovalent complex that is not present in the model reaction. Moreover, labeling efficiency is also affected by the noncovalent recognition step (Figure 2a) that is protein- and ligand-dependent but independent of reactivity. The variation of the inactivation rate constant in the protein environment is well illustrated by the *k*_inact_ values measured for the same ligand in multiple proteins (Table S2).

We further analyzed the data in ref (2) as the reactivity of nearly 1000 fragments and their labeling efficiency against ten targets offers a unique opportunity to gain further insight into the relationship between reactivity and labeling. Plots of percent labeling as a function of reactivity for each target (Figure S1) show that labeling tends to increase with reactivity for the top labeling compounds. This finding can be interpreted by assuming that compounds showing the highest labeling for a given reactivity are those that have high-affinity noncovalent complexes, and owing to their similar size and the reasonable coverage of the fragment chemical space, their affinities are comparable. Then, the contribution of the noncovalent recognition to labeling is similar, and labeling tends to increase with reactivity, as observed for seven out of the ten targets. Labeling was under 50% for the remaining three targets, suggesting that either low-affinity noncovalent association or low cysteine reactivity prevents efficient labeling. In addition to the labeling-reactivity relationship for top labeling compounds, we found that the labeling averaged for compounds with the same reactivity also tends to increase with reactivity for the majority of the targets investigated. Moreover, labeling averaged for the targets also tends to increase with reactivity (Figure S2). We suggest that the relationship between average labeling and reactivity is found owing to the large number of compounds involved, as the varying affinity of their noncovalent recognition largely cancels out for the average labeling.

The recognition of the relationship between reactivity and labeling was attributed to the canceling contribution of noncovalent affinity either because they have similar values, as assumed for the top labeling compounds, or because they are averaged for a large number of compounds. We further investigated the reactivity-labeling relationship using data obtained for a set of ultralow molecular weight compounds. These mini-fragments are heterocyclic compounds substituted with a halogen atom or a small unsaturated group.^23,24^ The labeling efficiency of these small and reactive compounds is expected to be primarily determined by their reactivity and less by their noncovalent recognition, since their very small size prevents significant contributions from noncovalent affinity. The GSH reactivity of 58 mini-fragments and their labeling of 7 targets were investigated, and it was found that the labeling averaged over the 7 targets increases with increasing compound reactivity (Table S1 and Figure S3). These data also show that there is significant variation in the labeling efficiency of compounds with similar reactivity. This indicates a non-negligible contribution from noncovalent interactions; nevertheless, the increase in the averaged labeling with reactivity shows that the binding of these small fragments is primarily reactivity-driven.

Ligand reactivity has a pronounced effect on the success of cell-based chemoproteomic studies aiming to identify ligandable residues. The direct relationship between intrinsic ligand reactivity and labeling efficiency is blurred by the large number of protein nucleophiles showing ligand-dependent reactivity; nevertheless, a tendency for more effective labeling by more reactive warheads was identified in proteomic studies using libraries of small compounds. Higher labeling by the more reactive chloroacetamides compared to acrylamides was observed in a cell-based screening of an electrophilic library^25^ (see Figure S4). Labeling of 758 proteins by an electrophilic compound collection was reported,^21^ and we analyzed the distribution of labeling efficiency of compound pairs with the same scaffold and with either an acrylamide or chloroacetamide warhead. The distributions clearly show more effective labeling by the more reactive chloroacetamide compounds (Figure S5). Similarly, it was reported for aminophilic electrophiles that compounds with strong reactivity toward a model compound tend to show strong proteomic reactivity^26^ (see Figure S6).

It has to be emphasized that the above findings do not imply a general correlation between the surrogate reactivity and protein labeling. In contrast, labeling is affected by noncovalent recognition and the presentation of the warhead to the protein nucleophile. What these analyses show is that reactivity contributes to labeling when the effects of other factors cancel out. In typical applications of covalent labeling in ligand discovery or protein targetability investigations, we look for compounds with notable contributions from noncovalent affinity and limited contributions from the chemical reaction, and we have to be aware of the demonstrated effect of reactivity. Although the estimation of *k*_inact_ from reactivities measured in model reactions is not feasible because *k*_inact_ is affected by the noncovalent recognition on the one hand, and by the mechanistic differences between the reactions with a surrogate molecule and a cysteine embedded in the protein on the other hand, reactivities obtained in model reactions are useful indicators of available inactivation rate constants when small compounds are investigated. Although these analyses focused on cysteine-targeting compounds, an analogous characterization of non-cysteine-labeling ligands against appropriate surrogates is also expected to provide information useful for estimating available inactivation rate constants. It is important to emphasize that increased labeling due to increased reactivity is not target-specific and thus acts against selectivity both among residues within a protein and among proteins.

### Optimization of Target Occupancy through the Inhibition constant (*K*_I_) of the Noncovalent Step

**Figure 3:**
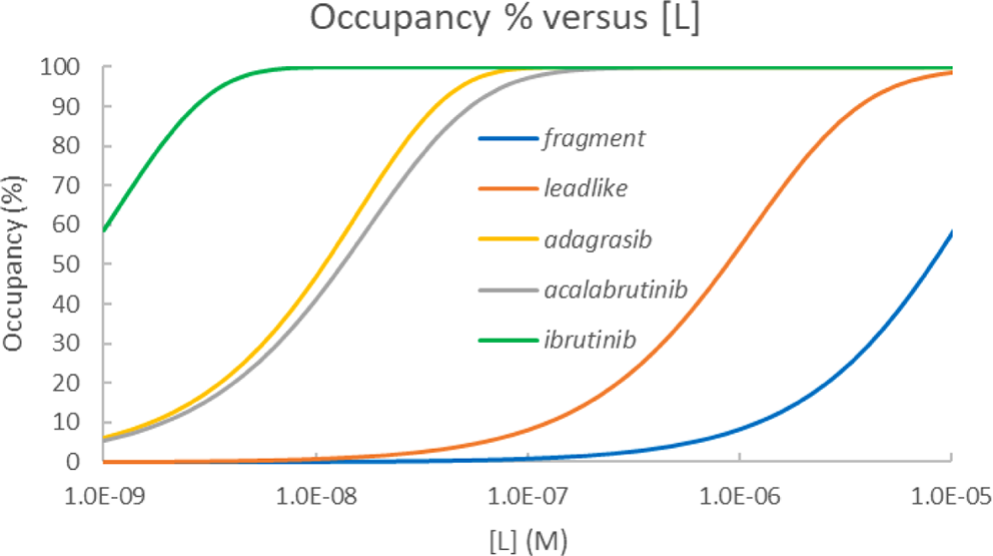
Simulation of occupancy % versus ligand concentration ([*L*]) for fragment (*K*_I_ = 10^–3^ M, *k*_inact_ = 10^–3^ s^–1^, and *t* = 24 h), lead-like (*K*_I_ = 10^–5^ M, *k*_*inact*_ = 10^–4^ s^–1^, and *t* = 24 h) and three drug (acalabrutinib *K*_I_ = 1.8·10^–7^ M, *k*_inact_ = 5.6·10^–3^ s^–1^, and *t* = 30 min; ibrutinib *K*_I_ = 5.4·10^–8^ M, *k*_inact_ = 2.7·10^–2^ s^–1^, and *t* = 30 min;^34^ adagrasib *K*_I_ = 3.7·10^–6^ M, *k*_inact_ = 1.3·10^–1^ s^–1^, and *t* = 30 min^35^) compounds.

The noncovalent recognition step is characterized by the inhibition constant, which is approximated by the equilibrium constant of the noncovalent complex formation when the chemical reaction of the covalent bond formation, the inactivation rate, is much slower than the dissociation of the noncovalent complex (see the discussion below Figure 1). In the following discussion, we accept this reasonable assumption and analyze K_*I*_ as an equilibrium constant. In this way, the noncovalent complex formation of the covalent labeling is described in the same way as the complex formation of noncovalent ligands. It is well established that the available affinity of noncovalent ligands is molecular size-dependent.^27,28^ This allows for the assignment of maximal inhibition constant ranges for fragment-sized, lead-like, and drug-like targeted covalent inhibitors. Accordingly, the occupancy simulation in Figure 3 used *K*_I_ = 10^–3^ M for the fragment, 10^–5^ M for lead-like, and 10^–7^ M for drug-like compounds. The *k*_inact_ value for the fragment was taken as 10^–3^ s^–1^. Here, we note that *k*_inact_ is not typically measured for fragments, and the selected value is in the medium-high range of those reported for larger compounds (see, e.g., refs (29−32)) and gives *k*_inact_/*K*_I_ = 1 M^–1^s^–1^ in line with the values observed for small compounds in ref (33) The *k*_inact_ of 10^–4^s^–1^ for the lead-like compound was chosen to yield a *k*_inact_/*K*_I_ = 10 M^–1^s^–1^ observed for lead-like compounds.^33^ The parameters for acalabrutinib^34^ and adagrasib^35^ were used to simulate drug-like occupancy.

Comparing the occupancy-ligand concentration curves in Figure 3, we see that 100% occupancy is achieved for all three compound types, although with different ligand concentrations and incubation times. Fragments require high ligand concentrations and long incubation times, as negligible occupancy is achieved up to micromolar concentrations after 24 h. Increasing reactivity allows the application of lower concentrations; however, it increases nonspecific labeling (cf. discussion on *k*_inact_). The increased labeling of lead-like compounds below micromolar concentrations with a modest inactivation rate constant shows that the size and associated noncovalent affinity are beneficial for lead-like compounds. Their labeling efficiency typically allows the variation of the measuring parameters in practically manageable ranges that include micromolar concentrations and a few hours of incubation. Drug-like compounds often exhibit high noncovalent affinity in the nanomolar range and an inactivation rate constant comparable to those of smaller compounds. They can achieve high occupancy at low concentrations and incubation times, which may hamper mass spectrometry-based *k*_inact_ and *K*_I_ determination and necessitate the use of biochemical assays. The high noncovalent affinity is a frequent feature of drug-like compounds; however, exceptions exist, like the approved KRAS^G12C^ inhibitors adagrasib and sotorasib, with *K*_I_ values of 3.7 μM^35^ and 12.2 μM,^36^ respectively. The low noncovalent affinity is accompanied by a higher inactivation rate constant, resulting in highly similar occupancy–ligand concentration curves for acalabrutinib and adagrasib (Figure 3). The selectivity of adagrasib^35^ shows that micromolar noncovalent affinity assures selective labeling even with an elevated inactivation rate constant of 0.13 s^–1^.

### Optimization of Target Occupancy through the pH Using the Three-Step Binding Model

**Figure 4:**
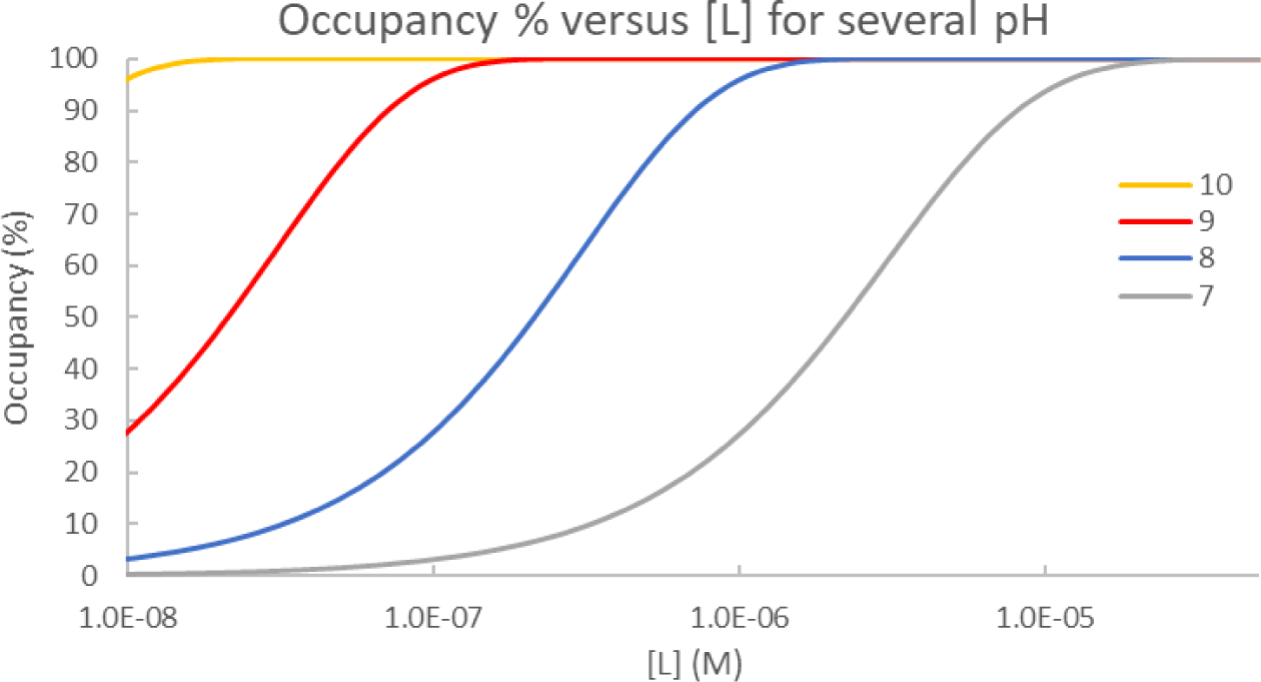
Simulation of occupancy % versus ligand concentration ([*L*]) for KRAS^G12C^ labeling by ARS-853 at several pH values using the three-step model of labeling (*K*_I_ = 5.62·10^–6^ M, p*K*_a_ = 9.2, *k*_inact_ = 0.80 s^–1^, and *t* = 1 h).

The formation of the covalent complex may follow a pathway more involved than the two-step binding, which includes the noncovalent complex formation and the chemical reaction for covalent bond formation. Both the ligand and the protein may be subject to an activation step, such as a conformational change or proton transfer. When such a step has a significant barrier, it may affect the observed first-order rate constant, *k*_obs_. One may still obtain *K*_I_ and *k*_inact_ from the two-step model (eq S2); however, they will not thoroughly describe the covalent labeling process. A typical example is the activation of a cysteine thiol by deprotonation to form a reactive thiolate. The p*K*_a_ of cysteines varies significantly in proteins, and pH changes near the cysteine p*K*_a_ affect the thiolate concentration and the observed first-order rate constant. This leads to pH-dependent *K*_I_ and *k*_inact_ when they are obtained from formulas corresponding to the two-step binding process (eq S2). As an example, we consider KRAS^G12C^ whose labeling has been investigated at various pH values for several ligands.^37,38^*k*_obs_ was measured for ARS-853 at pH values between 7 and 9, and *K*_I_ and *k*_inact_ were fitted using the two-step model (eq S2). Reported *K*_I_ values are in the 10 μM range, while *k*_inact_ increases with pH and shows an ∼40-fold variation in the pH range of 7–9 (Table S3). It is reasonable to assume that the source of pH dependence is the varying concentration of the reactive thiolate generated in a pH-dependent deprotonation of Cys12. Such covalent labeling can be described with a three-step model that includes the noncovalent ligand binding as the first step, Cys12 deprotonation of the complex as the second step, and the chemical reaction as the third step. The first step can be characterized by *K*_d_(≈ *K*_I_), the dissociation constant of the noncovalent complex, the second by the p*K*_a_ of Cys12, and the third by *k*_inact_. These are all independent of pH, which , in turn, affects labeling via the pH-dependent concentration of the noncovalent complex with deprotonated thiol (see “Three-step model of covalent labeling” in SI). Setting p*K*_a_ = 9.2 and fitting *k*_inact_ and *K*_I_ to the *k*_obs_*-*[*L*] function in the pH = 7–9 range (Figure S7) yields pH-independent  and  with mean values of 0.80 s^–1^ and 56.2 μM, respectively (Table S4). This *k*_inact_ is higher than the inactivation rate constant from the two-step model reported for any compound against KRAS^G12C^^35−38^ owing to the dependence of the latter on the thiolate concentration, which is low near neutral pH. The  and  parameters were used to calculate the pH-dependent occupancies (Figure 4).

Occupancies in Figure 4 show the expected pH dependence with the pH-independent parameters of the three-step model. Over micromolar ligand concentration is required for 50% occupancy at neutral pH, owing to the large difference between p*K*_a_ = 9.2 and pH = 7.0. Elevating the pH toward and above the cysteine p*K*_a_ decreases the ligand concentration needed for high occupancy. This behavior is typical in cysteine labeling systems, where a pH-dependent proton transfer affects the concentration of the reactive species, and is also relevant for other protein nucleophiles with protonation state-dependent reactivity, like serine, cysteine, lysine, tyrosine, threonine, aspartate, and glutamate. The pH-dependent labeling can be quantitatively described with the pH-independent parameters of a three-step model that includes the proton transfer in addition to the noncovalent complex formation and the chemical reaction of covalent bond formation.

### Experimental Validation

Two validated oncogenic protein targets, Bruton’s tyrosine kinase (BTK) and KRAS^G12C^, along with their irreversible covalent inhibitors, were selected for studies illustrating the effect of ligand size, noncovalent recognition, and covalent reactivity on affinity. BTK is a TEC-family nonreceptor protein kinase^39^ with approved covalent inhibitors ibrutinib (also known as PCI-32765)^40^ and acalabrutinib (also known as ACP-196).^34^ KRAS^G12C^ is an oncogenic mutant of KRAS, a member of the small membrane-bound Ras protein, and acts as a molecular switch. Its G12C mutation impairs GTPase-activating protein binding and yields a hyperactivated protein with constant activation of downstream signaling pathways.^41,42^ Adagrasib (also known as MRTX849) is an approved, highly selective, and potent small-molecule irreversible inhibitor of KRAS^G12C^.^35,43^ In addition to the approved drugs, we also investigated their lead-like and fragment-sized variants derived from the original compounds by successively removing structural elements while retaining their warhead (Tables 1–3). In addition, for all three drug molecules, we modified the warhead to include the generally more reactive chloroacetamide, and these molecules were used as starting points to derive lead-like and fragment-sized compounds (Tables 1–3).

**Table 1:**
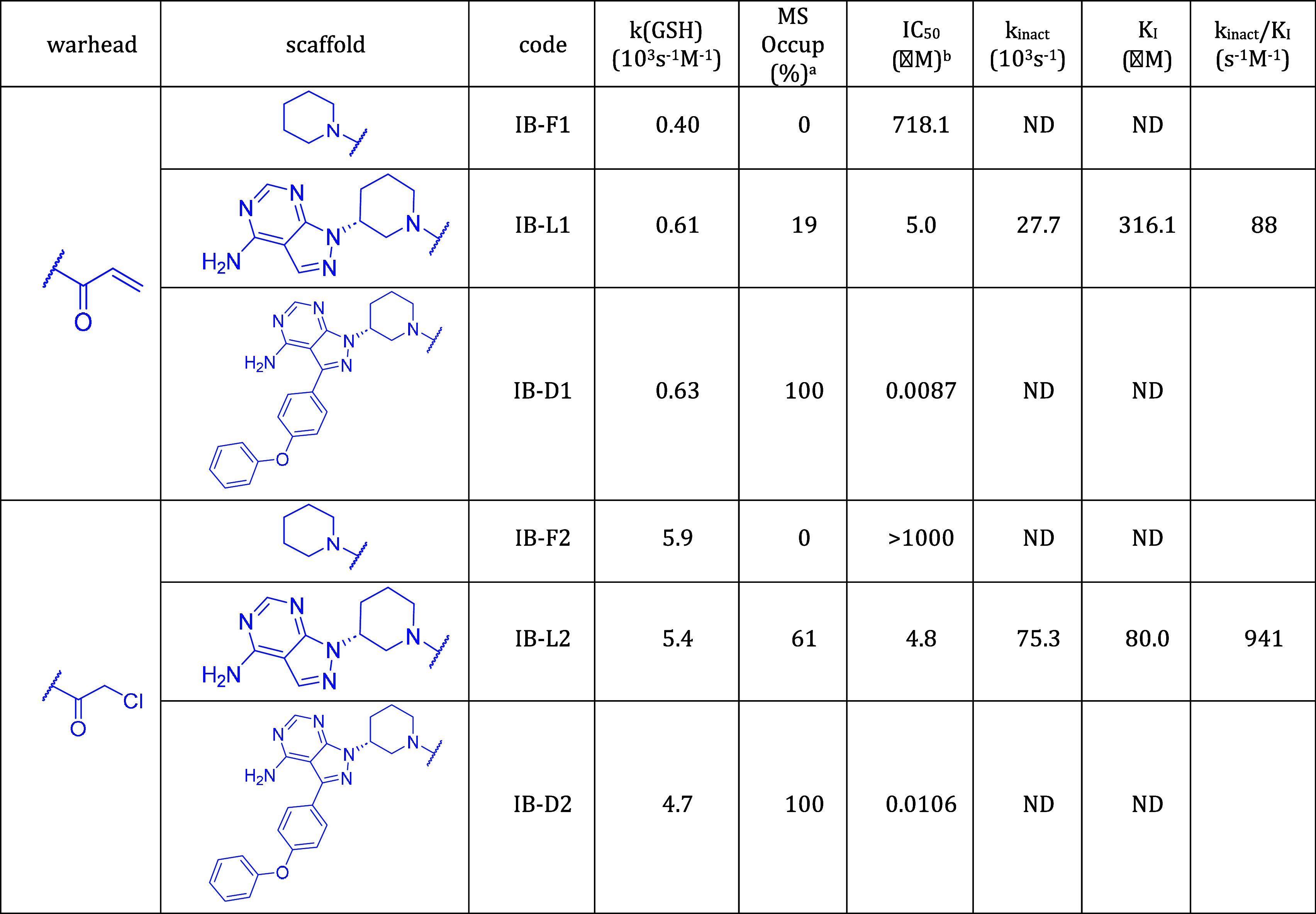
Activity-Related Data of Compounds Derived from Ibrutinib; Target: BTK

**Table 2:**
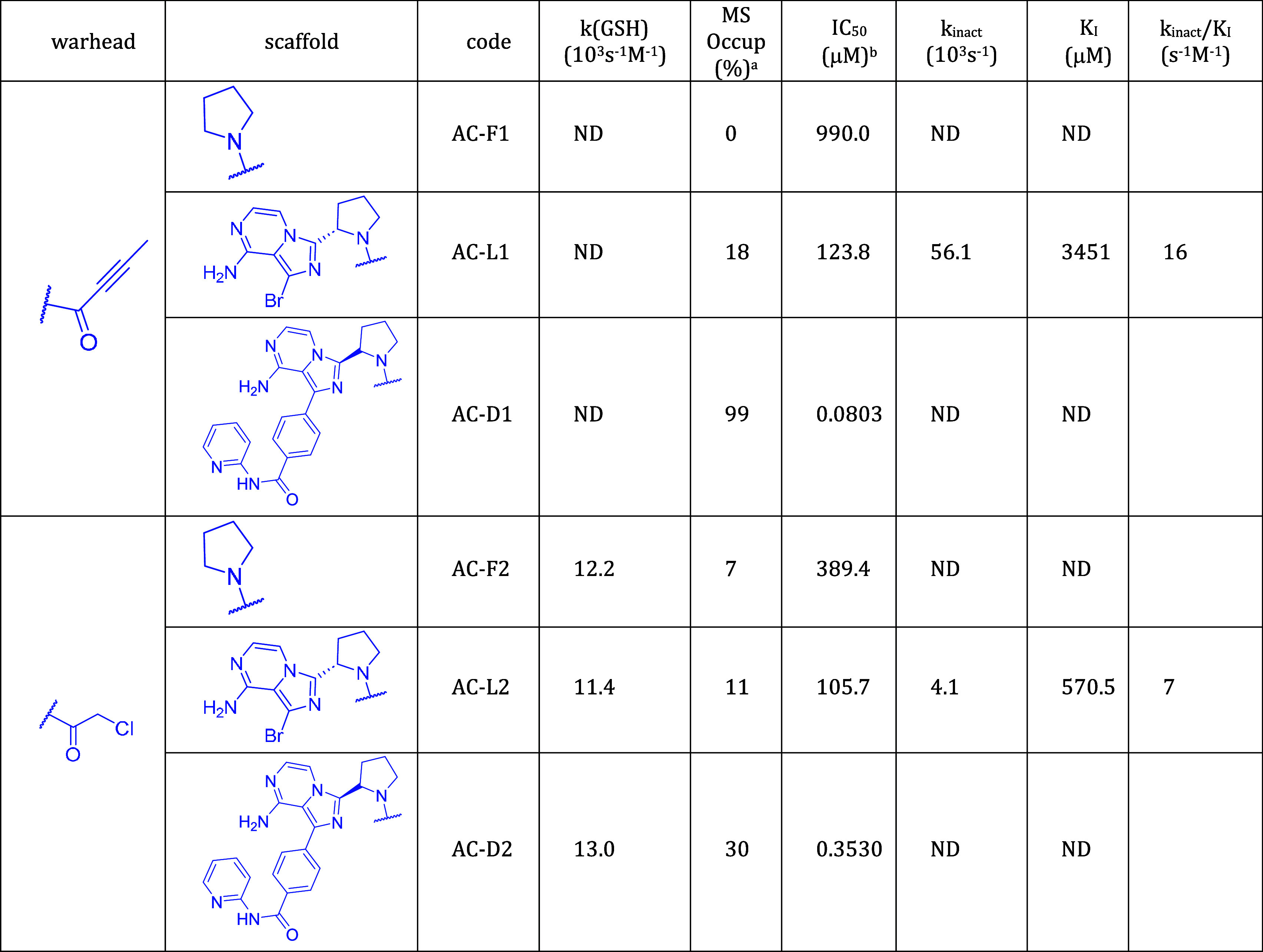
Activity-Related Data of Compounds Derived from Acalarutinib; Target: BTK

**Table 3:**
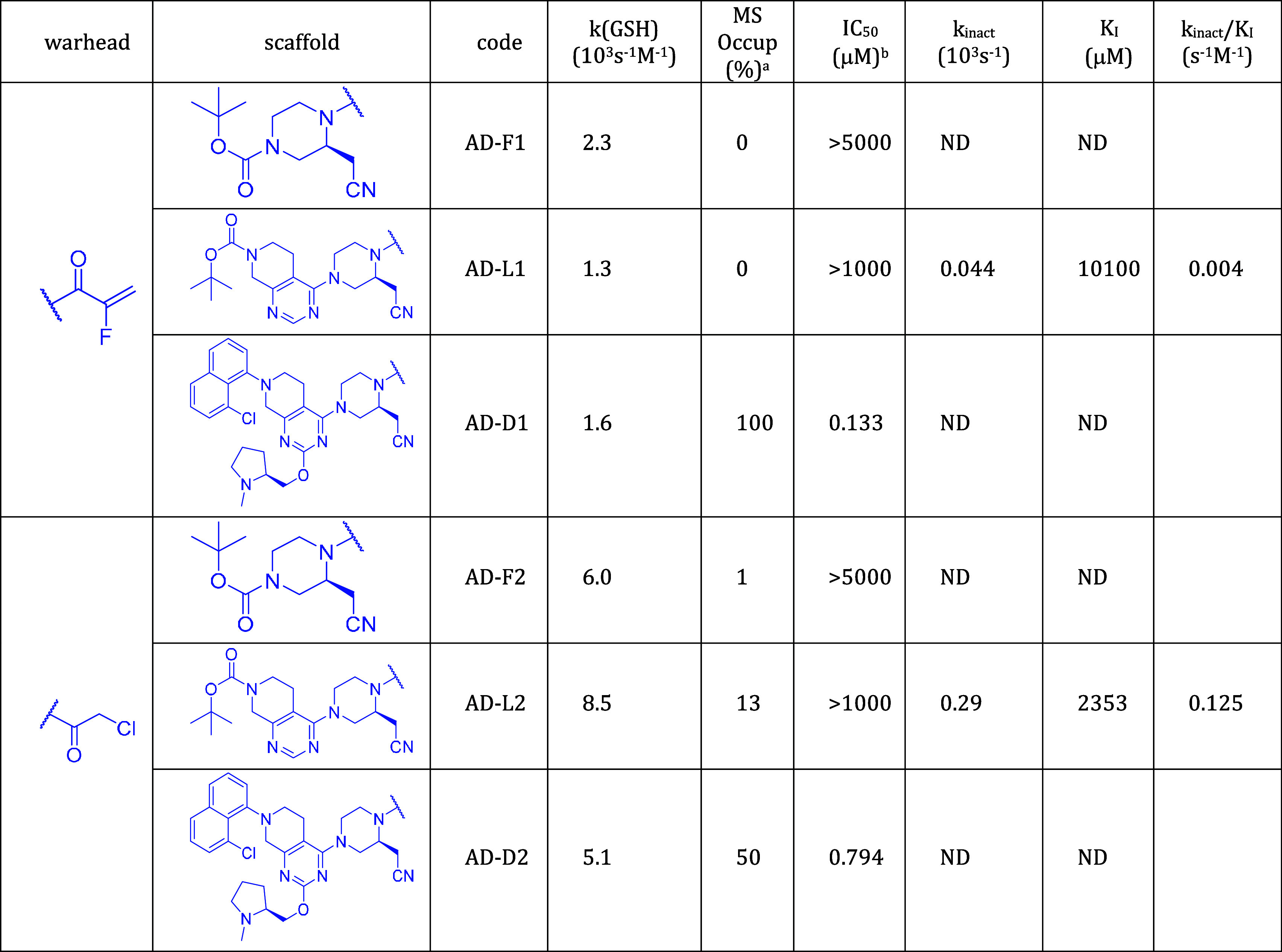
Activity-Related Data of Compounds Derived from Adagrasib; Target: KRAS^G12C^

Ibrutinib was developed from a noncovalent nanomolar inhibitor of BTK by introducing a warhead to target Cys481 and improve selectivity.^44^ Acalabrutinib is a second-generation BTK inhibitor with reduced intrinsic reactivity and higher biochemical and cellular selectivity.^34^ The detailed mechanism of BTK covalent labeling by ibrutinib and acalabrutinib was explored by quantum mechanical/molecular mechanical calculations. The ibrutinib reaction was proposed to start with a direct proton transfer from the Cys481 of the catalytic pocket to the acrylamide warhead, followed by S–C bond formation. The enolate intermediate undergoes a solvent-assisted tautomerization, which was found to be the rate-determining step of the covalent labeling.^45^ A similar mechanism, but with comparable barriers between the nucleophilic attack and the tautomerization, was obtained in another computational study on the ibrutinib-BTK reaction mechanism.^46^ Acalabrutinib was proposed to react with a water-assisted proton transfer coupled with the nucleophilic attack, and these steps have a higher free energy barrier than the subsequent water-assisted tautomerization.^47^

The KRAS^G12C^ inhibitor adagrasib was discovered by a different route. While KRAS^G12C^ was long considered undruggable, the screening of tethering compounds led to the identification of inhibitors that covalently bind to Cys12 in a previously unseen pocket.^48^ Analogous screening and subsequent optimization of the scaffold and the warhead led to the discovery of adagrasib.^43^ This is not the typical ligand-first approach, which introduces the warhead on a potent noncovalent inhibitor, as was done with ibrutinib. Rather, a modest-sized covalent ligand in a new binding site was first identified and developed into a drug.

The mechanism of the chemical reaction between KRAS^G12C^ and adagrasib has not been investigated to the best of our knowledge, but the mechanism of other α,β-unsaturated carbonyl compounds was studied by QM/MM calculations. There is no obvious proton acceptor to activate Cys12, and there are conflicting estimates for its p*K*_a_ based on pH-dependent reactivity and NMR measurements.^37,38^ Concerted proton transfer to the warhead carbonyl group and a nucleophilic attack on the β-carbon were proposed as the first step of the reaction.^49^ The resulting enolate intermediate undergoes solvent-assisted tautomerization in the rate-determining step. An alternative mechanism was proposed for ARS-853, an aliphatic acrylamide derivative, with a thiolate attack on the β-carbon followed by the rate-determining proton transfer from a water molecule coordinating a magnesium cation.^50^

First, we assessed the intrinsic reactivity of the compounds. Reactivities measured against GSH are consistent within each fragment-lead-drug triplet of compounds. This is in line with the expectation that GSH reactivity primarily depends on the warhead and is not significantly affected by the scaffold, as molecular recognition does not play an important role in the reaction against GSH. Compounds with a chloroacetamide warhead are more reactive than compounds with acrylamide warhead, and compounds with a 2-butynamide warhead do not react with GSH under the applied conditions.

**Figure 5:**
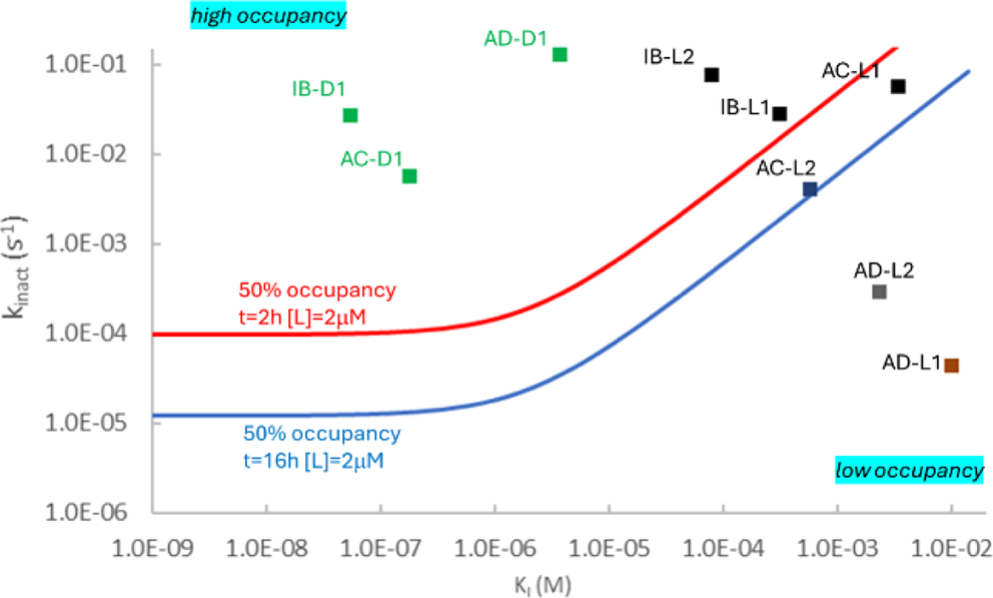
*K*_I_ and *k*_inact_ are for compounds in Tables 1–3. Lead-like compounds are shown with black squares. Drugs are shown with green squares with *K*_I_ and *k*_inact_ taken from refs (34) and (35). Occupancy increases from bottom right to top left. 50% occupancies calculated with parameters [*L*] = 2 μM, *t* = 2 h, and [*L*] = 2 μM, *t* = 16 h applied for determining occupancies in Tables 1–3 are indicated.

Next, we characterized covalent labeling by MS-determined occupancy and IC_50_ values. In addition, *k*_inact_ and *K*_I_ for the lead-like compounds were also measured. At this point, two notes are appropriate. First, occupancy, IC_50_ and kinetic parameters *k*_inact_ and *K*_I_ represent increasingly thorough characterizations of the inhibitors. Second, BTK and KRAS^G12C^ inhibitors have some distinctive features. Efficient recognition with nanomolar *K*_I_ can be achieved with BTK inhibitors, while no lower than micromolar *K*_I_ values have been reported for KRAS^G12C^ inhibitors. The separation of *k*_inact_ for BTK and KRAS^G12C^ inhibitors is less apparent, although *k*_inact_ = 0.13 s^–1^ of the KRAS^G12C^ inhibitor adagrasib^35^ exceeds those reported for BTK inhibitors.^34^ Another difference is that while BTK inhibitors are tested in an enzyme inhibition assay consistent with the equations of the two-step model (eqs S3–S8), the Michaelis–Menten kinetics is not straightforwardly applied to the exchange assay of KRAS^G12C^ inhibition. Nevertheless, the linear relationship between IC_50_ and *K*_I_/*k*_inact_ (eqs S5 and S8) was also found for KRAS^G12C^ inhibitors (Table S5 and Figure S8). The *K*_*I*_ and *k*_inact_ values are shown in Figure 5 to illustrate how these parameters affect occupancies.

All fragments have occupancies close to zero, and their IC_50_s approach or exceed millimolar, indicating that detecting their binding is difficult. Considering the drug-like compounds, it is worth separating fully optimized drugs with those derived from them by warhead exchange. All drugs show full occupancy and submicromolar IC_50_. Replacing their warhead by chloroacetamide yields slightly increased IC_50_ for all three compounds and substantially decreased occupancy for AC-D2 (30%) and AD-D2 (50%), but not for IB-D2 (100%) which has a lower IC_50_ than the other two compounds. It is interesting to see that although chloroacetamide is more reactive than the drug warheads acrylamide (ibrutinib), 2-butynamide (acalabrutinib), and 2-fluoroacrylamide (adagrasib) (cf. *k* (GSH) values in Tables 1–3), nevertheless, the biochemical activity and occupancy are lower for the compounds with chloroacetamide warhead. This may be the result of less efficient recognition (higher K_*I*_) and a slower chemical reaction (lower *k*_inact_). The latter is affected by the geometrical fit of the warhead and the protein nucleophile, which is expected to be less favorable compared to the optimized drug molecule. This may lead to a lower *k*_inact_ in spite of the higher GSH reactivity of compounds with the chloroacetamide warhead.

Lead-like compounds are between fragments and drugs in terms of size, biological activity, and labeling efficiency. The reduced-size lead-like KRAS^G12C^ inhibitors (AD-L1 and AD-L2) exhibit very low noncovalent affinity (high *K*_I_) and low reactivity (low *k*_inact_); the latter can be attributed to the suboptimal presentation of the warhead to Cys12. In contrast, investigated lead-like BTK inhibitors show intermediate occupancy in the 11–61% range and single- to triple-digit nanomolar biochemical activity. Their activities are separated by scaffold; ibrutinib-derived lead-like molecules have higher activity compared to acalabrutinib-derived molecules (IC_50_: IB-L1 5.0 μM, IB-L2 4.8 μM, AC-L1 123.8 μM, and AC-L2 105.7 μM). This activity separation also appears in the *k*_inact_*/K*_I_ ratio (IB-L1 88 M^–1^s^–1^, IB-L2 941 M^–1^s^–1^, AC-L1 16 M^–1^s^–1^, AC-L2 7 M^–1^s^–1^) and in the *K*_I_ values (IB-L1 316.1 μM, IB-L2 80 μM, AC-L1 3451 μM, AC-L2 570.5 μM). The separation of the *K*_*I*_ values by scaffold is in accordance with the expectation that the noncovalent recognition of lead-like compounds is primarily governed by their scaffold, and the warhead has a less important contribution. This feature is preserved for the drug-like BTK inhibitors, as compounds with the ibrutinib scaffold have higher biochemical activity compared to compounds with the acalabrutinib scaffold (IC_50_: IB-D1 0.0087 μM, IB-D2 0.0106 μM, AC-D1 0.0803 μM, AC-D2 0.353 μM). In contrast, activity separation by scaffolds cannot be recognized at the fragment level (IC_50_: IB-F1 718.1 μM, IB-F2 > 1000 μM, AC-F1 990 μM, AC-F2 389.4 μM), showing that noncovalent recognition is not the major determinant of activity. The fragments investigated are derived from drugs by retaining their warhead and their immediate environment (IB-F1, AC-F1, AD-F1) or derived from these fragments by warhead replacement (IB-F1, AC-F1, AD-F1). Therefore, they do not contain the recognition motifs providing significant noncovalent affinity to the drugs. This explains why their binding (either covalent or noncovalent) and their inhibitory activity are very low or undetectable. This clearly shows the difficulty in developing the investigated drug molecules from a fragment-sized covalent warhead. Moreover, even if the binding of the covalent fragment is detected, optimization by fragment growing improves the noncovalent interactions that may affect the binding mode^22^ and complicate optimization. In contrast, lead-like BTK inhibitors (IB-L1, IB-L2, AC-L1, AC-L2) show micromolar activity with significant contribution from noncovalent recognition quantified by *K*_I_. This ensures detectable occupancy and inhibitory activity and increases the probability of a fixed binding mode that is preserved during optimization of lead-like compounds.^33,51^ Indeed, the clinically tested KRAS^G12C^ inhibitor ARS-1620^52^ and its predecessor ARS-853^53^ were developed from smaller covalent binders; however, both the initial hit found by disulfide tethering and its acrylamide analog used for further development have more than 20 heavy atoms and establish noncovalent interactions with the binding pocket, as shown by their X-ray structures.^48^ AMG-510 (Sotorasib), another clinically tested KRAS^G12C^ inhibitor, was also optimized from a lead-like covalent screening hit, which has 29 heavy atoms and extensive noncovalent interactions with the protein.^51,54^

The electrophile-first approach identifies covalent binders that are optimized into covalent drugs. In addition to KRAS^G12C^, this approach was successful against other targets, including the SARS-CoV-2 main protease, hepatitis C virus NS3/4a protease, and 26S proteasome,^55^ all starting from lead-like covalent binders. The recently reported VVD-133214, a clinical-stage WRN helicase inhibitor, was developed from a covalent hit compound of over 20 heavy atoms.^56^ The hit was identified in a chemoproteomic screen, and the optimization retained the warhead while varying the decoration of the skeleton, with a modest increase in molecular size (23–30 heavy atoms). VVD-130037, a clinically tested KEAP1 inhibitor, was optimized from a chemoproteomic screening hit^57^ but no information on its development released.

## Conclusions

The covalent labeling of protein targets is most often described by the two-step model, which includes the noncovalent complex formation and the covalent reaction forming the chemical bond between the ligand and the protein. The first step depends on the noncovalent recognition, characterized by the *K*_I_ inhibition constant, which is well approximated by the equilibrium constant of the noncovalent complex. The second step with the chemical bond formation, characterized by the *k*_inact_ inactivation rate constant. Other factors affecting the covalent occupancy of the target are ligand concentration and incubation time, and a proper combination of the four parameters is needed to achieve significant labeling. To maximize the practical utility of our simulations and findings in library and ligand design, we suggest the prediction of ligand occupancy. Occupancy can be calculated as a function of these parameters, offering the opportunity to properly select experimental conditions, such as ligand concentration and incubation time, for detectable labeling. Although *k*_inact_ and *K*_I_ can be estimated using resource-intensive computations, their large-scale accurate prediction is currently not feasible. Nevertheless, ligand size-based estimation of attainable *K*_I_ values suggests the use of 10^–4^–10^–5^ M for fragments, 10^–6^–10^–7^ M for lead-like, and 10^–8^–10^–9^ M for drug-like compounds.^58,59^ We suggest the use of *k*_inact_ in the 10^–2^–10^–4^ s^–1^ range that covers the majority of the experimentally determined values. When selecting warhead reactivity, one has to keep in mind that model reactivities of useful warheads show modest variation (around 2 orders of magnitude in the first-order rate constant).^6,19^ They depend on the model reaction, and for small compounds, they tend to increase together with protein reactivity.

The covalent labeling process may include more than two elementary steps, as it often contains an activation step, such as the deprotonation of the cysteine residue. The *K*_I_ and *k*_inact_ parameters, with an adapted interpretation, still can be applied to model covalent labeling, but they are unable to account for the pH dependence of the process. It was shown for pH-dependent KRAS^G12C^ labeling that the occupancy can be properly described with a three-step model that uses pH-independent parameters.

While ligand reactivity is often characterized in a model reaction, a direct relationship between such reactivity and *k*_inact_ is blurred by potentially different mechanisms of the model reaction and the covalent bond formation within the protein, the presence of the noncovalent complex as the reactant state in the protein but not in the model reaction, and the contribution of noncovalent affinity (effect of *K*_I_) to the labeling efficiency. We showed that the effect of noncovalent affinity is reduced when using fragment-sized compounds, whose labeling efficiency tends to increase with the reactivity of the ligands as obtained in model reactions. This provides a rationale for using small, reactive compounds for proteomics applications, as they are able to label protein nucleophiles and map their reactivities.^21,25,60−65^

Different considerations apply when covalent binders are screened to find ligands with the potential to be developed into therapeutic agents. Drug-like targeted covalent inhibitors (TCIs) bind with significant noncovalent affinity needed to achieve selectivity. The recognition motifs responsible for the noncovalent affinity must be at least partially present in the chemical starting point identified by screening to ensure smooth optimization of the screening hit into a TCI. As fragment-sized compounds are unable to provide high noncovalent affinity, their labeling highly relies upon their reactivity. They typically do not contain the key recognition motif, whose inclusion upon optimization may change the binding mode and the presentation of the warhead to the protein nucleophile. This may necessitate warhead replacement upon fragment growth, not to reduce reactivity but to ensure covalent labeling by appropriately positioning the warhead. Altogether, fragment-sized covalent agents do not offer smooth optimization and are not ideal starting points for TCI development. In contrast, compounds with significant noncovalent interactions, typical for lead-like compounds with micromolar noncovalent affinity, are beneficial chemical starting points as they include recognition elements acting toward preserved binding mode and more straightforward optimization.

## Materials and Methods

### General Procedures and Instruments

For chemical synthesis, all starting materials and solvents were purchased from commercial vendors (Sigma-Aldrich, Fluorochem, and Combi-Blocks) and used without further purification. Reactions were monitored with Merck (Darmstadt, Germany) silica gel 60 F_254_ TLC plates. The column chromatography purifications were performed by using a Teledyne ISCO CombiFlash Lumen+ Rf. All tested compounds were >95% pure, as determined by HPLC analysis. ^1^H NMR spectra were recorded in DMSO-*d*_6_ or CDCl_3_ solution at room temperature on a Varian Unity Inova 500 spectrometer (500 MHz for ^1^H NMR spectra), with the deuterium signal of the solvent as the lock and TMS as the internal standard. Chemical shifts (δ) and coupling constants (*J*) are given in parts per million and Hz, respectively. HPLC-MS measurements were performed using a Shimadzu LCMS-2020 device equipped with a Reprospher 100 C18 (5 μm; 100 × 3 mm) column and a positive–negative double ion source (DUIS ±) with a quadrupole MS analyzer in a range of 50–1000 *m*/*z*. The sample was eluted with gradient elution using eluent A (10 mM ammonium formate in water:acetonitrile 19:1) and eluent B (10 mM ammonium formate in water:acetonitrile 1:4). The flow rate was set to 1 mL/min. The initial condition was 0% B eluent, followed by a linear gradient to 100% B eluent by 1 min; from 1 to 3.5 min, 100% B eluent was retained; and from 3.5 to 4.5 min, the system returned to the initial condition with 5% B eluent, which was retained until 5 min. The column temperature was kept at room temperature, and the injection volume was 10 μL. The purity of compounds was assessed by HPLC with UV detection; all tested compounds were >95% pure. High-resolution mass spectrometric measurements were performed using a Q-TOF Premier mass spectrometer (Milford, MA) in positive or negative electrospray ionization mode. Data acquisition and processing were performed using Analyst software version 1.6.2 (AB Sciex Instruments, CA, USA). Chromatographic separation was achieved by Purospher STAR RP-18 end-capped (50 mm × 2,1 mm, 3 μm) LiChrocart 55–2 HPLC cartridge. The sample was eluted with gradient elution using solvent A (0.1% formic acid in water) and solvent B (0.1% formic acid in acetonitrile). The flow rate was set to 0.5 mL/min. The initial condition was 5% B for 2 min, followed by a linear gradient to 95% B by 6 min; from 6 to 8 min, 95% B was retained; and from 8 to 8.5 min, the system returned to the initial condition with 5% B eluent, which was retained until 14.5 min. The column temperature was kept at room temperature, and the injection volume was 10 μL. Nitrogen was used as the nebulizer gas (GS1), heater gas (GS2), and curtain gas, with the optimum values set at 35, 45, and 45 (arbitrary units), respectively. The source temperature was 450 °C, and the ion spray voltage was set at 5000 V. The declustering potential value was set to 150 V.

### Thiol Reactivity Assay

For the GSH assay, a 500 μM solution of the fragment (PBS buffer, pH 7.4, 10% acetonitrile, 250 μL) with a 200 μM solution of indoprofen as the internal standard was added to a 10 mM l-glutathione solution (dissolved in PBS buffer, 250 μL) in a 1:1 ratio. The final concentrations were 250 μM fragment, 100 μM indoprofen, 5 mM l-glutathione, and 5% acetonitrile (500 μL). The final mixture was analyzed by HPLC-MS at 0, 1, 2, 4, 8, 12, 24, 48, and 72 h time intervals. In the case of fragments that were not detectable in a concentration of 250 μM, the final concentrations were reversed to 5 mM for the fragment and 250 μM for GSH. Degradation kinetics were also investigated using the previously described method, applying pure PBS buffer for the l-glutathione solution. In this experiment, the final concentrations of the mixture were 250 μM fragment, 100 μM indoprofen, and 5% acetonitrile. The AUC (area under the curve) values were determined via integration of HPLC spectra and corrected with the internal standard. The fragment AUC values were applied for ordinary least-squares (OLS) linear regression, and to compute important parameters (kinetic rate constant, half-life time), a programmed excel (Visual Basic for Applications) was utilized. The data are expressed as the means of duplicate determinations, with standard deviations within 10% of the given values.

### Radioligand Kinase Activity Assay for Bruton’s Tyrosine Kinase (BTK)

Covalent probes were tested with the ^33^PanQinase Activity Assay (by Reaction Biology Ltd.) on BTK. All kinase assays were performed in 96-well ScintiPlates from PerkinElmer (Boston, MA, USA) in a 50 μL reaction volume. All compounds were tested at 10 final assay concentrations, in singlicate. The final DMSO concentration in the reaction cocktails was 1% in all cases. The reaction cocktail was pipetted in four steps in the following order: 25 μL of assay buffer (standard buffer/[γ-^33^P]-ATP); 10 μL of ATP solution (in H_2_O); 5 μL of test compound (in 10% DMSO); 10 μL of enzyme/substrate mixture. The assay for the protein kinase contained 70 mM HEPES, pH 7.5, 3 mM MgCl_2_, 3 mM MnCl_2_, 3 μM Na-orthovanadate, 1.2 mM DTT, 50 μg/mL PEG_20000_, 3 μM ATP, and 5 μg/mL substrate. The reaction cocktails were incubated at 30 °C for 60 min. The reaction was stopped with 50 μL of 2% (v/v) H_3_PO_4_, and plates were aspirated and washed two times with 200 μL of 0.9% (w/v) NaCl. Incorporation of ^33^P_i_ was determined with a microplate scintillation counter (MicroBeta, Wallac). The residual activities for each concentration and the compound IC_50_ values were calculated using Quattro Workflow version 3.1.1 (Quattro Research GmbH, Munich, Germany). The fitting model for the IC_50_ determinations was “Sigmoidal response (variable slope)” with parameters “top” fixed at 100% and “bottom” at 0%. The fitting method used was a least-squares fit.

### Expression and Purification of Human BTK Kinase Domain

The expression and purification of the human BTK kinase domain (residues 387–659) were based on the method used by Bradshaw J. M. et al. The kinase domain was inserted into pFastBac-1 with an N-terminal 6×-His tag followed by a TEV protease cleavage site. (The plasmid was a gift from Dr. Ville Paavilainen, University of Helsinki.) Viruses were produced in Sf9 cells, and expression of the BTK kinase domain was subsequently induced in Tni insect cells by infecting 2 L of cultured cells with 1:200 mL virus solution such that cell growth was terminated after 3 days. The cells were collected by centrifugation (800 g for 15 min), and the pellet was resuspended in 50 mL of lysis buffer (10 mM Hepes, pH 7.5, 400 mM NaCl, 1.5 mM DTT) supplemented with 1× protease inhibitor cocktail (Roche). The cells were lysed by five passages through a cell homogenizer. The cellular debris were pelleted by centrifugation (30,000 × *g* for 30 min). The protein was bound in batch to nickel–nitrilotriacetic acid agarose beads in binding buffer (lysis buffer supplemented with 20 mM imidazole) for 4 h at 4 °C. The beads were washed with additional binding buffer (four 5 mL washes), and the protein was eluted with four 0.5 mL portions of elution buffer (lysis buffer S4 supplemented with 300 mM imidazole). The resulting soluble protein was purified further by gel filtration on a HiLoad 16/60 Superdex 75 (GE Healthcare) column equilibrated with 20 mM Tris, pH 8.0, 50 mM NaCl, and 1 mM DTT. The pure protein was then flash-frozen in liquid nitrogen and stored at −80 °C.

### MS-Occupancy Measurements for BTK

Covalent probes (IB and AC probes of Tables 1 and 2) were dissolved to a concentration of 10 mM in DMSO and stored at −80 °C. For conducting the experiment, a solution of 0.1 mM in DMSO from each molecule was prepared and diluted 2-fold in reaction buffer (HEPES 20 mM, pH 7.5, 50 mM NaCl) immediately prior to the experiment. 1 μL of this solution (50 μM) was mixed with 24 μL of 1 μM His_6_-BTK (giving 2 μM compound). The samples were incubated for 2 h at 25 °C, and the reaction was quenched by adding 25 μL of 0.8% formic acid. Two injections from each sample were performed. The LC/MS runs for His_6_-BTK were performed on a Waters ACQUITY UPLC Class H instrument in positive ion mode using electrospray ionization. UPLC separation used a C4-BEH column (300 Å, 1.7 μm, 21 mm × 100 mm). The column was held at 40 °C and the autosampler at 10 °C. Mobile phase A was 0.1% formic acid in water, and mobile phase B was 0.1% formic acid in acetonitrile. The run flow rate was 0.4 mL min^–1^. The gradient used was 1% B for 0.2 min, increasing linearly to 95% B for 1.6 min, holding at 85% B for 0.5 min, changing to 1% B in 0.2 min, and holding at 1% for 1 min. The MS data were collected on a Waters SQD2 detector with an *m*/*z* range of 2–3071.98 and a focus range of 600–1900 *m*/*z*. The desolvation temperature was 500 °C with a flow rate of 800 L h^–1^. The voltages used were 1.00 kV for the capillary and 24 V for the cone. Raw data were processed using OpenLynx and deconvoluted using MaxEnt with a range of 32500–36500 Da and a resolution of 1 Da/channel.

To estimate K_i_ and *k*_inact_ for the lead-size compounds, time-dependent BTK labeling experiments were performed in several concentrations of the compounds. To perform the labeling, 1 μL of 100X stocks of the compounds in DMSO was mixed with 99 μL of 1 μM His6-BTK in HEPES (25 mM, pH 7.5) and 50 mM NaCl. Incubation was performed at 25 °C. At defined time intervals, 10 μL samples were mixed with 30 μL of 20% acetonitrile +0.25% TFA, and 10 μL were injected into LCMS to quantify the labeling. Fitting was performed as described for the KRAS^G12C^ inhibitors.

### Expression and Purification of Human KRAS^G12C^

The cDNA sequence of KRAS-4B (UniProt ID: P01116-2) was ordered from GenScript, and the segment coding for residues 1–169 was cloned into a pET-45b vector following a His tag and a TEV protease site. The protein was expressed in *E. coli* BL21 Rosetta cells at 18 °C overnight and purified on a Ni-NTA column. TEV protease in a 1:10 molar ratio and 5 mM GDP were added during dialysis of the protein into 20 mM HEPES, 150 mM NaCl, 5 mM MgCl_2_, pH = 7.5. Residual His-tagged protein and the cleaved His tag were removed on a Ni-NTA column. This was followed by gel filtration in the dialysis buffer on a Superdex 200 10/300 GL column in an ÄKTA Purifier (GE Healthcare) system.

### KRAS^G12C^ MANT-GDP Exchange Assay

KRAS^G12C^ protein was first buffer-exchanged into low-magnesium buffer (20 mM HEPES pH 7.5, 50 mM NaCl, 0.5 mM MgCl_2_) using a NAP5 column (catalog no.: 17-0583-1, Cytiva). The proteins were then incubated with a 20-fold molar excess of *N*-methylanthraniloyl (MANT)-GDP (catalog no.: 69244, Sigma-Aldrich) in loading buffer (50 mM NaCl, 20 mM HEPES pH 7.5, 0.5 mM MgCl_2_, 10 mM EDTA, and 1 mM dithiothreitol (DTT)) in a total volume of 200 μL at 20 °C for 90 min. The reaction was stopped by adding MgCl_2_ to a final concentration of 10 mM and then incubated at 20 °C for 30 min. The unbound MANT-GDP was removed using the NAP5 column equilibrated with nucleotide exchange buffer (40 mM HEPES pH 7.5, 50 mM NaCl, 10 mM MgCl_2_, 2 mM dithiothreitol (DTT)) . Next, the MANT-GDP-bound KRas^G12C^ protein (in a final concentration of 1 μM) was incubated with the probes for 60 min. After the incubation, the MANT-GDP KRAS^G12C^-inhibitor mixtures were loaded into a black 384-well microplate in the presence or absence of the different inhibitor molecules at various concentrations. The nucleotide exchange reaction was initiated by adding a 100-fold molar excess of GppNHp (catalog no.: G0635, Sigma-Aldrich), a nonhydrolyzable GTP analog, and the SOS1 exchange domain (catalog no.: GE02, Cytoskeleton, Inc.) protein at a final concentration of 0.2 μM. The change in fluorescence intensity was measured every 30 s in RT for 60 min on an EnSpire plate reader (PerkinElmer, Inc.). The measured values were fitted to a single exponential function by using GraphPad Prism 10 software. The derived rates were normalized to the RAS-SOS1 minus RAS-only samples, from which the IC_50_ values were calculated from three independent experiments with each inhibitor using GraphPad Prism 10 software.

### MS-Occupancy Measurements for KRAS^G12C^

MS-based occupancy analysis of the protein was performed on a Sciex Triple Quad 3500 LC-MS/MS system (Sciex, Framingham, U.S.A.) equipped with a Turbo V ion source in electrospray ionization mode. Chromatographic separation was performed on a Sciex Exion 2.0 HPLC system consisting of a binary pump, an autosampler, and a column compartment. No real chromatographic separation was carried out; samples were only retained on a short security guard cartridge (Phenomenex wide-pore C4, 4 × 3 mm) to focus and desalt the protein samples prior to ionization, and the labeled and unlabeled proteins were not separated. A 4 min gradient (both in solvent composition and flow rate) was used with an initial flow of 0.5 mL/min and 10% eluent B. This was held for 0.5 min to wash out the salts from the sample. A 1.5 min linear increase was applied to reach a final flow of 1 mL/min and maximum eluent composition of B at 65%. These parameters were held for 0.5 min, and a quick 0.1 min linear gradient was used to reach the initial flow rate and eluent composition. This was followed by a 1.4 min equilibration phase . Water containing 0.1% formic acid (eluent A) and acetonitrile containing 0.1% formic acid were used for chromatography. The column temperature was kept at RT, and the injection volume was 10 μL. The MS parameters were as follows: 450 °C source temperature, 5000 V ion spray voltage, and 120 V declustering potential. Compressed air was used as the nebulizer gas (GS1) and heater gas (GS2), and nitrogen was used as the curtain gas, with values set at 35, 45, and 45, respectively. The protein was diluted to 100 μg/mL using a pH 7.4 PBS buffer. Twenty microliters of the diluted protein were treated with 0.2 μL DMSO solutions of the compounds at given concentrations. The incubation times used for the compounds varied between 20 s and 24 h. The reactions were quenched with 2 μL of 10% formic acid solution. Each experiment was measured in two biological replicates The ratio of the labeling was determined from the ratio of the MS peak heights. After identifying single occupancy levels in each experimental setup, these were plotted against time using GraphPad Prism Software, and based on one-phase experimental decay, we calculated the *k*_obs_ (s^–1^). These values were then plotted against the concentration of the probes, and *K*_i_ and *k*_inact_ were calculated directly from nonlinear regression according to the *k*_obs_ – c function as follows: 

### General Synthetic Procedures

#### General Procedure A

To a solution of the appropriate piperidine/pyrrolidine/piperazine intermediates in DCM, triethylamine (1 equiv) was added, and the mixture was allowed to stir under Ar at room temperature (RT) for 10 min. The mixture was cooled with iced water, and then the appropriate acyl chloride (1 equiv) was added dropwise, and the reaction was left to stir at RT for 2 h. The reaction mixture was concentrated under vacuum. The residue was diluted with H_2_O and extracted with EtOAc. The organic layer was washed with 1 M aq. HCl, dried over Na_2_SO_4_, filtered, and concentrated under vacuum. The product was then purified by preparative HPLC on a C18 column with an acetonitrile–water gradient elution.

#### General Procedure B

HATU (1-[Bis(dimethylamino)methylene]-1H-1,2,3-triazolo[4,5-*b*]pyridinium 3-oxide hexafluorophosphate, 1.2 equiv) was added to a solution of the appropriate acid (1.1 equiv), pyrrolidine intermediate (1 equiv), and DIPEA (2 equiv) in *N*,*N*-dimethylformamide (DMF). The reaction mixture was stirred at room temperature overnight. After 16 h, the mixture was extracted with EtOAc and brine. The organic layers were collected and dried over Na_2_SO_4_ and the solvent was removed by vacuum, and the crude product was purified by preparative HPLC on a C18-column with acetonitrile–water gradient elution.

#### Synthesis of the Covalent Probes

##### 1-(3-(4-Amino-1H-pyrazolo[3,4-*d*]pyrimidin-1-yl)piperidin-1-yl)prop-2-en-1-one (IB-L1)

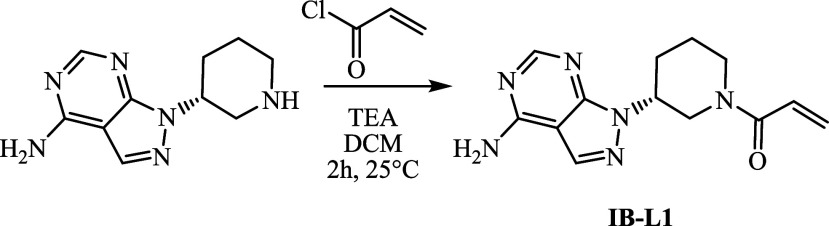



IB-L1 probe was synthesized following general procedure A, resulting in 42 mg (62%) product. ^1^H NMR (500 MHz, CD_3_OD) δ: 8.34 (s, 1H), 8.24 (s, 1H), 6.88–6.58 (m, 1H), 6.16 (t, *J* = 14.3 Hz, 1H), 5.70 (dd, *J* = 31.7, 10.9 Hz, 1H), 4.41–3.98 (m, 1H), 3.88–3.60 (m, 1H), 3.51–3.40 (m, 1H), 2.40–2.25 (m, 1H), 2.21 (s, 1H), 2.04 (s, 1H), 1.74–1.49 (m, 2H), 0.91–0.83 (m, 1H) ppm.

##### 1-(3-(4-Amino-1H-pyrazolo[3,4-*d*]pyrimidin-1-yl)piperidin-1-yl)-2-chloroethan-1-one **(IB-L2)**

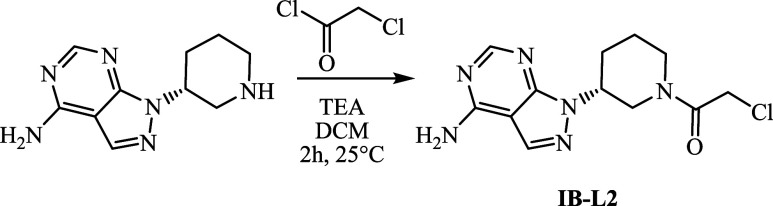



IB-L2 probe was synthesized following general procedure A, resulting in 38 mg (52%) product. ^1^H NMR (500 MHz, CD_3_OD) δ: 8.25 (s, 1H), 8.17 (s, 1H), 4.53 (d, *J* = 12.1 Hz, 1H), 4.32 (d, *J* = 11.3 Hz, 2H), 4.15 (s, 1H), 4.11–3.89 (m, 1H), 3.84–3.62 (m, 1H), 2.37–2.26 (m, 1H), 2.23–2.14 (m, 1H), 2.09–1.90 (m, 2H), 1.80–1.56 (m, 1H) ppm. ^13^C NMR (125 MHz, CDCl_3_) δ: 189.7, 158.7, 153.8, 149.3, 132.6, 100.8, 63.4, 49.5, 37.6, 33.0, 25.1, 14.5 ppm. HRMS (+TOF) C_12_H_16_N_6_OCl calculated mass 295.1074 and measured 295.1078.

##### 1-(3-(4-Amino-3-(4-phenoxyphenyl)-1H-Pyrazolo[3,4-*d*]pyrimidin-1-yl)piperidin-1-yl)-2-chloroethanone **(IB-D2)**

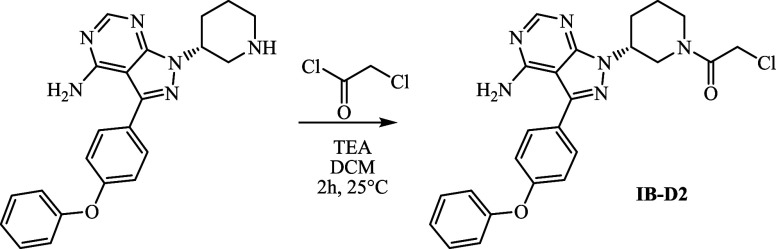



IB-D2 probe was synthesized following general procedure A, resulting in 155 mg (67%) product. ^1^H NMR (500 MHz, CDCl_3_) δ: 8.39 (d, *J* = 9.4 Hz, 1H), 7.66 (d, *J* = 8.4 Hz, 2H), 7.41 (t, *J* = 7.6 Hz, 2H), 7.19 (m, 3H), 7.11 (d, *J* = 7.9 Hz, 2H), 5.64 (s, 2H), 5.09–4.84 (m, 1H), 4.63 (dd, *J* = 147.9, 12.6 Hz, 1H), 4.18–4.10 (m, 2H), 4.00 (dd, *J* = 74.8, 13.3 Hz, 1H), 3.65 (dt, *J* = 201.4 Hz, 12.3 Hz, 1H), 3.12 (dt, J = 22.9, 11.8 Hz, 1H), 2.51–2.31 (m, 1H), 2.32–2.23 (m, 1H), 2.10–1.96 (m, 1H), 1.91–1.79 (m, 2H) ppm.

##### 1-(2-(8-Amino-1-bromoimidazo[1,5-*a*]pyrazin-3-yl)pyrrolidin-1-yl)but-2-yn-1-one **(AC-L1)**

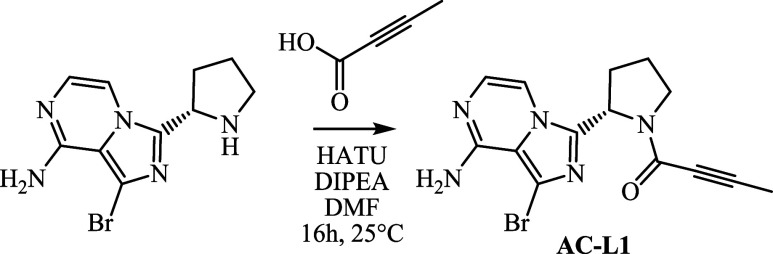



AC-L1 probe was synthesized following general procedure B, resulting in 20 mg (29%) product. ^1^H NMR (500 MHz, CD_3_OD) δ: 7.93 (d, *J* = 6.0 Hz, 1H), 6.98 (d, *J* = 5.9 Hz, 1H), 5.41–5.39 (m, 1H), 2.51–2.44 (m, 1H), 2.41–2.35 (m, 1H), 2.30–2.23 (m, 1H), 2.14–2.08 (m, 1H), 2.06 (s, 3H), 1.37–1.29 (m, 2H) ppm.

##### 1-(2-(8-Amino-1-bromoimidazo[1,5-*a*]pyrazin-3-yl)pyrrolidin-1-yl)-2-chloroethan-1-one **(AC-L2)**

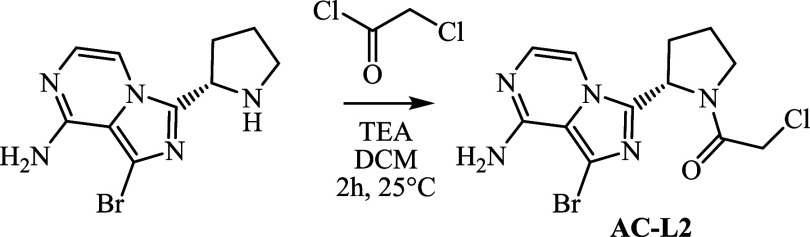



AC-L2 probe was synthesized following general procedure A, resulting in 41 mg (58%) product. ^1^H NMR (500 MHz, CD_3_CN) δ: 7.66 (d, *J* = 5.1 Hz, 1H), 7.06 (d, *J* = 5.1 Hz, 1H), 5.98 (broad s, 1H), 5.34 (dd, *J* = 7.9, 4.1 Hz, 1H), 4.19 (d, *J* = 6.2 Hz, 2H), 3.79–3.67 (m, 2H), 2.44–2.28 (m, 2H), 2.19–2.09 (m, 2H) ppm. ^13^C NMR (125 MHz, CDCl_3_) δ: 165.8, 149.5, 146.0, 132.2, 128.5, 115.8, 115.7, 112.5, 109.5, 52.6, 42.3, 30.9, 28.4, 25.4 ppm. HRMS (+TOF) C_12_H_14_N_5_OClBr calculated mass 358.0070 and measured 358.0077.

##### 4-(8-Amino-3-(1-(2-chloroacetyl)pyrrolidin-2-yl)imidazo[1,5-*a*]pyrazin-1-yl)-N-(pyridin-2-yl)benzamide **(AC-D2)**

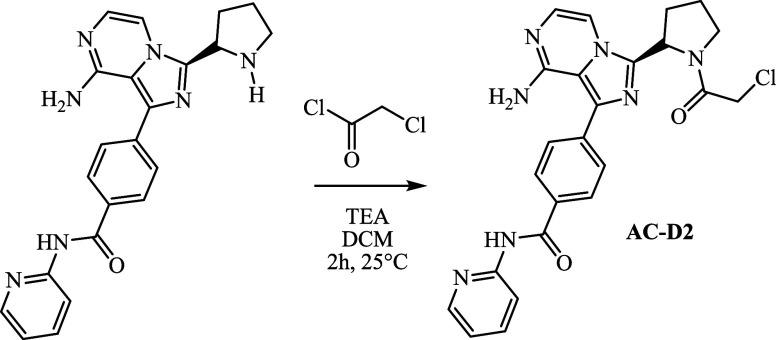



AC-D2 probe was synthesized following general procedure A, resulting in 22 mg (47%) product.^1^H NMR (500 MHz, CD_3_OD) δ: 8.39 (s, 1H), 8.27 (d, *J* = 8.3 Hz, 1H), 8.15 (d, *J* = 8.3 Hz, 2H), 7.87 (t, *J* = 7.9 Hz, 1H), 7.82 (d, *J* = 8.2 Hz, 2H), 7.75 (d, *J* = 4.8 Hz, 1H), 7.25–7.16 (m, 1H), 7.13 (s, 1H), 5.55–5.52 (m, 1H), 4.35 (d, *J* = 13.7 Hz, 1H), 4.25 (d, *J* = 13.7 Hz, 1H), 3.96–3.92 (m, 1H), 3.83–3.78 (m, 1H), 2.47–2.43 (m, 1H), 2.40–2.34 (m, 1H), 2.24–2.12 (m, 2H) ppm. ^13^C NMR (125 MHz, CD_3_OD) δ: 182.9, 172.9, 147.8, 145.3, 141.6, 138.2, 137.6, 134.0, 130.0, 127.8, 126.8, 120.0, 116.3, 114.9, 110.0, 53.3, 41.9, 30.5, 24.5 ppm. HRMS (+TOF) C_24_H_23_N_7_O_2_Cl calculated mass 476.1601 and measured 476.1601.

##### *Tert*-butyl 3-(cyanomethyl)-4-(2-fluoroacryloyl)piperazine-1-carboxylate **(AD-F1)**

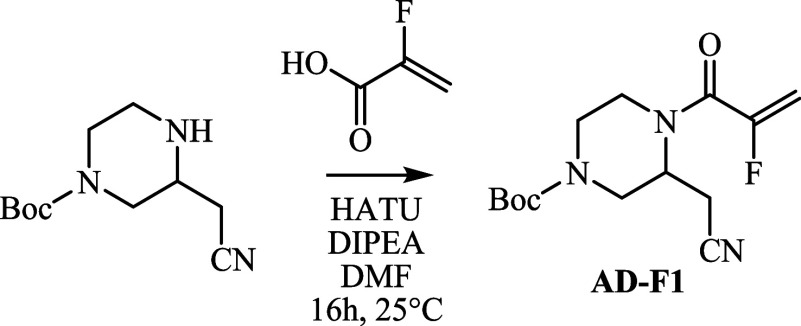



AD-F1 probe was synthesized following general procedure B and resulted in 55 mg (37%) product. ^1^H NMR (500 MHz, CDCl_3_) δ: 5.38 (d, *J* = 46.3 Hz, 1H), 5.23 (dd, *J* = 16.9, 3.6 Hz, 1H), 4.20–4.10 (m, 3H), 3.18–3.13 (m, 2H), 2.93 (s, 1H), 2.80–2.75 (m, 1H), 2.69 (s, 1H), 1.69 (s, 1H), 1.51 (s, 9H) ppm. ^13^C NMR (125 MHz, CDCl_3_) δ: 194.4, 176.0, 157.7, 116.3, 100.7, 57.4, 47.6, 37.6, 36.3, 31.6, 28.3 ppm. HRMS (+TOF) C_14_H_21_N_3_O_3_F calculated mass 298.1566 and measured 298.1568.

##### *Tert*-butyl 4-(2-chloroacetyl)-3-(cyanomethyl)piperazine-1-carboxylate **(AD-F2)**

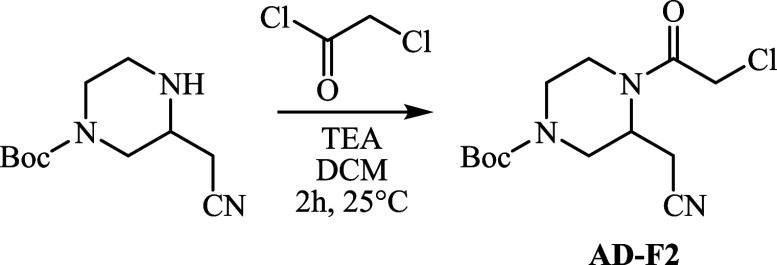



AD-F2 probe was synthesized following general procedure B, resulting in 86 mg (57%) product. ^1^H NMR (500 MHz, CDCl_3_) δ: 4.88 (s, 1H), 4.13–4.07 (m, 3H), 3.73 (d, *J* = 13.3 Hz, 1H), 3.40 (s, 1H), 3.18 (d, *J* = 13.2 Hz, 1H), 3.01 (s, 1H), 2.82–2.61 (m, 3H), 1.51 (s, 9H) ppm. ^13^C NMR (125 MHz, CDCl_3_) δ: 209.4, 175.9, 116.4, 46.2, 41.7, 41.6, 40.7, 36.7, 28.3, 11.2 ppm. HRMS (+TOF) C_13_H_21_N_3_O_3_Cl calculated mass 302.1271 and measured 302.1273.

##### *Tert*-butyl 4-(3-(cyanomethyl)-4-(2-fluoroacryloyl)piperazin-1-yl)-5,8-dihydropyrido[3,4-*d*]pyrimidine-7(6H)-carboxylate **(AD-L1)**

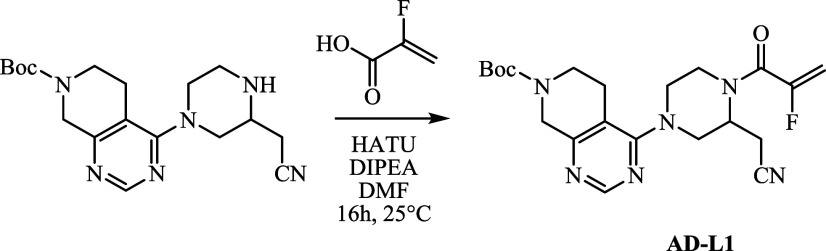



*Tert*-butyl (S)-4-(3-(cyanomethyl)piperazin-1-yl)-5,8-dihydropyrido[3,4-*d*]pyrimidine-7(6H)-carboxylate (24 mg, 0.07 mmol), triethylamine (0.034 mL; 0.21 mmol), and HATU (30 mg, 0.084 mmol) were dissolved in 1 mL of acetonitrile. 2-Fluoroprop-2-enoic acid (6.5 mg, 0.08 mmol) was added to the mixture and stirred at room temperature for 30 min. The reaction mixture was evaporated and purified with preparative chromatography (eluent: water:CH_3_CN containing 0.1% formic acid on a C8 column). We obtained 20 mg (71%) white powder. ^1^H NMR (500 MHz, CD_3_OD-d4) δ: 8.52 (s, 1H), 5.39–5.26 (m, 2H), 4.92 (s, 1H), 4.61 (d, *J* = 18.7 Hz, 1H), 4.48 (d, *J* = 18.7 Hz, 1H), 4.14 (dt, *J* = 13.9, 2.2 Hz, 1H), 4.06–3.98 (m, 1H), 3.77 (dt, *J* = 13.1, 5.2 Hz, 1H), 3.51 (s, 2H), 3.31 (d, *J* = 3.8 Hz, 2H), 3.12 (d, *J* = 14.1 Hz, 2H), 2.99 (dd, *J* = 17.1, 6.7 Hz, 1H), 2.82 (t, *J* = 5.6 Hz, 2H), 1.52 (s, 9H). ^13^C NMR (126 MHz, CD_3_OD) δ: 164.87, 162.30, 162.05, 160.97, 157.48, 155.35, 154.70, 117.06, 116.34, 99.50, 80.49, 48.59, 48.11, 48.06, 47.95, 47.89, 47.77, 47.70, 47.60, 47.43, 47.38, 47.26, 47.09, 27.25, 25.79. HRMS (+TOF) C_21_H_28_N_6_O_3_F calculated mass 431.2201 and measured 431.2190

##### *Tert*-butyl 4-(4-(2-chloroacetyl)-3-(cyanomethyl)piperazin-1-Yl)-5,8-dihydropyrido[3,4-*d*]pyrimidine-7(6H)-carboxylate **(AD-L2)**

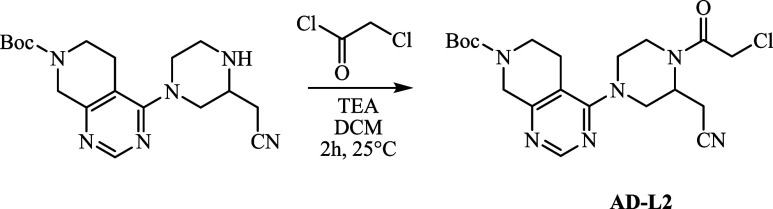



*Tert*-butyl (S)-4-(3-(cyanomethyl)piperazin-1-yl)-5,8-dihydropyrido[3,4-*d*]pyrimidine-7(6H)-carboxylate (30 mg, 0.10 mmol) and triethylamine (0.036 mL; 0.30 mmol) were dissolved in 1 mL of dichloromethane and cooled to 0 °C. Chloroacetyl chloride (0.014 mL, 0.20 mmol) was added to the solution at 0 °C. The mixture was stirred at room temperature for 1 h. The reaction mixture was evaporated and purified with preparative chromatography (eluent: water:CH_3_CN containing 0.1% formic acid on C8 column). We obtained 10 mg (29%) of white powder. ^1^H NMR (500 MHz, CD_3_OD) δ: 8.50 (s, 1H), 4.97 (s, 1H), 4.60 (d, *J* = 17.6 Hz, 2H), 4.47 (s, 1H), 4.34 (d, *J* = 12.0 Hz, 2H), 4.10 (d, *J* = 12.8 Hz, 2H), 3.99 (s, 1H), 3.76 (s, 1H), 3.47 (s, 2H), 3.31 (tt, *J* = 5.4, 2.6 Hz, 6H), 2.95 (d, *J* = 30.1 Hz, 3H), 2.80 (s, 3H), 1.50 (s, 9H). ^13^C NMR (126 MHz, CD_3_OD) δ: 168.45, 166.11, 162.36, 156.1, 117.6, 81.89, 50.02, 48.87, 48.79, 47.11, 41.81, 41.45, 28.65, 27.29, 18.8. HRMS (+TOF) HRMS (+TOF) C_20_H_28_N_6_O_3_Cl calculated mass 435.1905 and measured 435.1891

##### 2-(1-(2-Chloroacetyl)-4-(7-(8-chloronaphthalen-1-yl)-2-((1-methylpyrrolidin-2-yl)methoxy)-5,6,7,8-tetrahydropyrido[3,4-*d*]pyrimidin-4-yl)piperazin-2-yl)acetonitrile **(AD-D2)**

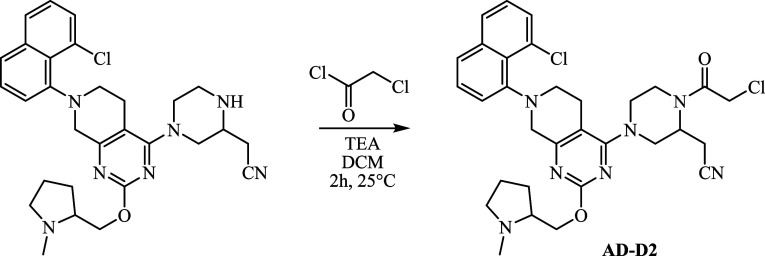



AD-D2 probe was synthesized following general procedure A, resulting in 13 mg (28%) product. ^1^H NMR (500 MHz, CD_3_OD) δ: 7.82 (d, *J* = 8.1 Hz, 1H), 7.68 (d, *J* = 8.1 Hz, 1H), 7.53–7.46 (m, 2H), 7.39–7.29 (m, 2H), 4.72–4.67 (m, 1H), 4.60–4.50 (m, 2H), 4.40–4.11 (m, 4H), 3.81–3.55 (m, 6H), 3.31 (s, 3H), 3.23–3.13 (m, 4H), 3.02–3.00 (m, 3H), 2.79–2.77 (m, 1H), 2.18–1.99 (m, 3H) ppm.

#### Synthesis of the Lead-Level Intermediers

##### 1-Bromo-3-(pyrrolidin-2-yl)imidazo[1,5-*a*]pyrazin-8-amine **(AC-Int)**

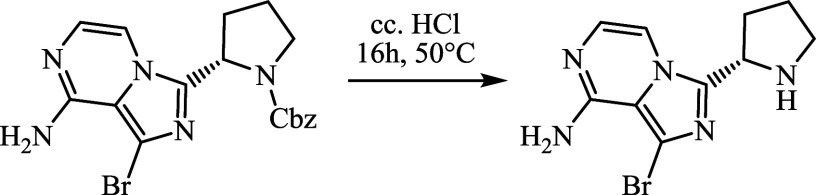



The Cbz-protected pyrrolidine derivative (1 mmol, 415 mg) was dissolved in aqueous cc. HCl and stirred overnight at 50 °C. After 16 h, the reaction mixture was quenched with sat. NaHCO_3_ aqueous solution until a basic pH was reached and then the product was extracted with EtOAc several times. The organic layers were collected, dried over Na_2_SO_4_, and concentrated. The crude product was washed with cold water and purified by flash chromatography on a reversed phase C18 column with acetonitrile–water gradient elution to give 136 mg (48%) product. ^1^H NMR (500 MHz, CDCl_3_) δ: 7.63 (d, *J* = 5.1 Hz, 1H), 7.00 (d, *J* = 5.2 Hz, 1H), 3.63–3.59 (m, 1H), 3.12–2.98 (m, 3H), 2.30–2.15 (m, 3H), 2.04–2.03 (m, 1H) ppm.

##### *Tert*-butyl 4-(3-(cyanomethyl)piperazin-1-yl)-5,8-dihydropyrido[3,4-*d*]pyrimidine-7(6H)-carboxylate **(AD-Int)**

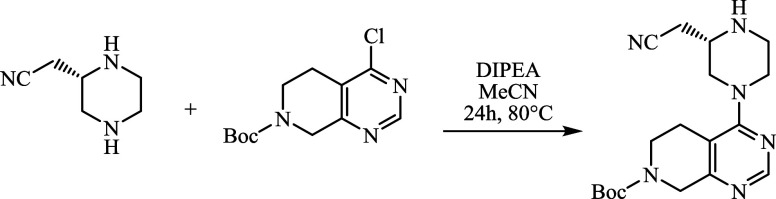



*Tert*-butyl 4-chloro-5H,6H,7H,8H-pyrido[3,4-*d*]pyrimidine-7-carboxylate (100 mg; 0.37 mmol) was dissolved in acetonitrile (2 mL). 2-[(2S)-piperazin-2-yl]acetonitrile dihydrochloride (73 mg; 0.37 mmol) and DIPEA (0.260 mL; 1.5 mmol) were added to the solution. The mixture was stirred at 80 °C for 2 h. The reaction mixture was evaporated and purified with flash chromatography (eluent: water:CH_3_CN containing 0.1% formic acid on C8 column). We obtained 60 mg (45%) white powder. ^1^*H* NMR (500 MHz, CD_3_OD-d4) δ: 8.48 (s, 1H), 4.52 (s, 2H), 4.06–3.99 (m, 1H), 3.85 (d, *J* = 12.6 Hz, 1H), 3.61 (s, 2H), 3.33 (dt, *J* = 3.3, 1.7 Hz, 1H), 3.17 (dtd, *J* = 9.4, 6.3, 2.9 Hz, 1H), 3.09 (ddd, *J* = 13.8, 11.8, 3.0 Hz, 2H), 2.98–2.85 (m, 2H), 2.75 (t, *J* = 5.6 Hz, 2H), 2.72–2.61 (m, 2H), 1.52 (d, *J* = 1.2 Hz, 9H). ^13^*C* NMR (126 MHz, CD_3_OD) δ: 164.57, 154.74, 154.62, 117.27, 116.04, 80.48, 51.74, 50.89, 44.49, 27.26, 26.07, 20.58. HRMS (+TOF) C_18_H_27_N_6_O_2_ calculated mass 359.2190 and measured 359.2178.

## Supporting Information Available

Mathematical formulas for occupancy, and experimental details (PDF)
Mathematical formulas for occupancy, and experimental details (CSV)
