# Supplementary material for: Size-Dependent Target Engagement of Covalent Probes
Source: J Med Chem. 2025 Mar 18;68(6):6616–32. doi: 10.1021/acs.jmedchem.5c00017 (PMC11956015; doi:10.1021/acs.jmedchem.5c00017)
Supplement: Supplementary file 1 — jm5c00017_si_001.pdf [file jm5c00017_si_001.pdf]

SUPPORTING INFORMATION

Size dependent target engagement of covalent probes

László Petri[a†], Ronen Gabizon[b†], György G. Ferenczy[a†], Nikolett Péczka[a,c], Attila Egyed[a], Péter Ábrányi-Balogh[a], Tamás Takács[d,e], György M. Keserű[a,c*]

[a]HUN-REN Research Centre for Natural Sciences, Medicinal Chemistry Research Group and National Drug Discovery and Development Laboratory, 2 Magyar tudósok krt, Budapest, 1117, Hungary

[b]Department of Chemical and Structural Biology, Weizmann Institute of Science, Helen and Milton A. Kimmelman bldg, Rehovot 76100, Israel

[c]Budapest University of Technology and Economics, Department of Organic Chemistry and Technology, 8 Budafoki út, Budapest, 1111, Hungary

[d]HUN-REN Research Centre for Natural Sciences, Signal Transduction and Functional Genomics Research Group, 2 Magyar tudósok krt., Budapest, 1117, Hungary

[e]Doctoral School of Biology, Institute of Biology, ELTE Eötvös Loránd University, Pázmány Péter sétány 1/A, Budapest, 1117, Hungary

[†]These authors contributed equally

*corresponding author's e-mail address: keseru.gyorgy@ttk.hu

Contents of Supporting Information

1. Formulas describing covalent labeling of proteins 2
2. Labeling increases with reactivity for top labeling fragment-sized compounds 5
3. Labeling increases with reactivity for fragment-sized compounds 6
4. Labeling increases with reactivity for minifragments 7
5. Labeling increases with increasing reactivity in proteomic studies 9
6. Three-step model of covalent labeling 12
7. Variation of the inactivation rate constant in the protein environment 14
8. Parameter derivation for the pH dependent labeling of $KRAS^{G12C}$ by ARS-853 15
9. Linear relationship between IC_{50} and K_I/k_{inact} for $KRAS^{G12C}$ inhibitors 16
10. MS occupancy results 17
11. k_{inact}/K_I determination 20
12. HPLC-MS purity of the synthesized covalent probes 22
13. ^{1}H-NMR spectrum of the synthesized covalent probes 27
14. References 34

1. Formulas describing covalent labeling of proteins

Here we provide formulas generally describing covalent labeling of nucleophilic protein residues. In the following discussion we consider covalent enzyme inhibition where the covalent agent inhibits the transformation of the substrate by the enzyme (both steps in Scheme 1) and also the displacement of a ligand typically bound to a receptor (first step in Scheme 1).

$$\mathrm{P} + \mathrm{S} \underset{k_{\mathrm{off}}}{\overset{k_{\mathrm{on}}}{\rightleftharpoons}} \mathrm{P{\bullet}S} \xrightarrow{k_{cat}} \mathrm{P} + S'$$

Scheme 1 Covalent labeling inhibiting two alternative processes is investigated; 1) enzymatic catalysis where the substrate (S) first reversibly binds to the enzyme (P) and the substrate is converted in the second step and 2) ligand receptor binding where only the first reversible step occurs.

The formulas below contain $[S]$ and $K_M = \frac{k_{off}+k_{cat}}{k_{on}}$ that are the substrate concentration and Michaelis constant, respectively, for the enzymatic reaction. For ligand-receptor binding, $[S]$ is the concentration of the ligand displaced by the covalent agent and $K_M = \frac{k_{off}}{k_{on}}$ is the dissociation constant of this ligand. It is assumed that the ligand concentration [L] is much higher than the protein concentration $[P_T]$, and if a substrate is present then its concentration [S] is also much higher than $[P_T]$.

According to the two-step process discussed above, the target occupancy, the observed reactivity, the percentage of inhibition and the IC_{50} values can be related to the parameters k_{inact} and K_I.

$$\textit{Occupancy(t)} = \frac{[PL]}{[P_T]} = 1 - \exp\left(-k_{obs}t\right) \tag{1}$$

where the observed first order rate of inactivation is

$$k_{obs} = k_{inact}\frac{[L]}{K_I\left(1+\frac{[S]}{K_M}\right)+[L]} \approx \frac{k_{inact}[L]}{K_I\left(1+\frac{[S]}{K_M}\right)} \tag{2}$$

When the ligand concentration, $[L]$, is small compared to the inhibition constant K_I, then the approximate formula $k_{obs} \approx \frac{k_{inact}[L]}{K_I\left(1+\frac{[S]}{K_M}\right)}$ is sometimes used. When covalent labeling occurs in the absence of substrate, or competing ligand, then the $\frac{[S]}{K_M}$ term is missing. The approximate formula contains k_{inact}/K_I, that has a form of a second order rate constant and characterizes the rate of covalent modification for small ligand concentrations[1].

Percentage inhibition is expressed as

$$I\%(t) = 100 * \left(1 - \frac{1 + \frac{[S]}{K_M}}{1 + \frac{[L]}{K_I} + \frac{[S]}{K_M}} exp(-k_{obs} \cdot t) \right) \quad (3)$$

and this agrees with the occupancy formula (Eq. (1)) when the ligand concentration is small compared to the inhibition constant ($\frac{[L]}{K_I} \approx 0$). Eq. (3) is valid for enzyme inhibition when it is measured in terms of reaction velocity: $I\% = 100 * \left(1 - \frac{v_L}{v_0}\right)$ with v_L and v_0 being the reaction velocity with and without the covalent agent. In the case of ligand-receptor binding, Eq. (3) gives the percentage of ligand displaced by the covalent agent.

The $IC_{50}(t)$ defined as the inhibitor concentration that reduces the enzyme activity (reaction velocity) to half of its value without inhibitor is expressed as [2]

$$IC_{50}(t) = K_I\left(1 + \frac{[S]}{K_M}\right) \cdot \left(2 \cdot exp\left(-k_{obs,IC_{50}} \cdot t\right) - 1\right) \quad (4)$$

This formula also applies for ligand-receptor binding and expresses the concentration of covalent ligand that displaces half of the reference ligand, *S*. It is to be noted that $Occupancy$, $I\%$ and and IC_{50} are time-dependent, and the latter decreases with increasing time. When IC_{50} is measured with a long incubation time for a compound with sufficiently high k_{inact} then the $IC_{50}(t)$ is much smaller than the initial $IC_{50}(t = 0)$ and the approximate relationship between $IC_{50}(t)$ and $\frac{K_I}{k_{inact}}$ can be written as [2,3]

$$IC_{50}(t) \approx \frac{ln(2)}{t}\left(1 + \frac{[S]}{K_M}\right)\frac{K_I}{k_{inact}} \quad (5)$$

showing a linear relationship between $IC_{50}(t)$ and $\frac{K_I}{k_{inact}}$.

When enzyme inhibition is characterized by end-point measurements (product formation) the percentage inhibition is written as

$$I\%(P;t) = 100 * \left(1 - \frac{1 + \frac{[S]}{K_M}}{1 + \frac{[L]}{K_I} + \frac{[S]}{K_M}} \frac{1 - exp(-k_{obs} \cdot t)}{k_{obs}} \right) \quad (6)$$

where $I\%(P;t) = 100 * \left(1 - \frac{[P]_L}{[P]_0}\right)$, with $[P]_L$ and $[P]_0$ being the product concentration in the presence and absence of the inhibiting ligand. The corresponding $IC_{50}(P;t)$ is expressed as [4]

$$IC_{50}(P;t) = K_I\left(1 + \frac{[S]}{K_M}\right) \cdot \left(\frac{2 - 2 \cdot exp\left(-k_{obs,IC_{50}} \cdot t\right)}{k_{obs,IC_{50}} \cdot t} - 1\right) \quad (7)$$

and with the assumption that $IC_{50}(P;t) \ll IC_{50}(P;0)$ the above equation reduces to

$$IC_{50}(P;t) \approx \frac{1.59}{t}\left(1 + \frac{[S]}{K_M}\right)\frac{K_I}{k_{inact}} \quad (8)$$

Finally, we note that in endpoint enzyme inhibition measurements the determination of k_{inact} and K_I with numerical fitting was proposed when the enzyme is incubated with inhibitor prior to the addition of substrate that leads to biphasic time-dependent equations [5].

2. Labeling increases with reactivity for top labeling fragment-sized compounds

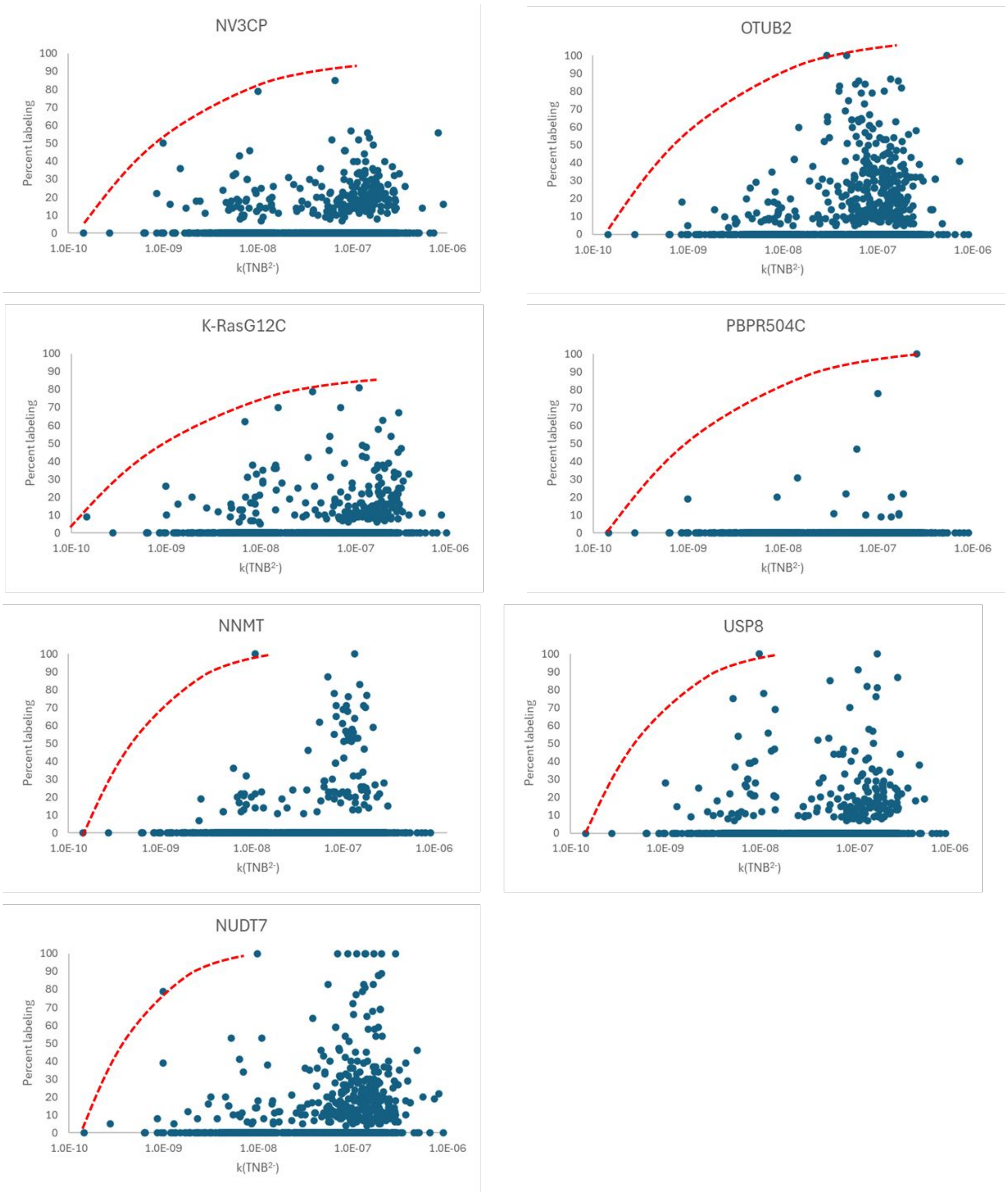

Figure S1 Percent labeling versus reactivity (k_{TNB2-}) against TNB^{2-}. Data are shown for targets (seven out of ten) where high labeling was reported in ref [6]. Labeling tends to increase for top labeling compounds as indicated by the red envelopes.

3. Labeling increases with reactivity for fragment-sized compounds

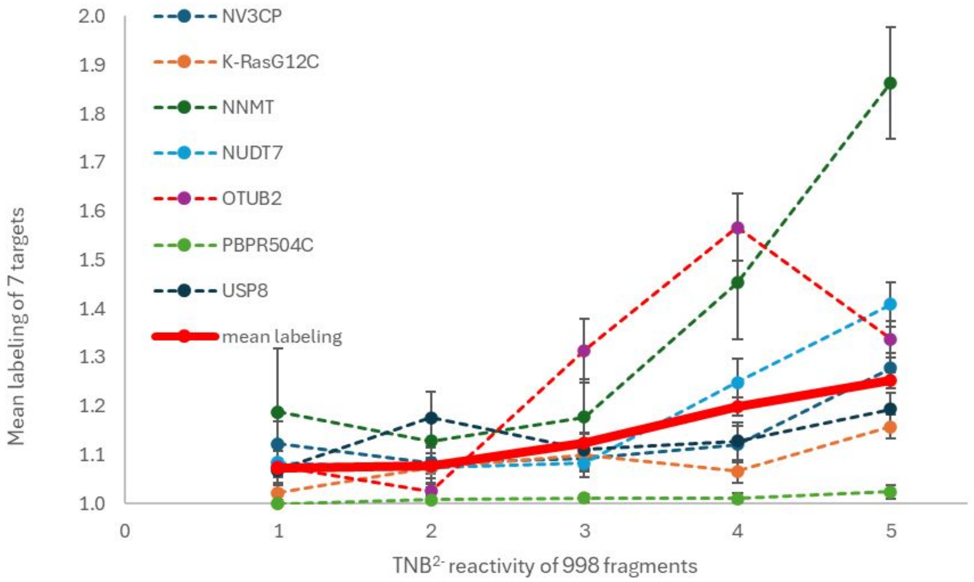

Figure S2 Labeling of targets averaged for compounds with the same TNB^{2-} reactivity. Labeling and reactivity data from ref [6]. Labeling is binned into classes 1-4; higher number represents higher labeling. TNB^{2-} reactivity is binned into classes 1-5; higher number represents higher reactivity. Mean labeling of the 7 targets (thin red line) by fragment reactivity is also shown. Standard errors are indicated by vertical bars.

4. Labeling increases with reactivity for minifragments

Table S1 GSH reactivity and biochemical activity of minifragments

compound[a]	GSH $t_{1/2}$ (h)[a]	HDAC8 RA%[a]	$MurA_{EC}$ RA%[b]	Target A RA%	Target B RA%	Target C RA%	Target D RA%	Target E RA%
**A1+**	0.06	105.85	21	97.6	61	43	88.4	100
**A2+**	0.06	2.38	30	3.9	77	27.4	72.7	98.1
**A3+**	0.07	1.35	23.3	0.2	25.5	19.4	54.2	100
**A4+**	>48	1.02	19.5	21.1	87.3		95.6	97.1
**A5+**	>48	1.7	24.7	46.1	82.1	91.8	88.3	100
**A6+**	7.70	0.67	24.2	0	0	80.2	36.1	81.1
**B1+**	13.10	1.54	89.9	37.8	87.2		89.5	97.7
**B2+**	19.10	0.8	65.8	34.9	98.8		86.9	92.4
**B3+**	>48	1.5	77	10.3	87.1		82.2	100
**B4+**	>48	1.09	100	56.8	81.6		97.6	95.6
**B5+**	>48	1.88	80.8	34.4	85.7		74.8	100
**B6+**	>48	0.58	74.3	41.7	92.5		66.6	100
**C1+**	>48	3.74	31.1	9.9	100		60.1	95.6
**C2+**	>48	0.17	39.4	4.2	99	81.2	60.9	87.4
**C3+**	0.40	0	-1.5	0	9.5	76.8	12.9	21.7
**C4+**	>48	0.82	61.7	45.8	97.6		93.7	97.5
**C5+**	11.10	0.34	37.5	14.4	97.8		78.3	100
**C6+**	0.08	0	24.9	0	28.6	43.4	60.5	89.3
**D1+**	*0.00*	1.16	99.1	80.1	95		58.5	61.5
**D2+**	*0.00*	1.61	98.2	100	100		50	75.6
**D3+**	>48	1.08	8.6	-0.2	0	77.7	45.5	68.8
**D6+**	*0.00*	0	6.7	0	5.3	102	13.9	8.4
**E1+**	*0.00*	44.9						
**E4+**	*3.10*	1.12	83.6	80.7	84.9	97	95.1	79.9
**F1+**	>48	1.67	97.9	95.9	100		96.8	77.9
**F2+**	>48	1.78	95.5	54.3	95.1		95	84.8
**F3+**	>48	1.95	4.2	63.9	100		78.7	59.1
**F4+**	*0.00*		24.3	-0.7	-1	97.8	33.5	81.2
**F5+**	22.40	0.55	75.8	59.4	40.1		77.8	89.2
**F6+**	*0.00*	2.66	77.8	35.4	55.6		71.4	71.6
**G1+**	0.07	0.68	63.9	17.3	89.7	63.9	100	94.3
**G2+**	0.00	0.41	40.1	57.4	79	24.6	96.7	94.3
**G3+**	0.00	0	5.5	0	0.6	97.3	19.9	63.4
**G4+**	>48	58.92	70.1	54.3	90.9		84.2	82.3
**G5+**	>48	4.22	38.3	6.5	100		95.1	100
**G6+**	0.00	0	100	4.7	61.4		24.7	52.3
**H1+**	*34.40*	1.3	100	50	88.2		92.1	88.9
**H2+**	3.60	1.13	82.9	71.3	86.1		92.4	
**H3+**	0.00	0.91	10.4	52.7	80.2	97.8	72.7	
**H4+**	>48	1.01	100	84	79.4		60.9	
**J1+**	>48	27.85	100	48	95.8		88.4	
**J2+**	>48	1.21	65.5	28.7	79.9		62.7	
**J3+**	>48	1.21	98.9	9.6	64.8		86.9	
**J4+**	>48	1.3	96.1	69	83.4		54.2	
**K2+**	>48	1.76	97.5	80.4	87.4		95.6	
**K3+**	47.60	1.61	99.7	25.6	72.5		93.7	
**K5+**	>48	2.09	100	45.8	64.6		74.9	
**K6+**	19.40	2.54	100					
**L1+**	>48	3.85	99.7	93.5	88.1		82.2	
**L2+**	>48	1.58	97.1	69.4	70.6		13	
**L3+**	>48	1.1	100	87.4	85.2		97.6	
**L4+**	>48	107.15	99.1	59.7	83.9		88.3	
**L6+**	>48	1.11	91.2	42.6	76.6		78.3	
**N3+**	0.00	0.82	1.2	47.6	74	87.3	36.1	
**P3+**	>48	1.65	22.2	61.3	79		66.6	
**Q2+**	*1.80*	0	10.3	32.8	67.8		55.5	80.5
**R2+**	0.00	4.56	42.8	2.5	0		60.5	
**R4+**	*22.60*	88.5	57.2	30.5	68.4		66.7	100

[a]ref. [7] [b]ref. [8]

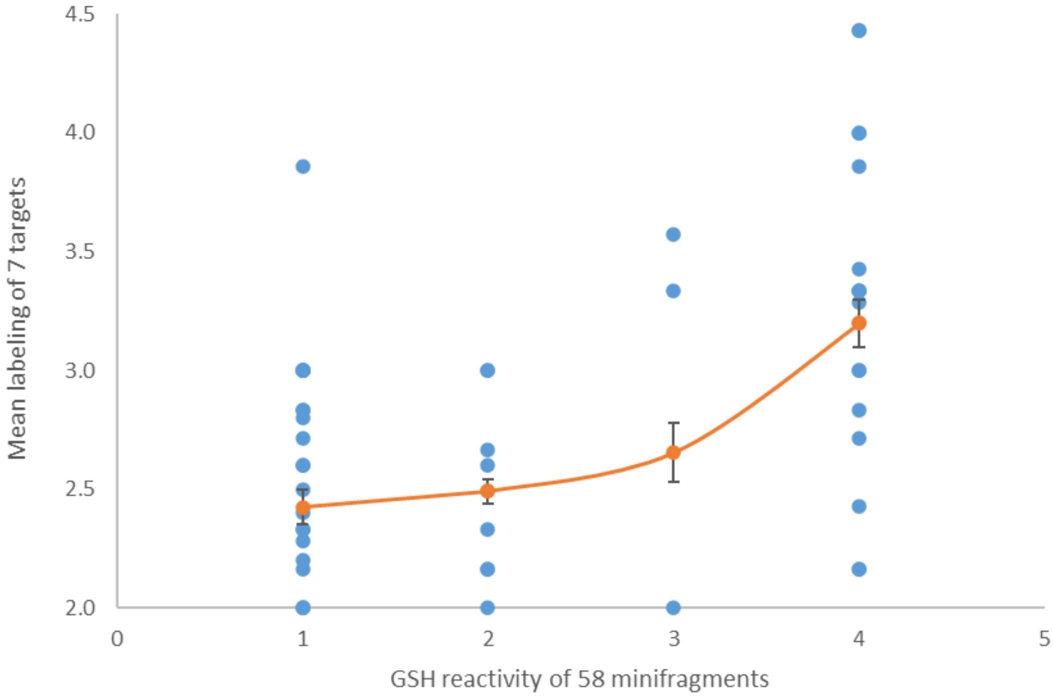

Figure S3 Mean labeling of 7 targets versus GSH reactivity for 58 minifragments (Table S1). Labeling is binned into classes 1-5; higher number represents higher labeling. GSH reactivity is binned into classes 1-4; higher number represents higher reactivity. Mean(orange) labeling of minifragments also shown; standard error is indicated by vertical bars.

5. Labeling increases with increasing reactivity in proteomic studies

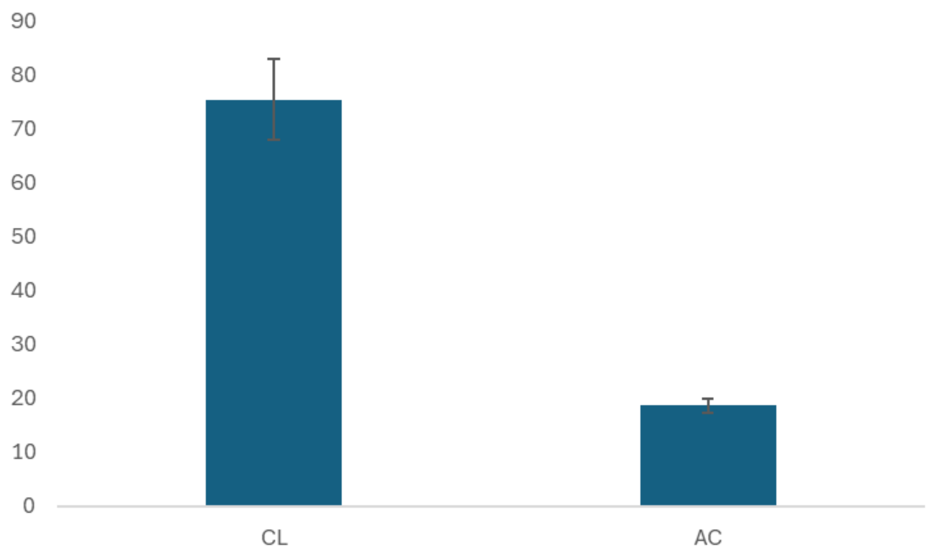

Figure S4. Average number of labelled amino acids by compounds with chloroacetamide (CL) and acrylamide (AC) warheads as reported in ref. [9]. The standard error of mean is also indicated.

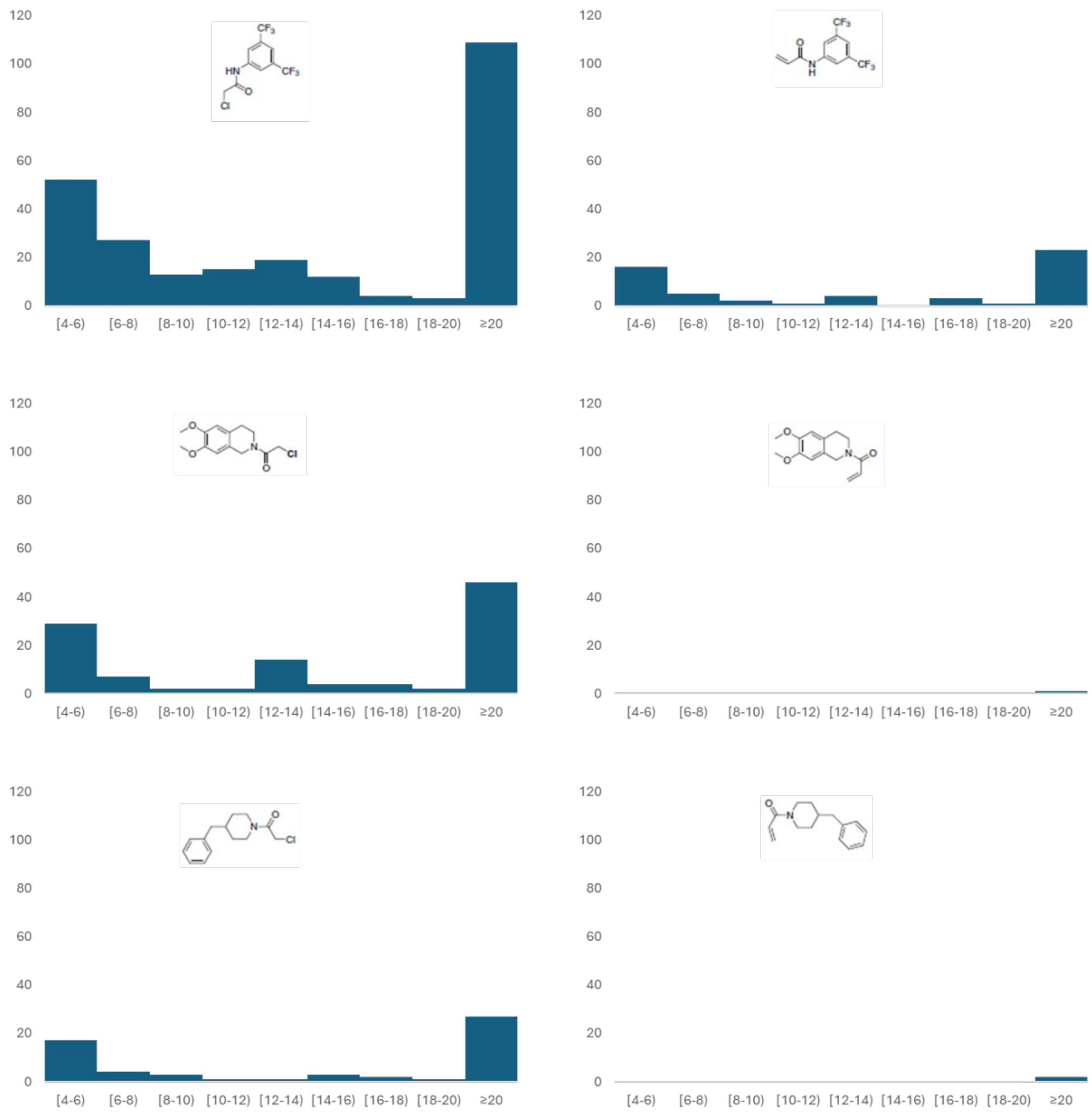

Figure S5. Labeling efficiency distribution of compound pairs with the same skeleton and with either chloroacetamide (left) or acrylamide (right) warhead. Labeling efficiency increases left to right. Data are from ref. 10. In accordance with ref. 10 compounds with R<4 are considered as non-labeling and are not shown.

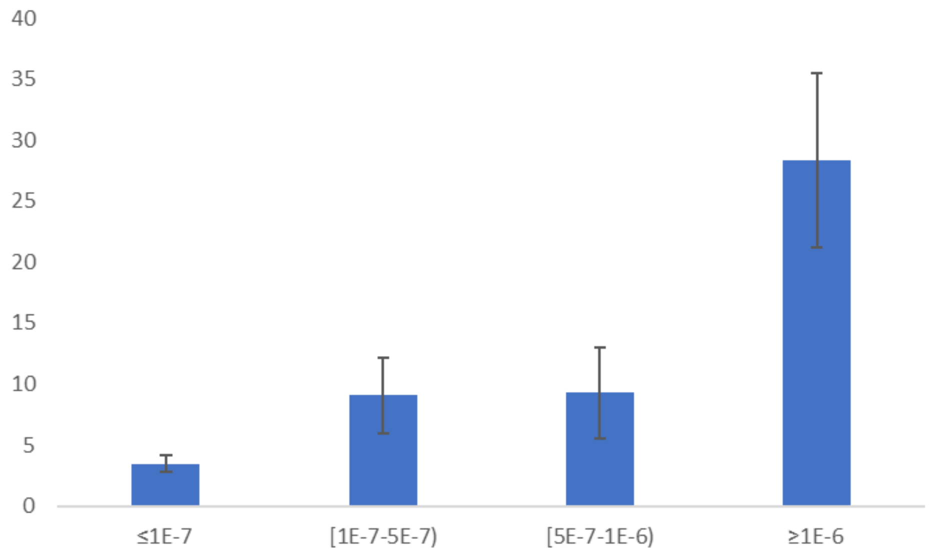

Figure S6. Average number of liganded lysines versus model reactivity based on data in ref. 11. Standard error of mean is also indicated. Reactivities (k) are calculated from the reported product amount ([P]) with the formula $k = \frac{ln\left(1-\frac{[P]}{[A_0]}\right)}{t}$ with $[A_0] = 125\mu M$ is the initial concentration of the aminophilic compound and $t = 3600s$ is the incubation time. The reactivities were binned as indicated on the horizontal axes.

6. Three-step model of covalent labeling

Here, we present how kinetic parameters of a three-step covalent labeling reaction (Scheme S1) affect labeling efficiency characterized by occupancy. The first step is ligand binding that is followed by the activation of the complex. The activation may be, for example, a conformational change or a proton transfer. Then the covalent transformation is assumed to include a single rate limiting step. This may be, for example, the thiolate attack on a Michael acceptor. This may be followed by other steps leading to the final product, however, these steps are assumed to be fast enough not to affect the overall reaction rate.

$$\mathrm{P + L} \underset{k_{-1}}{\overset{k_1}{\rightleftharpoons}} \mathrm{P{\bullet}L} \underset{k_{-2}}{\overset{k_2}{\rightleftharpoons}} \mathrm{P'{\bullet}L} \xrightarrow{k_{inact}} \mathrm{PL}$$

Scheme S1 Three-step process of ligand-protein binding. The second step may be a conformational change or a proton transfer.

It is assumed that the concentration of the non-covalent complex (P•L) and the activated non-covalent complex (P′•L) is constant (steady state assumption).

$$0 = \frac{d[P'{\bullet}L]}{dt} = k_2[P{\bullet}L] - (k_{-2} + k_{inact})[P{\bullet}L]$$

$$[P{\bullet}L] = \frac{(k_{-2} + k_{inact})}{k_2}[P{\bullet}L]$$

$$0 = \frac{d[P{\bullet}L]}{dt} = k_1[P][L] + k_{-2}[P'{\bullet}L] - (k_{-1} + k_2)[P{\bullet}L]$$

$$[P] = \frac{(k_{-1} + k_2)[P{\bullet}L] - k_{-2}[P'{\bullet}L]}{k_1[L]} = \frac{(k_{-1} + k_2)\frac{(k_{-2} + k_{inact})}{k_2} - k_{-2}}{k_1[L]}[P'{\bullet}L]$$

One can obtain the observed first order reaction rate, k_{obs}, as follows

$$\frac{d[P'L]}{dt} = k_{inact}[P'{\bullet}L]$$

$$\frac{d[P_T]}{dt} = \frac{d[P_{cat}]}{dt} + \frac{d[P'L]}{dt} = 0$$

$$[P_{cat}] = [P] + [P{\bullet}L] + [P'{\bullet}L] = \left\{\frac{(k_{-1} + k_2)\frac{(k_{-2} + k_{inact})}{k_2} - k_{-2}}{k_1[L]} + \frac{(k_{-2} + k_{inact})}{k_2} + 1\right\}[P'{\bullet}L]$$

$$-\frac{d[P_{cat}]}{dt} = k_{inact}\frac{[P_{cat}]}{\frac{(k_{-1} + k_2)\frac{(k_{-2} + k_{inact})}{k_2} - k_{-2}}{k_1[L]} + \frac{(k_{-2} + k_{inact})}{k_2} + 1} = k_{obs}[P_{cat}]$$

This gives the expression for k_{obs}

$$k_{obs} = \frac{k_3}{\frac{(k_{-1} + k_2)\frac{(k_{-2} + k_3)}{k_2} - k_{-2}}{k_1[L]} + \frac{(k_{-2} + k_3)}{k_2} + 1}$$

When the chemical reaction is significantly slower than the "deactivation" of the complex, e.g. the protonation of the thiolate if the second step is thiol deprotonation, then $k_{inact} \ll k_{-2}$, and k_{obs} simplifies to

$$k_{obs} = \frac{k_{inact}}{\frac{K_1 K_2}{[L]} + K_2 + 1}$$

with $K_1 = \frac{k_{-1}}{k_1}$ and $K_2 = \frac{k_{-2}}{k_2}$.

When the second step is cysteine deprotonation then K_2 can be written as $K_2 = 10^{pK_a - pH}$.

Measuring k_{obs} as a function of ligand concentration at several pH values, K_1 and k_{inact} can be obtained by fitting to $k_{obs} - [L]$.

When pK_a is higher than the pH then $K_2 \gg 1$ and $k_{obs} = \frac{1}{\frac{K_1}{[L]}+1}\frac{k_{inact}}{K_2}$ showing that k_{obs} is reduced to that of the two-step model with $K_1^{3step} = K_I^{2step}$ and $k_{inact}^{2step} = \frac{k_{inact}^{3step}}{K_2}$.

7. Variation of the inactivation rate constant in the protein environment

Table S2 Inactivation rate of an acrylamide derivative (compound 11 of ref. [12]) toward kinases with Cys at the same location. Data are from ref. [12]

protein	k_{inact} (s^{-1})
JAK3	2.32
ITK	0.00564
BMX	0.000144
TXK	0.000487
TEC	0.00156
BTK	0.124
BLK	0.0278

8. Parameter derivation for the pH dependent labeling of KRAS^{G12C} by ARS-853

Table S3 pH dependent parameters of KRASG12C labeling by ARS-853 from ref [13]

pH	k_{inact} (s^{-1})	K_I (μM)
7.0	0.0073	36.8
7.5	0.0169	30.2
8.0	0.038	34.2
8.5	0.099	42.6
9.0	0.280	77.0

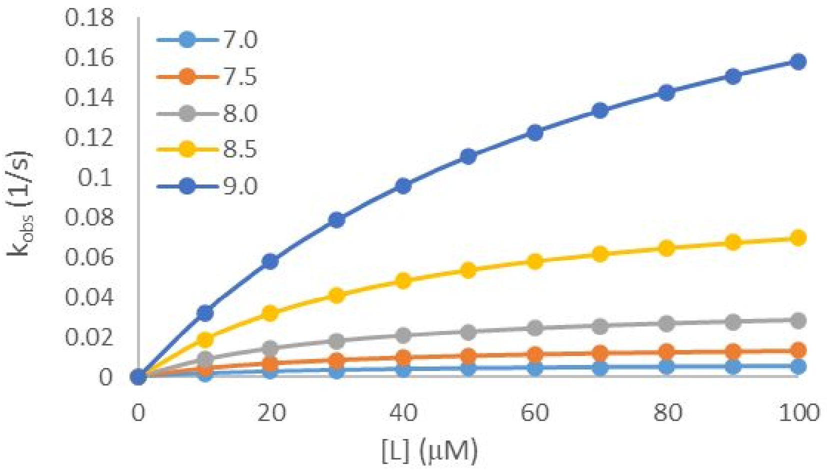

Figure S7 Observed first order rate constant (k_{obs}) as a function of ligand concentration $[L]$ at several pH values generated with data in Table S2 using Eq. (2) of the main text.

Table S4 k_{inact} and K_I fitted to k_{obs} of Figure S4 using $k_{obs} = \frac{k_{inact}}{\frac{K_1 K_2}{[L]} + K_2 + 1}$ of the three-step model at pK_a=9.2.

pH	7.0	7.5	8.0	8.5	9.0
k_{inact} (s^{-1})	1.16	0.86	0.64	0.60	0.72
K_d (μM)	37.0	30.8	36.4	51.1	125.6

mean k_{inact}=0.80 s^{-1}; mean K_d=56.2 μM

9. Linear relationship between IC_{50} and K_I/k_{inact} for $KRAS^{G12C}$ inhibitors

Table S5 IC_{50} and K_I/k_{inact} for $KRAS^{G12C}$ inhibitors from ref [14]

compound	IC_{50}(μM)	k_{inact}/K_I ($M^{-1}s^{-1}$)	K_I/k_{inact} (Ms)	IC_{50} (M)
1	3.1	2.6	3.85E-01	0.0000031
2	1.2	4	2.50E-01	0.0000012
3	0.3	11	9.09E-02	0.0000003
4	0.069	83	1.20E-02	6.9E-08
5	0.016	343	2.92E-03	1.6E-08
6	0.017	924	1.08E-03	1.7E-08
7	0.00065	6698	1.49E-04	6.5E-10
AMG510	0.00085	4895	2.04E-04	8.5E-10
MRTX849	0.00008	56284	1.78E-05	8E-11

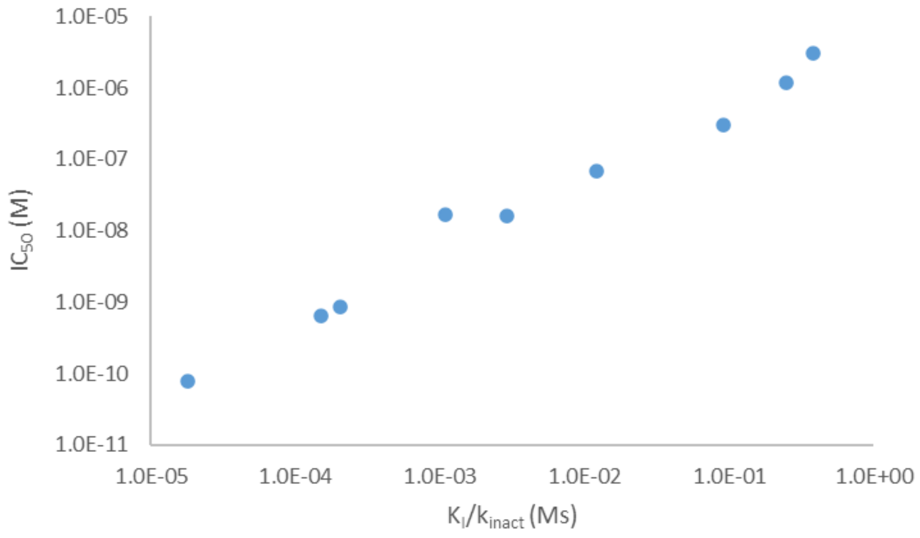

Figure S8 IC_{50} versus K_I/k_{inact} for $KRAS^{G12C}$ inhibitors show linear relationship. Data are from ref [14] and shown in Table S5

10. MS occupancy results

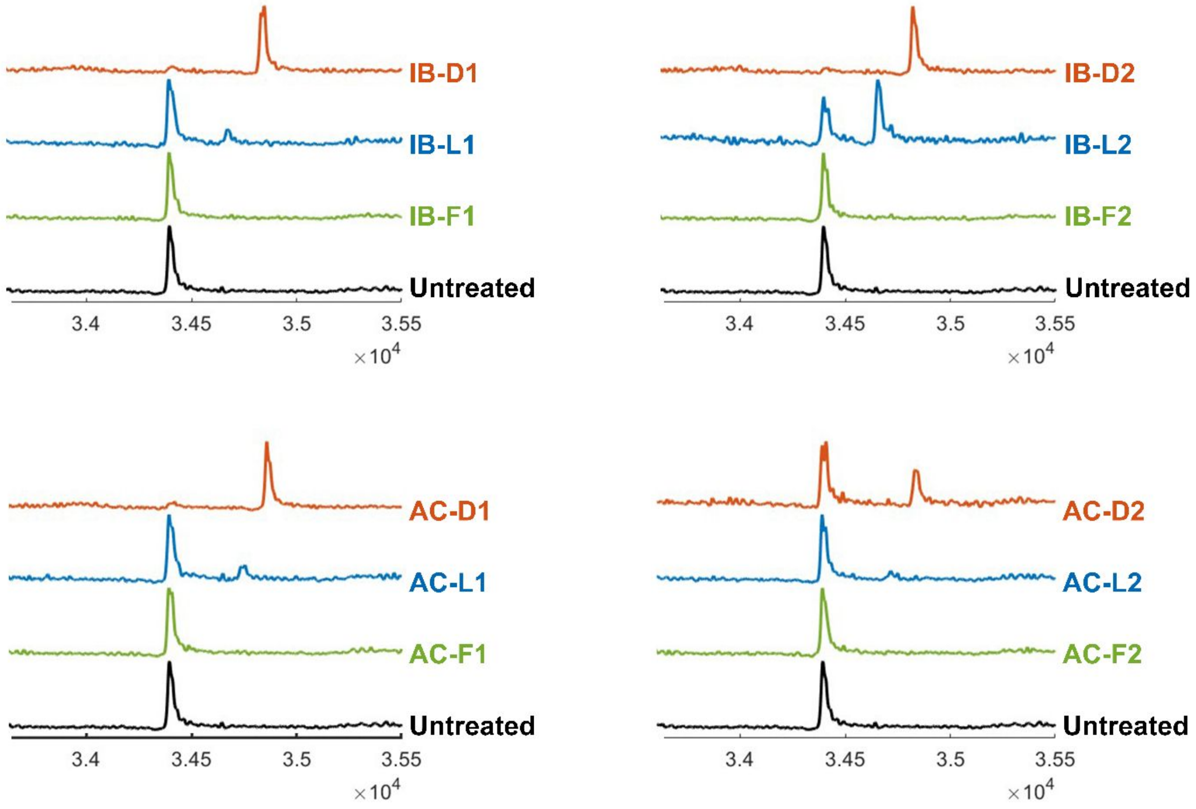

AD-F1

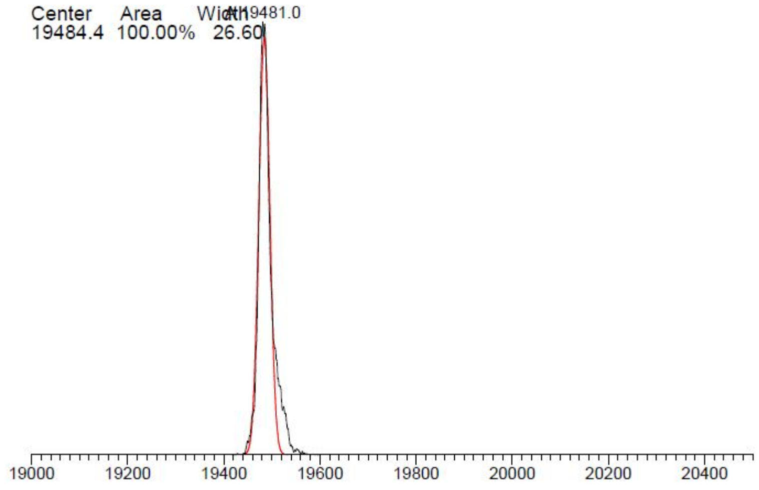

AD-F2

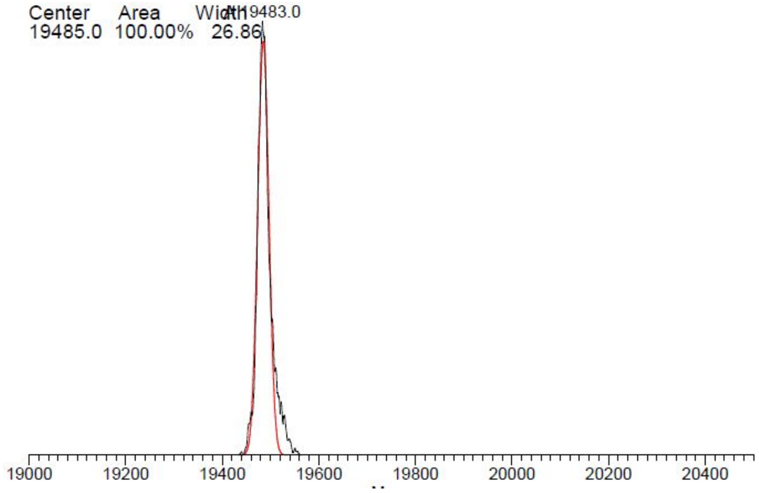

AD-L1

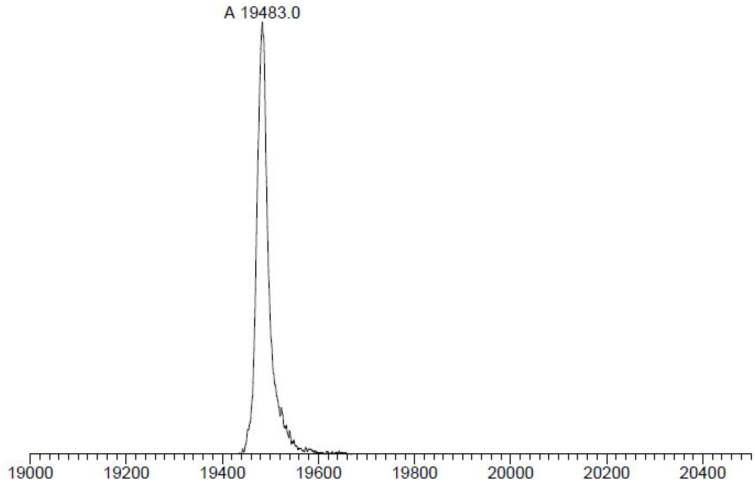

AD-L2

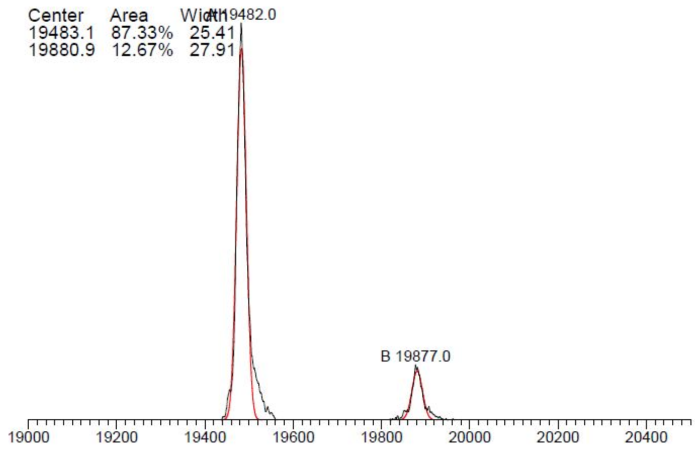

AD-D1

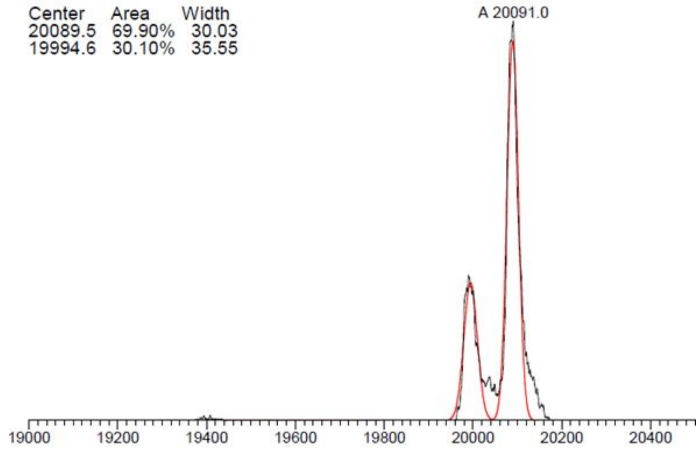

AD-D2

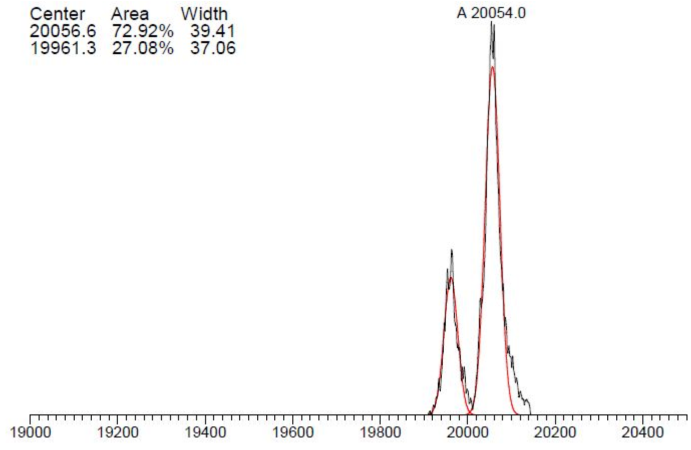

Figure S6 Single-concentration occupancy results, shown as deconvoluted MS spectra for all covalent probes of BTK and KRAS^{G12C}.

11. k_{inact}/K_I determination

Here, we present how k_{inact}/K_I were determined based on concentration-dependent occupancy curves.

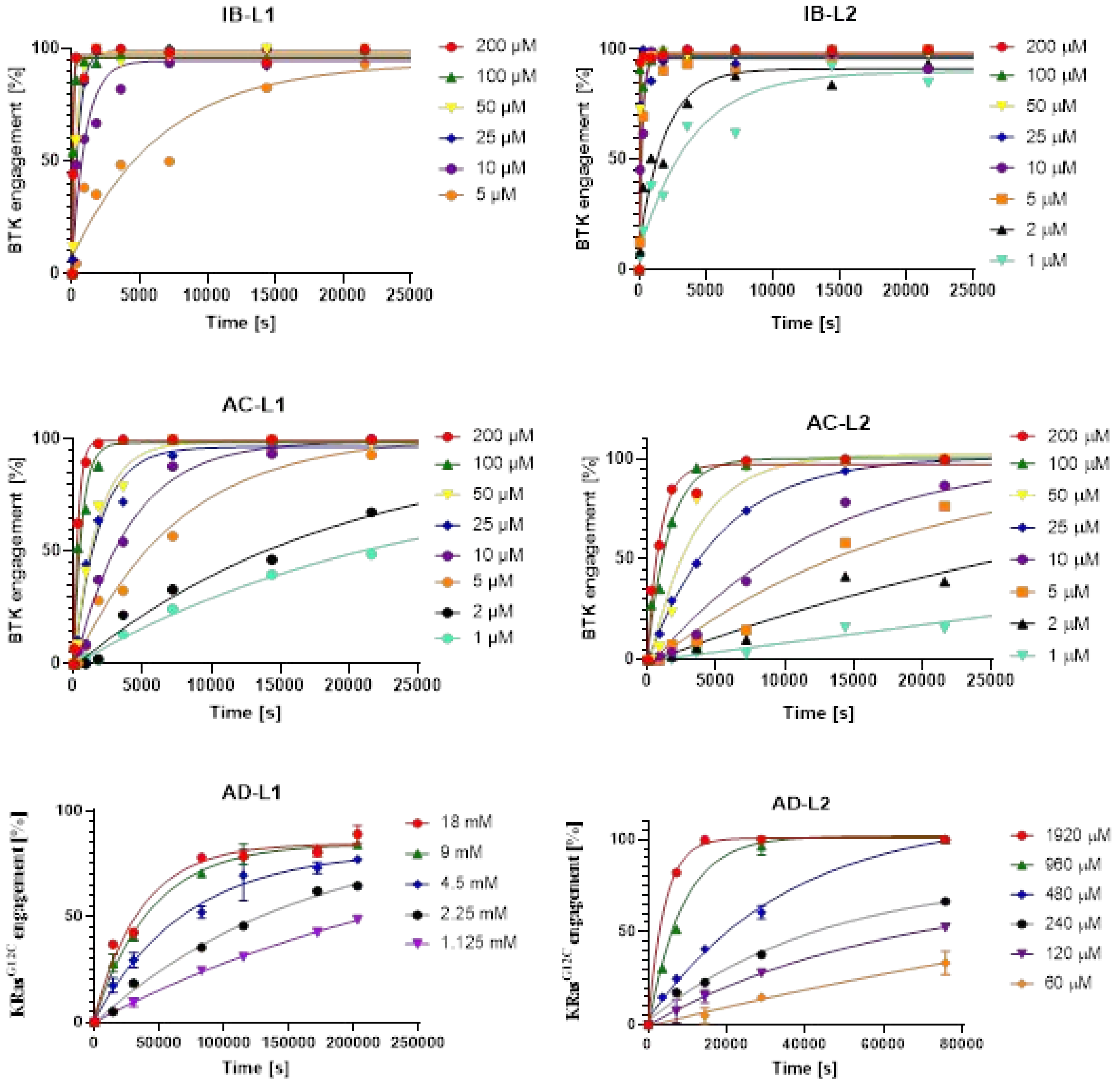

Figure S7 Time courses of BTK and $KRAS^{G12C}$ covalent engagement. For each compound, the percent of covalently labeled protein was assessed by MS and exponential one-phase decoy regression was used (with fixed zero percent labelling at time zero) to calculate the k_{obs} values.

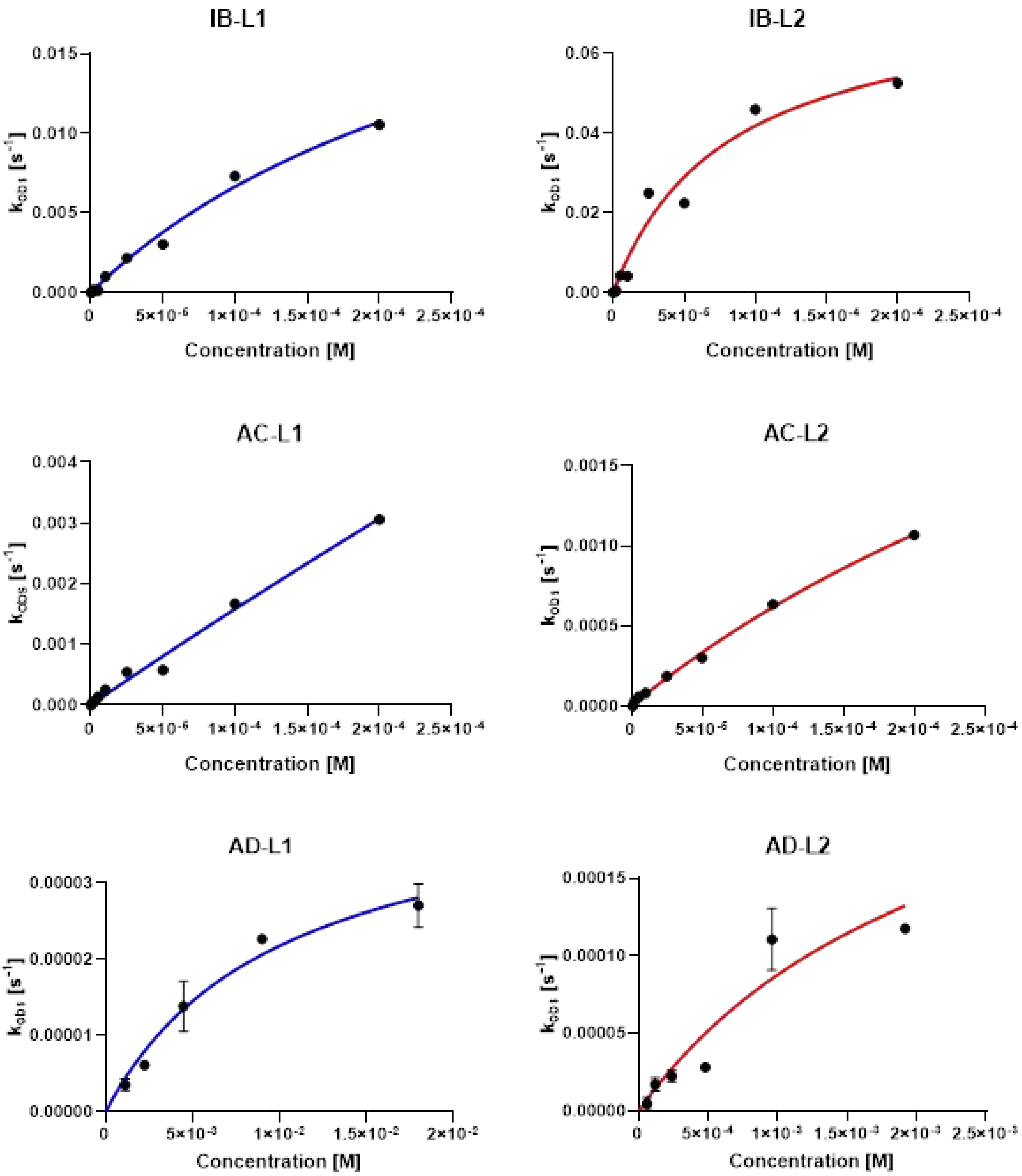

Figure S8 Determination of K_I and k_{inact} for the interaction of covalent probes with BTK and KRAS^{G12C}. Calculated k_{obs} values were plotted against the concentration of the probes and and K_I and k_{inact} were calculated directly from non-linear regression according to the k_{obs} – c function as follows: $k_{obs} = \frac{k_{inact} \cdot c}{K_I + c}$.

12. HPLC-MS purity of the synthesized covalent probes

The synthesized covalent probes are reported here by their HPLC-MS spectra. Others were purchased and used as commercially available chemicals.

IB-L1 (purity: 99+%)

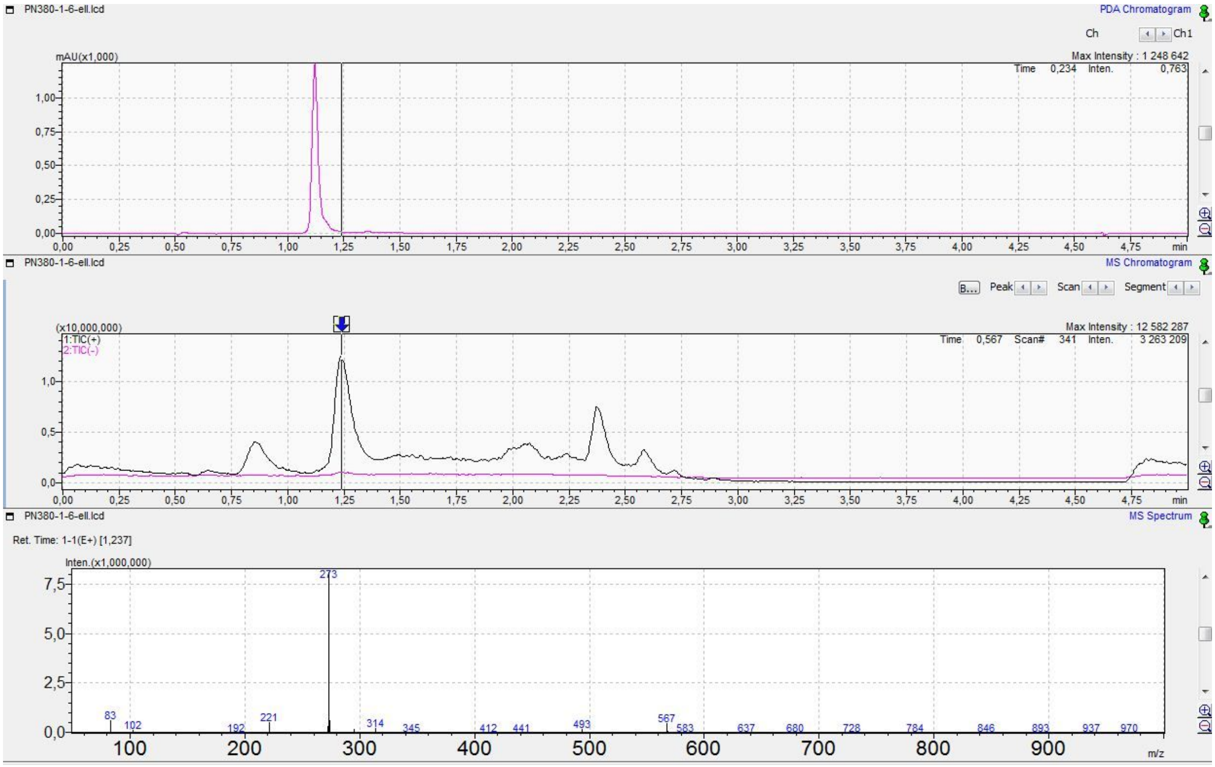

IB-L2 (purity: 97.2%)

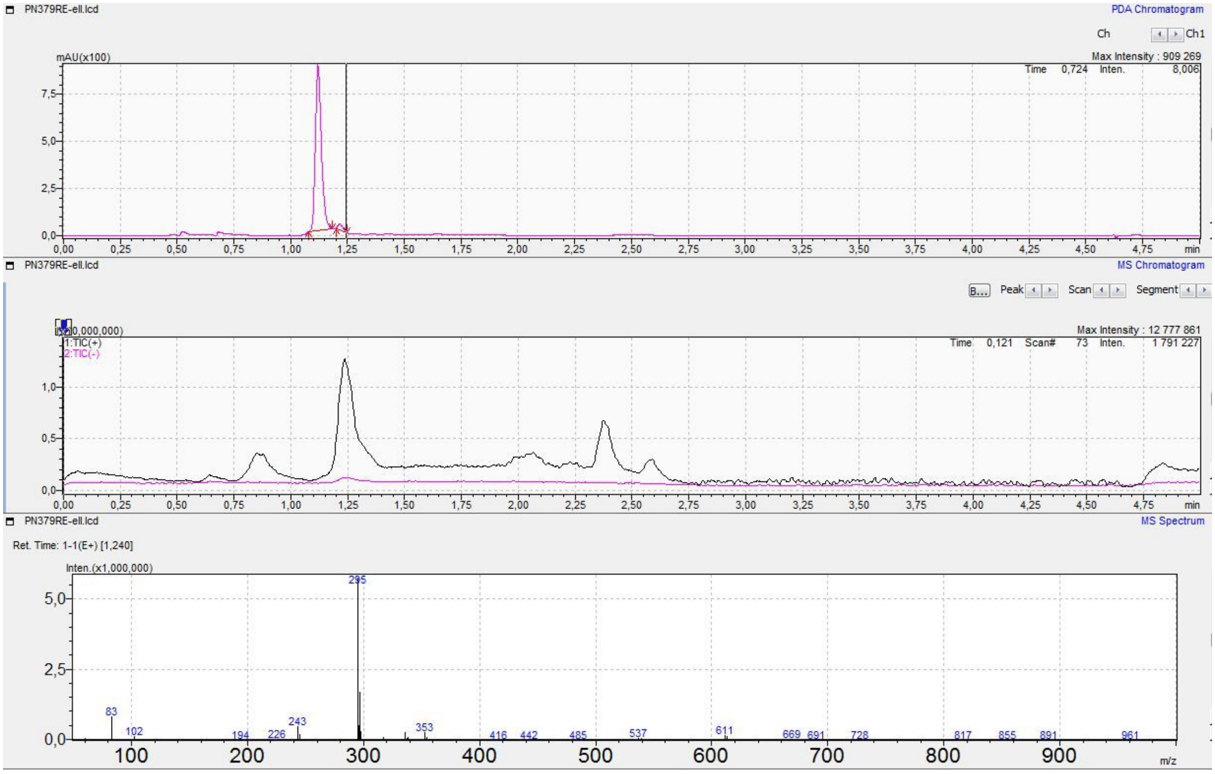

IB-D2 (purity: 99+%)

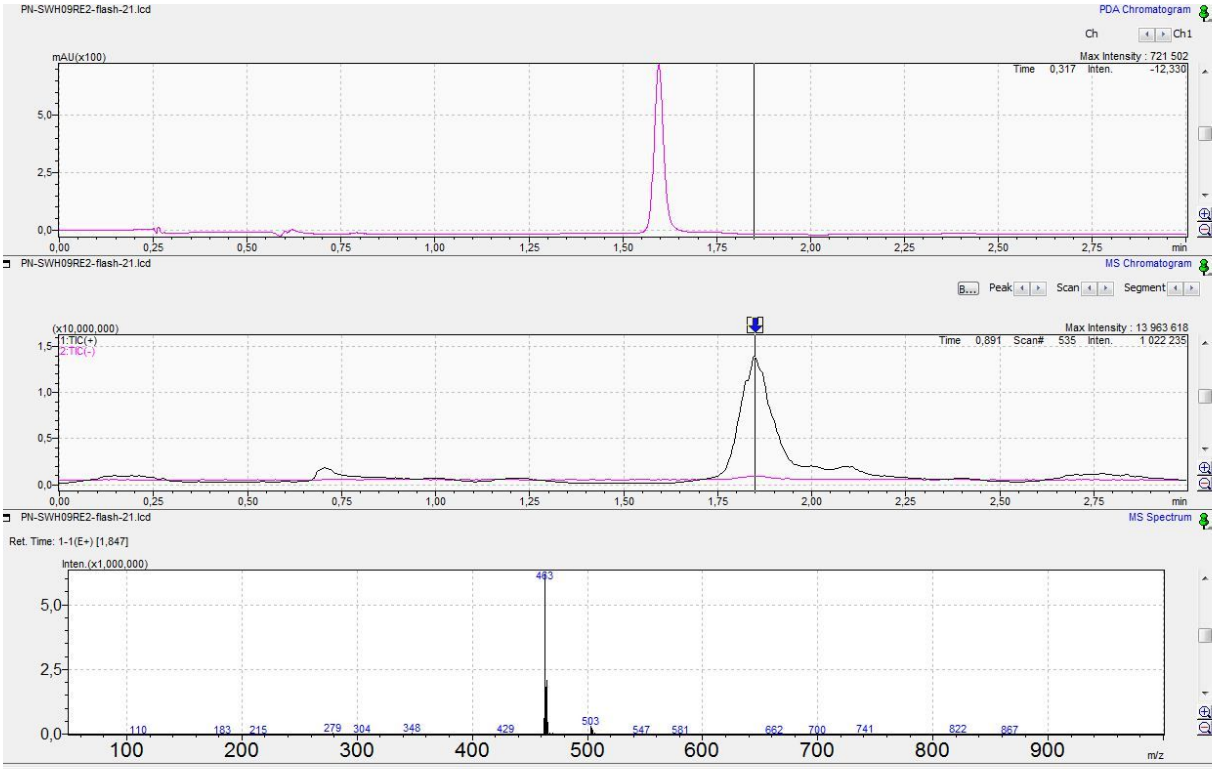

AC-L1 (purity: 99+%)

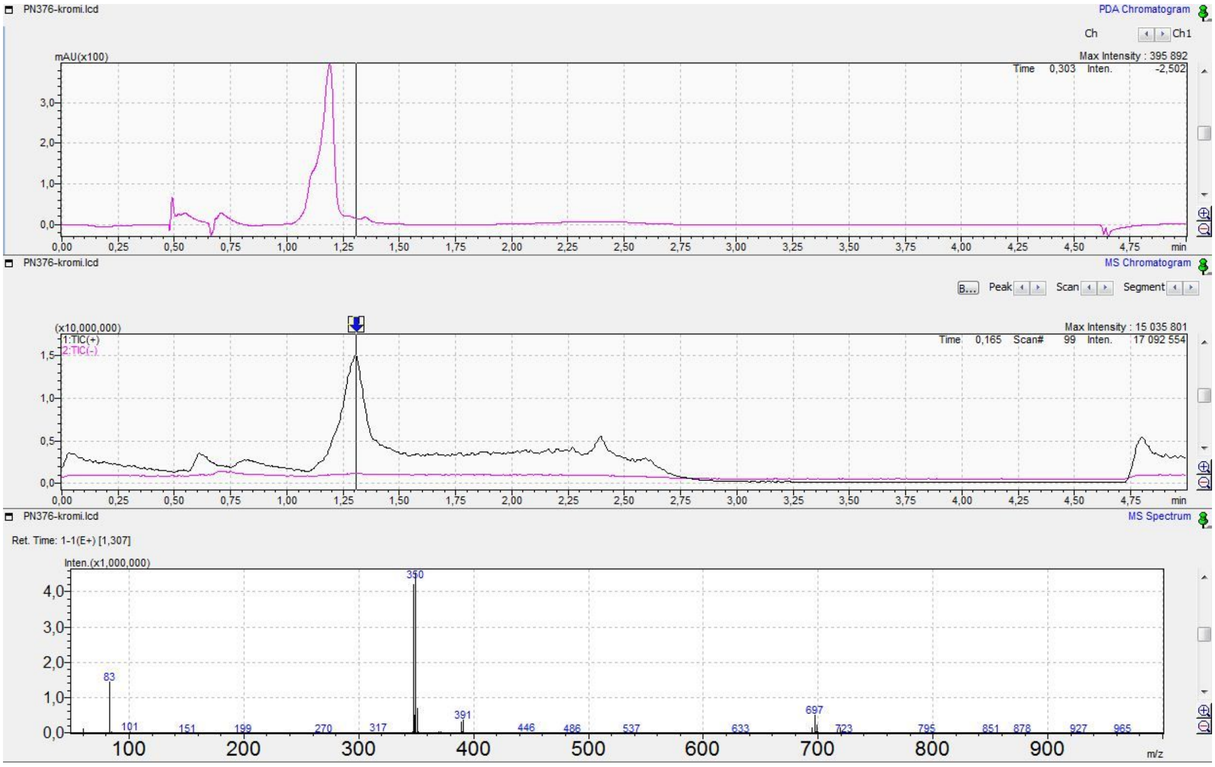

AC-L2 (purity: 99+%)

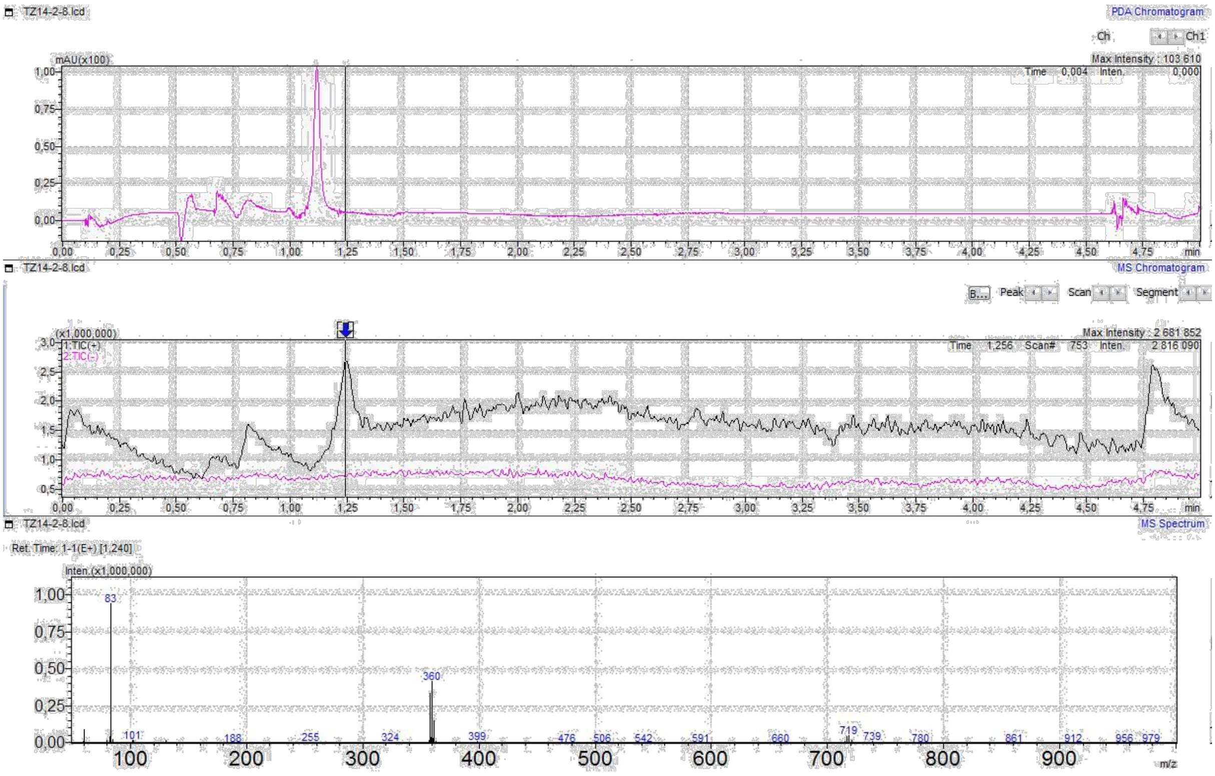

AC-D2 (purity: 99+%)

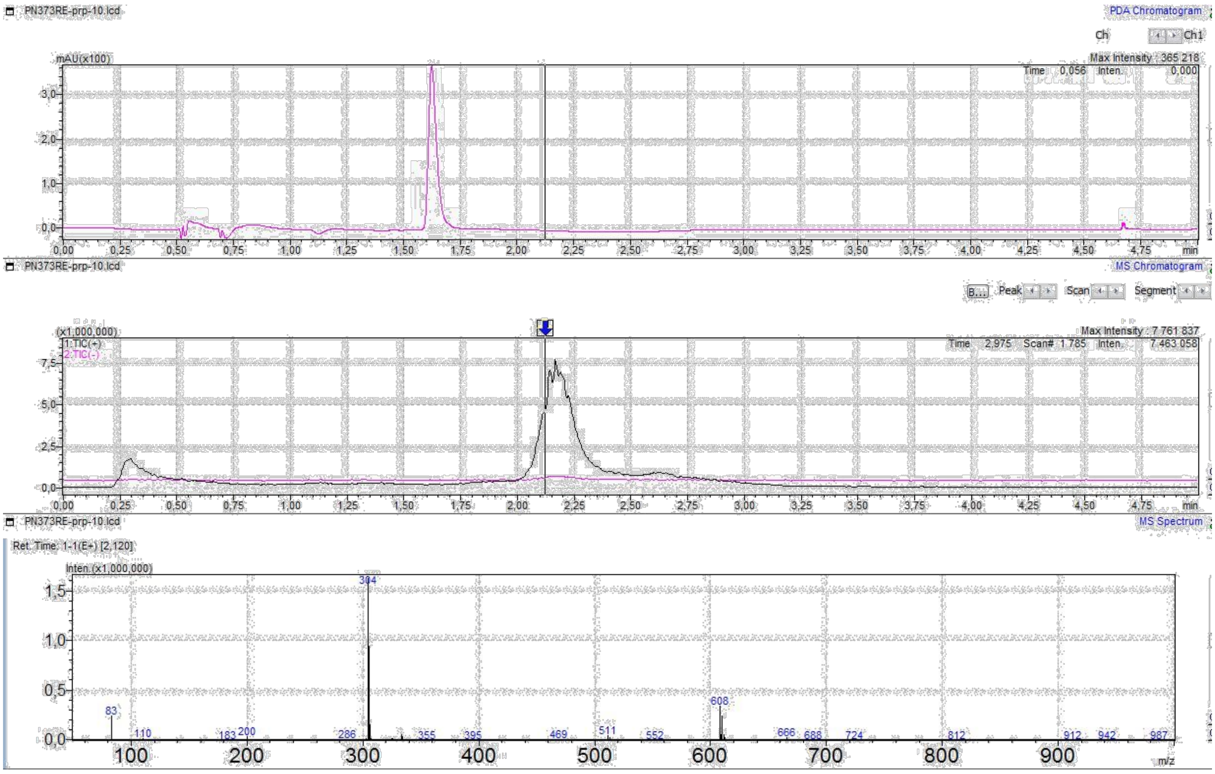

AD-F1 (purity: 99+%)

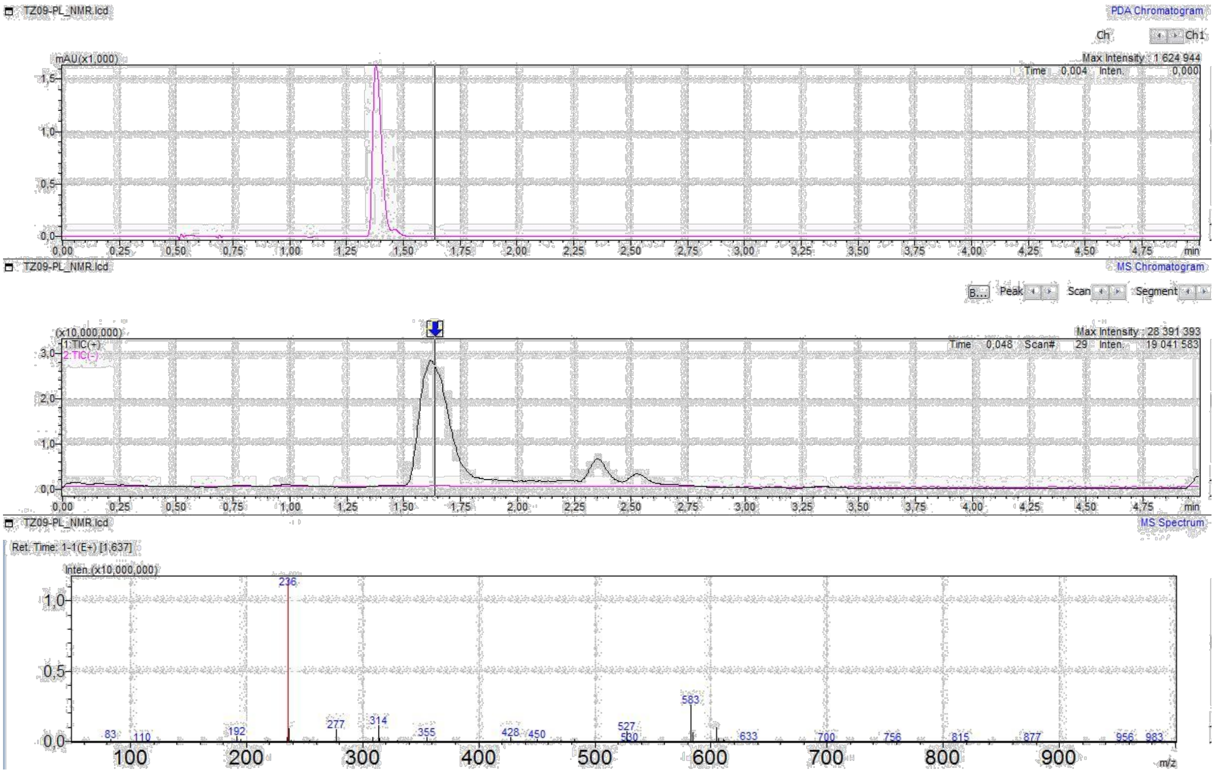

AD-F2 (purity: 99+%)

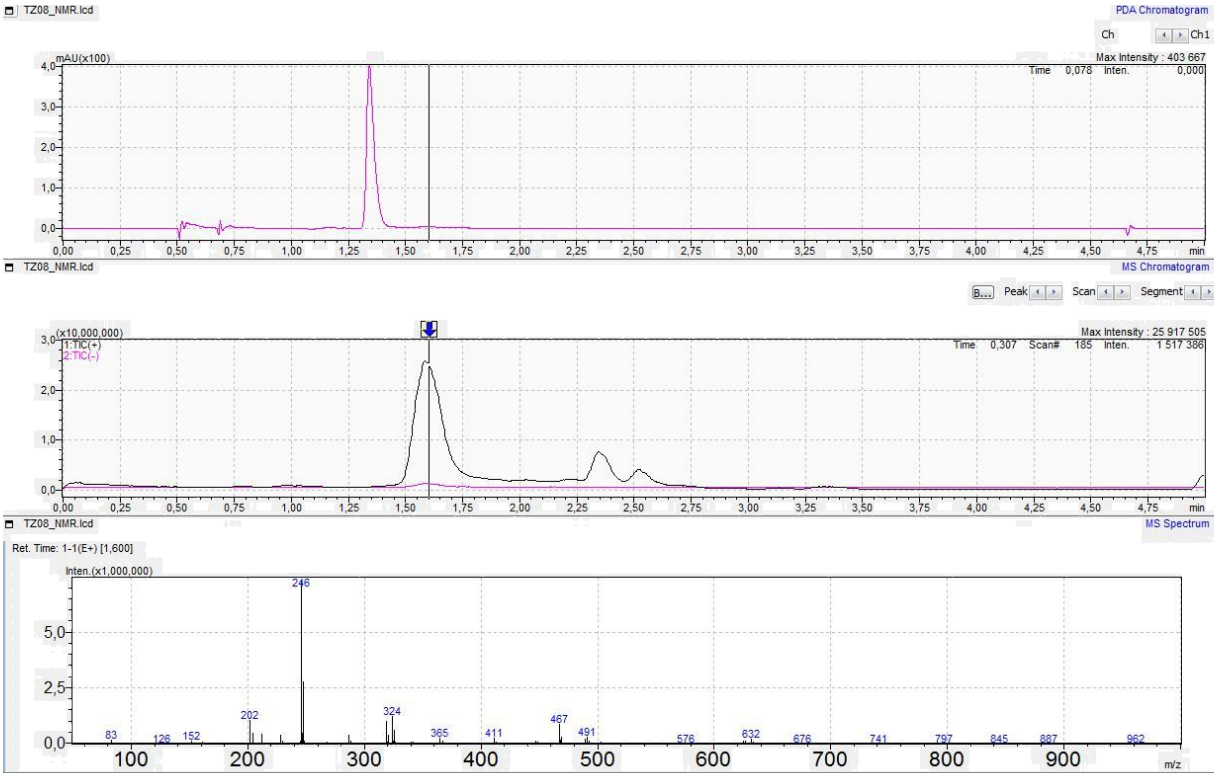

AD-L1 (purity: 99+%)

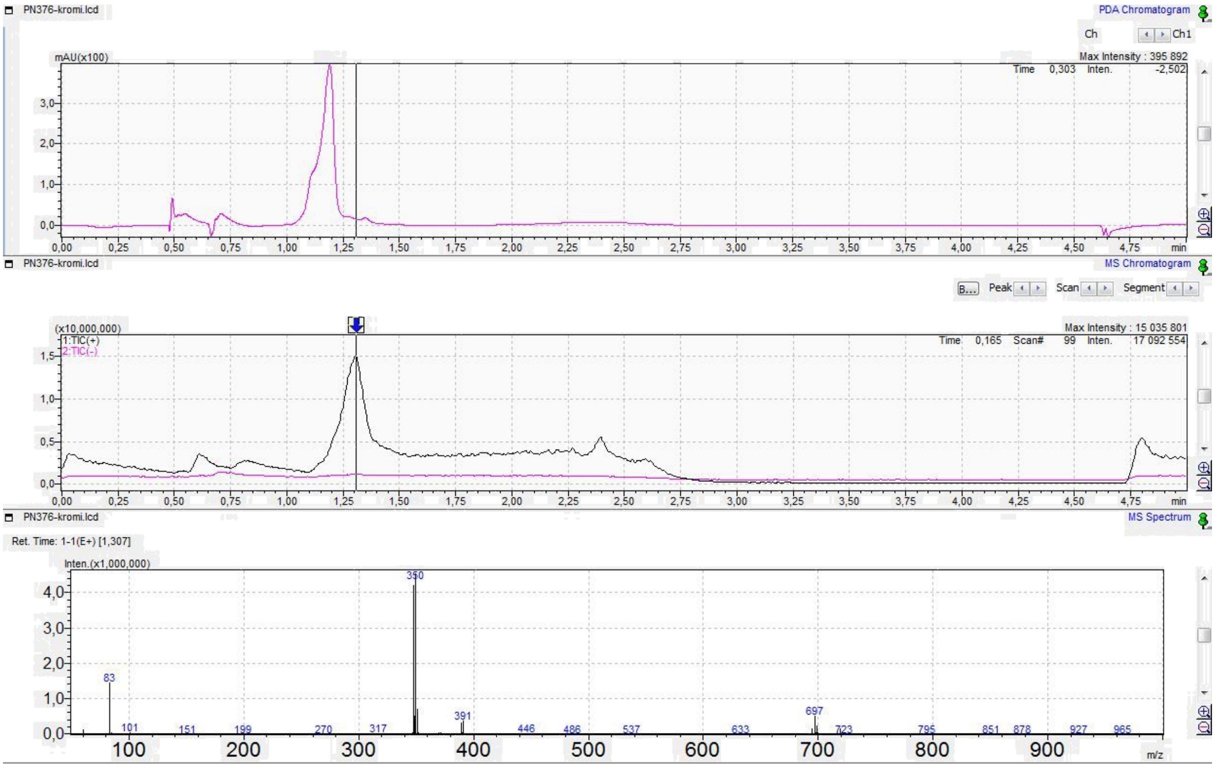

AD-L2 (purity: 95.3%)

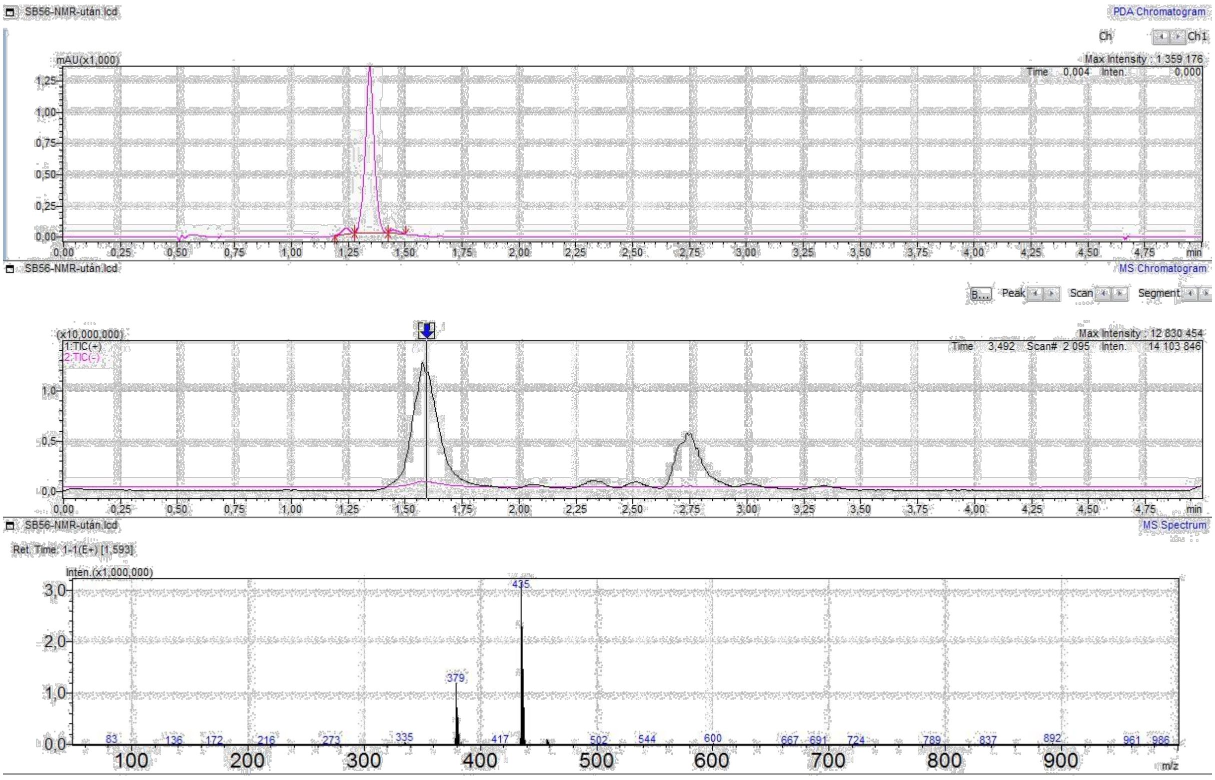

AD-D2 (purity: 99+%)

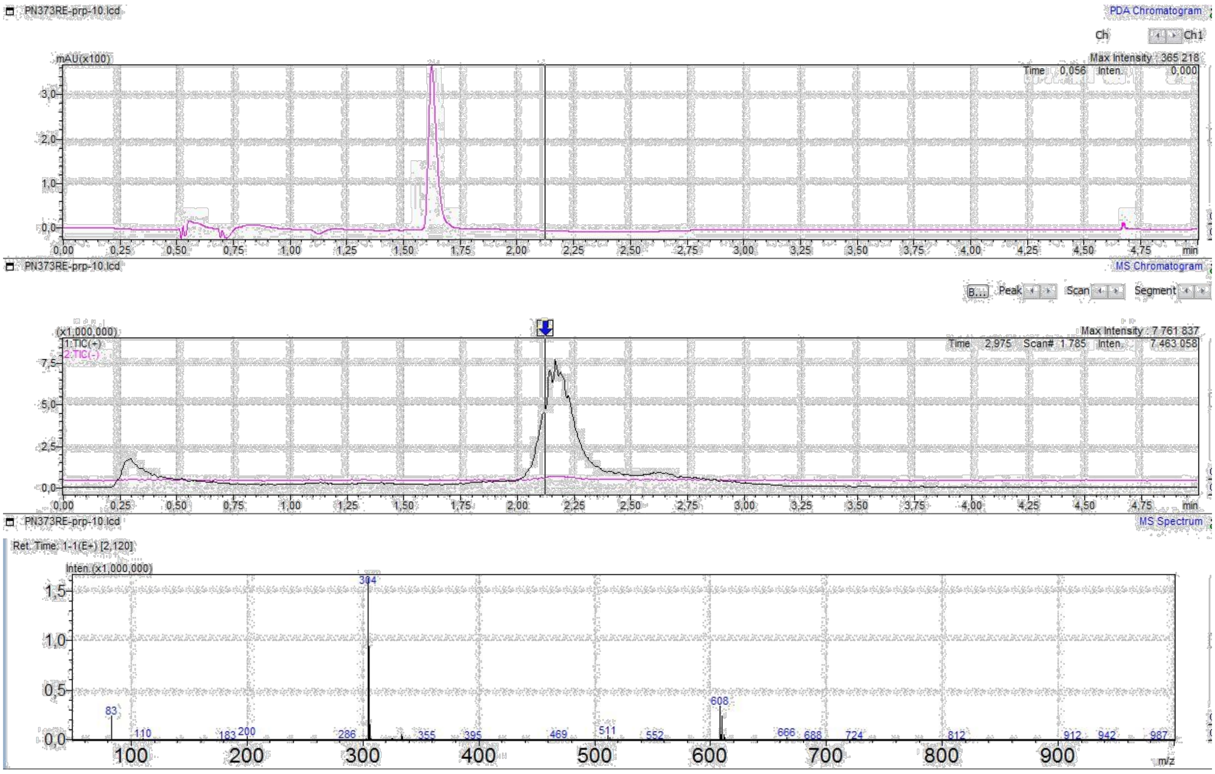

13. ^{1}H-NMR spectrum of the synthesized covalent probes

IB-L1

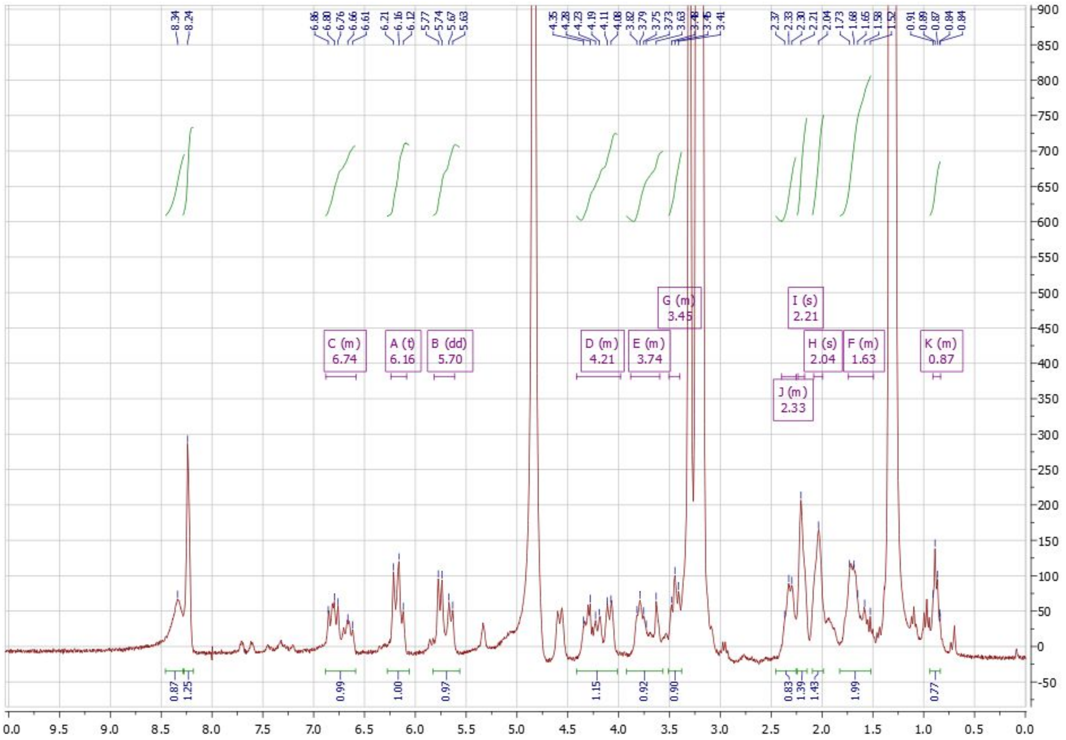

IB-L2

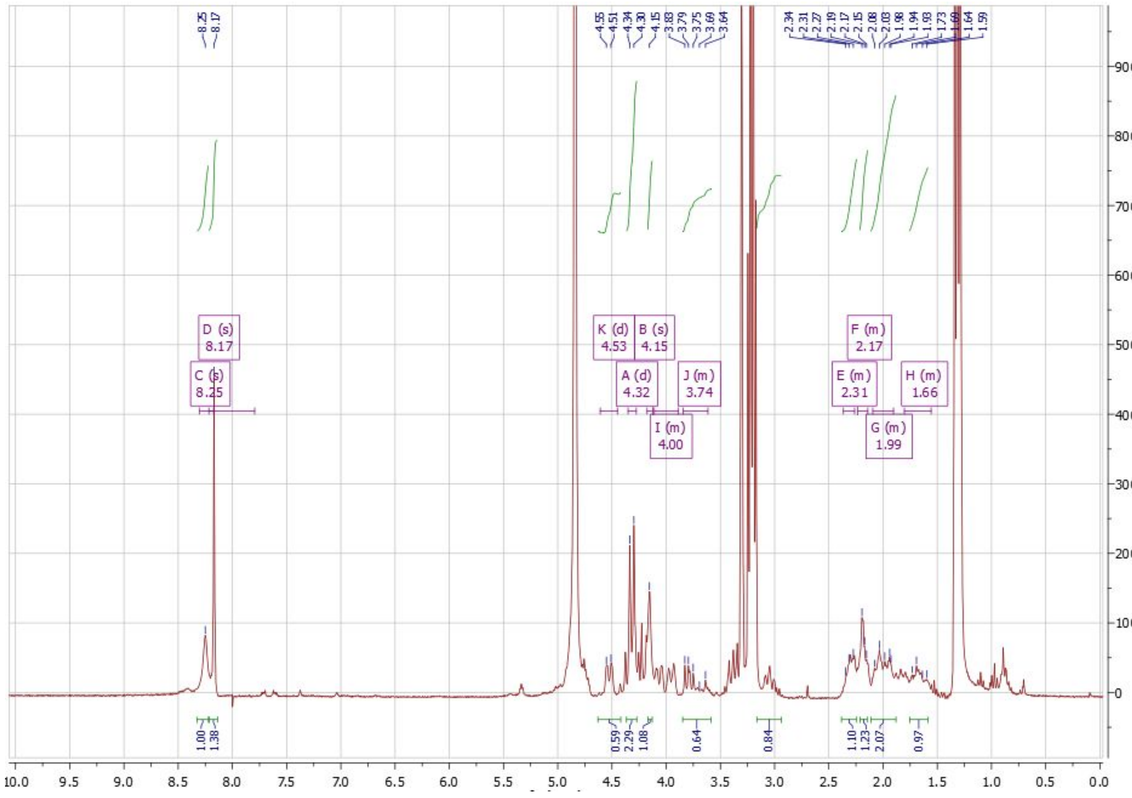

IB-D2

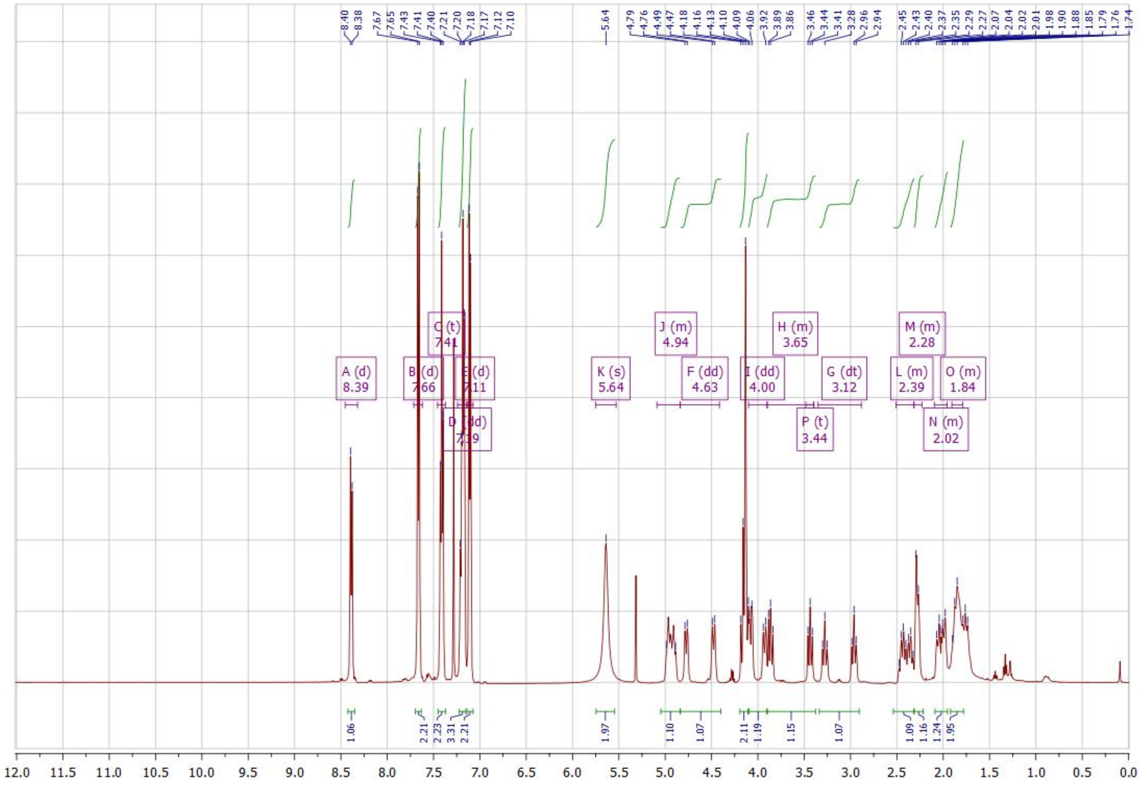

AC-L1

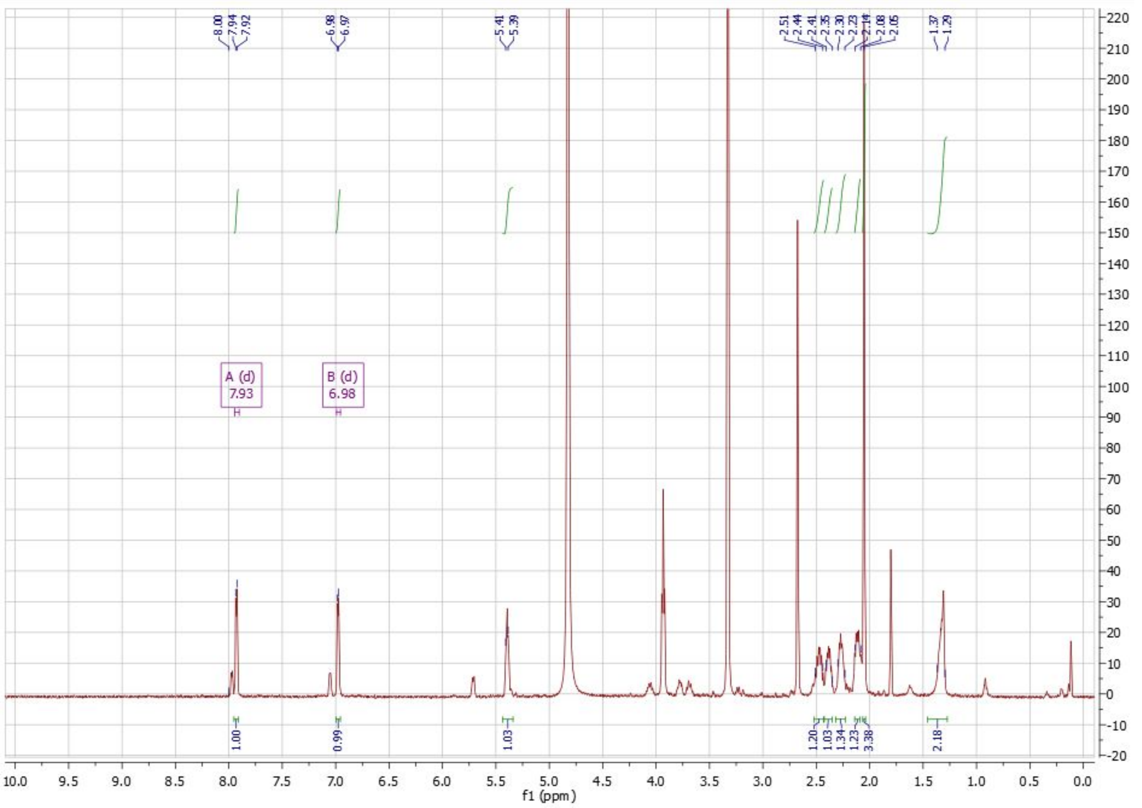

AC-L2

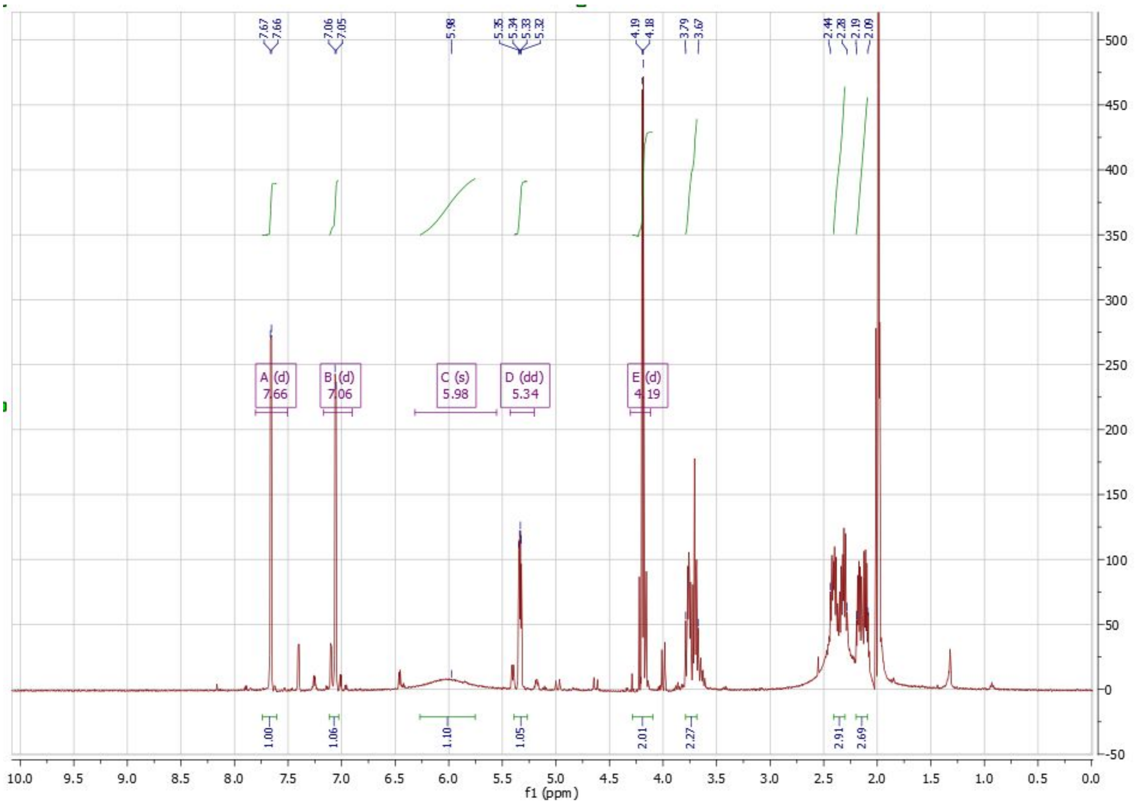

AC-D2

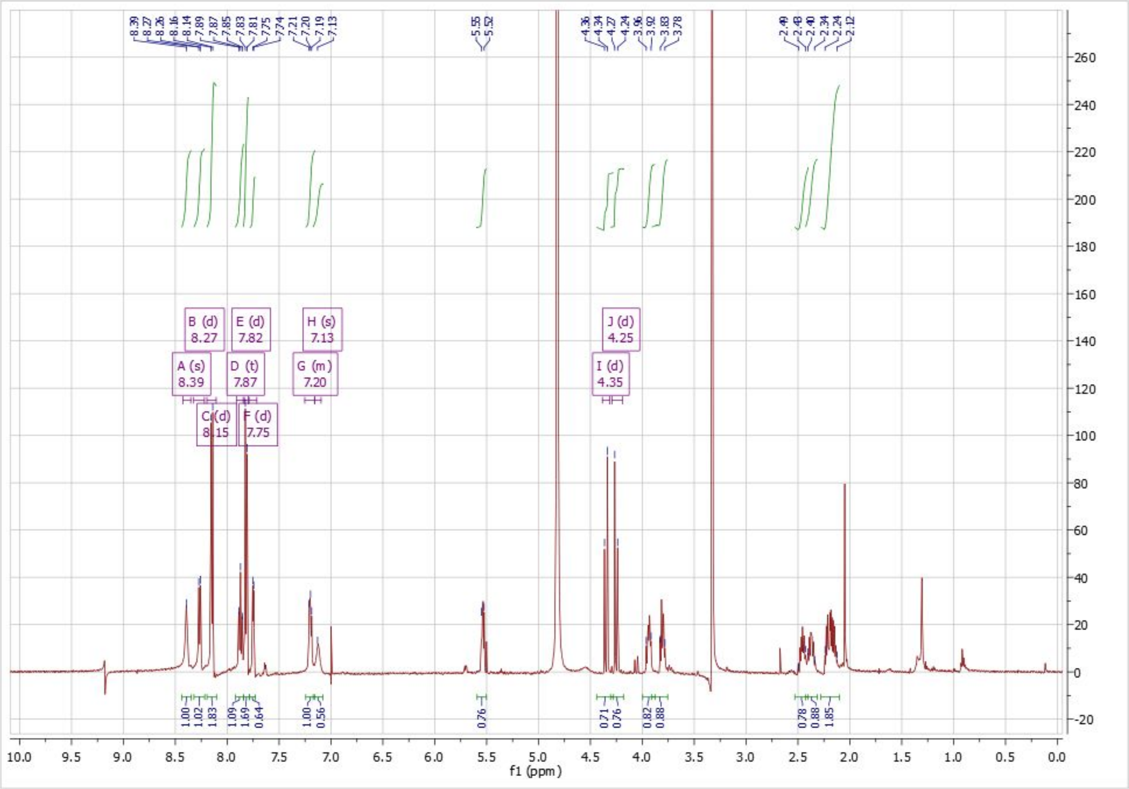

A (s)
8.39
B (d)
8.27
C (d)
8.15
D (t)
7.87
E (d)
7.82
F (d)
7.75
G (m)
7.20
H (s)
7.13
I (d)
4.35
J (d)
4.25
f1 (ppm)

AD-F1

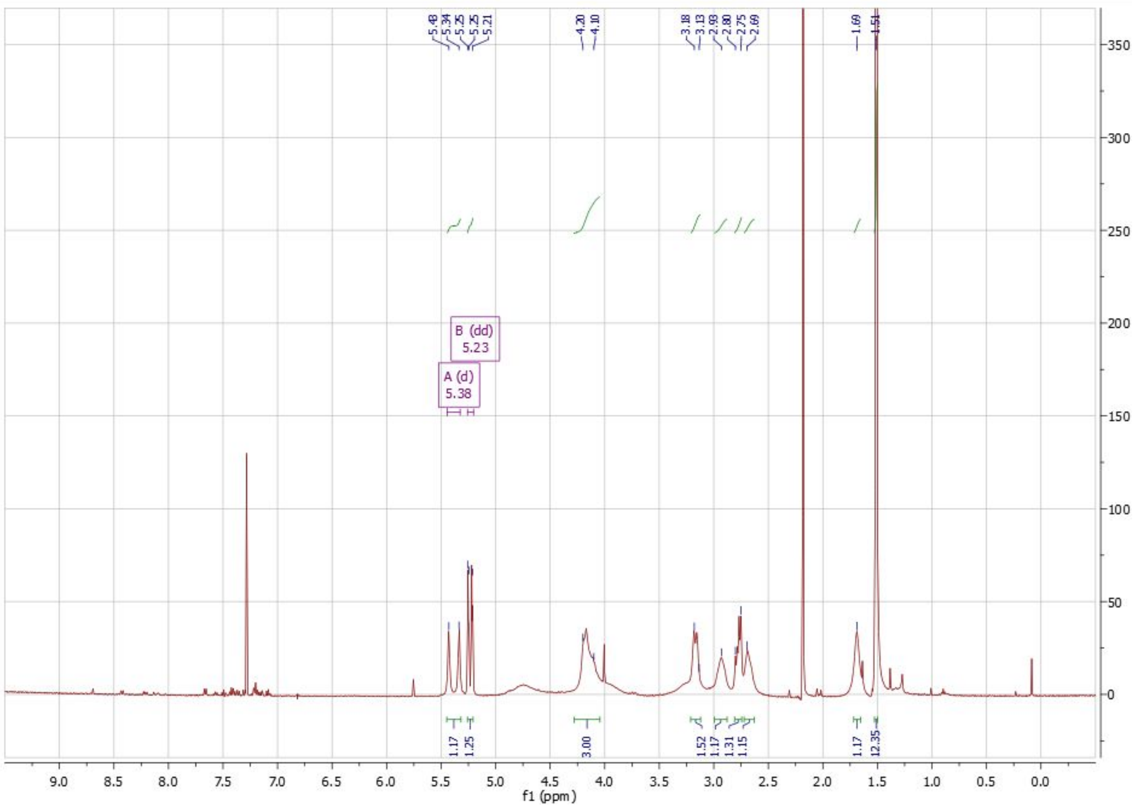

A (d)
5.38
B (dd)
5.23
f1 (ppm)

AD-F2

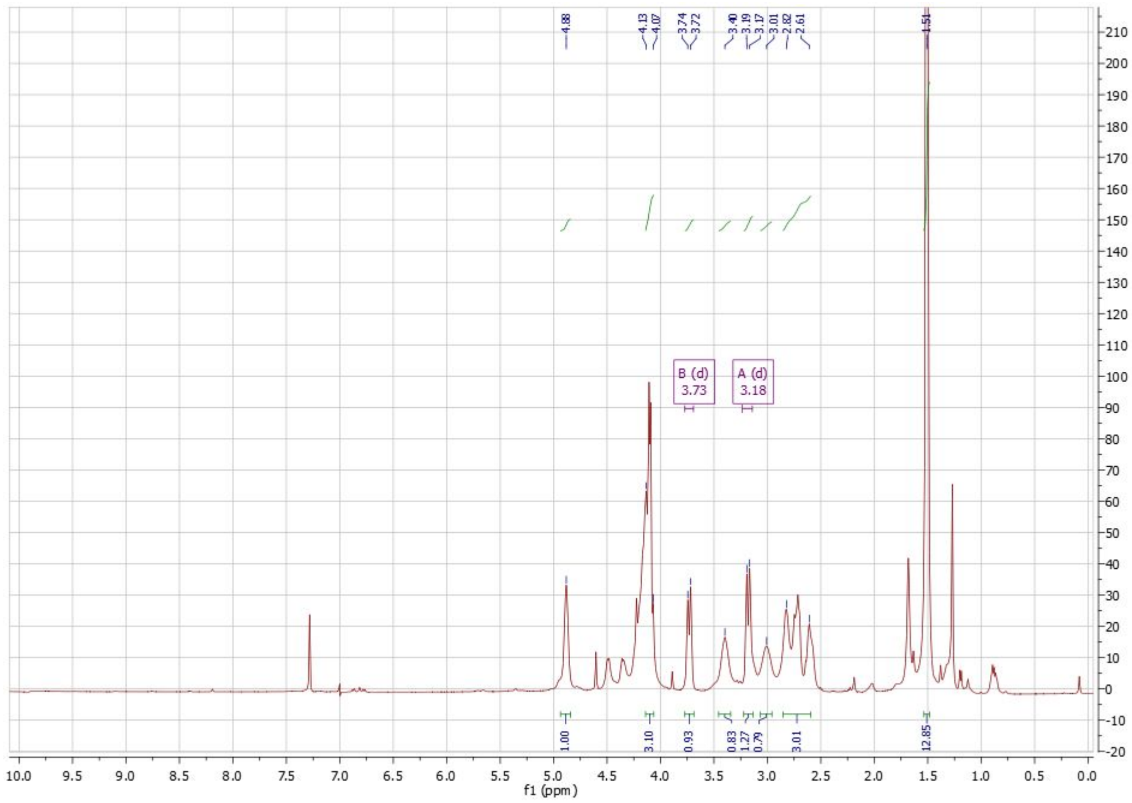

B (d)
3.73
A (d)
3.18
f1 (ppm)

AD-L1

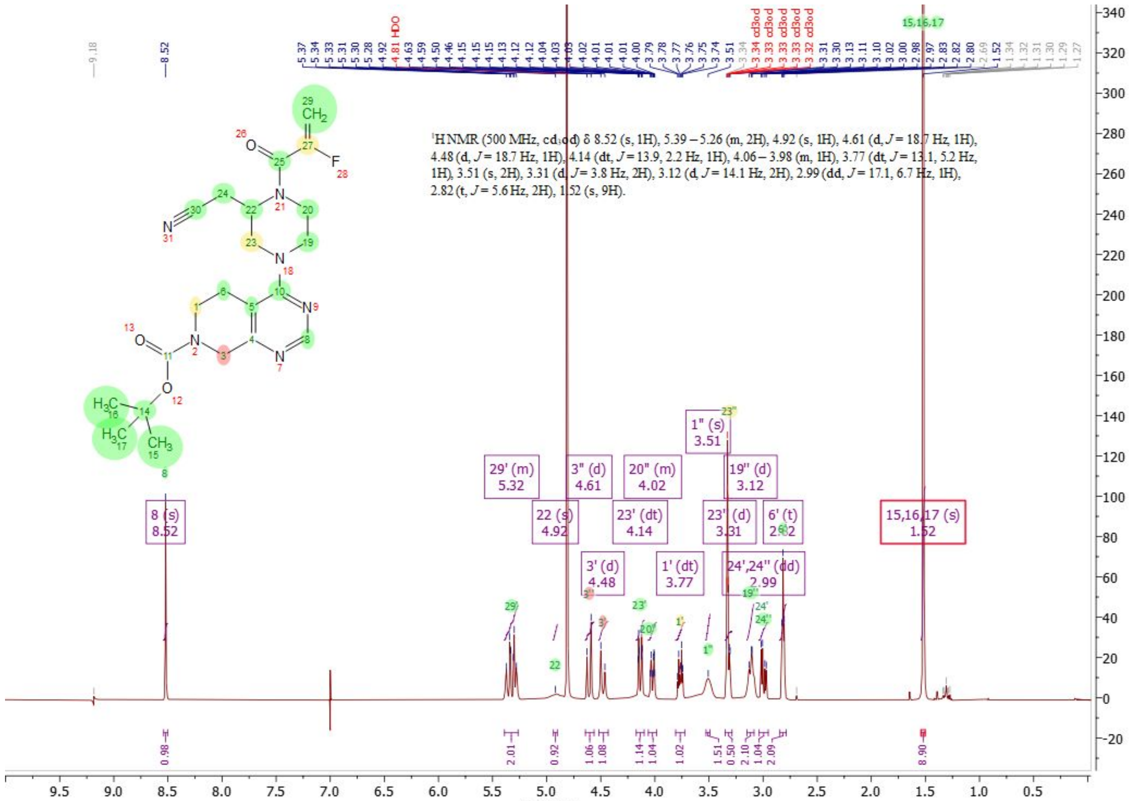

^{1}H NMR (500 MHz, cd3od) δ 8.52 (s, 1H), 5.39 – 5.26 (m, 2H), 4.92 (s, 1H), 4.61 (d, J = 18.7 Hz, 1H), 4.48 (d, J = 18.7 Hz, 1H), 4.14 (dt, J = 13.9, 2.2 Hz, 1H), 4.06 – 3.98 (m, 1H), 3.77 (dt, J = 13.1, 5.2 Hz, 1H), 3.51 (s, 2H), 3.31 (d, J = 3.8 Hz, 2H), 3.12 (d, J = 14.1 Hz, 2H), 2.99 (dd, J = 17.1, 6.7 Hz, 1H), 2.82 (t, J = 5.6 Hz, 2H), 1.52 (s, 9H).
8 (s)
8.52
29' (m)
5.32
22 (s)
4.92
3" (d)
4.61
3' (d)
4.48
20" (m)
4.02
23' (dt)
4.14
1' (dt)
3.77
1" (s)
3.51
23' (d)
3.31
19" (d)
3.12
24',24" (dd)
2.99
6' (t)
2.82
15,16,17 (s)
1.52
f1 (ppm)

AD-L2

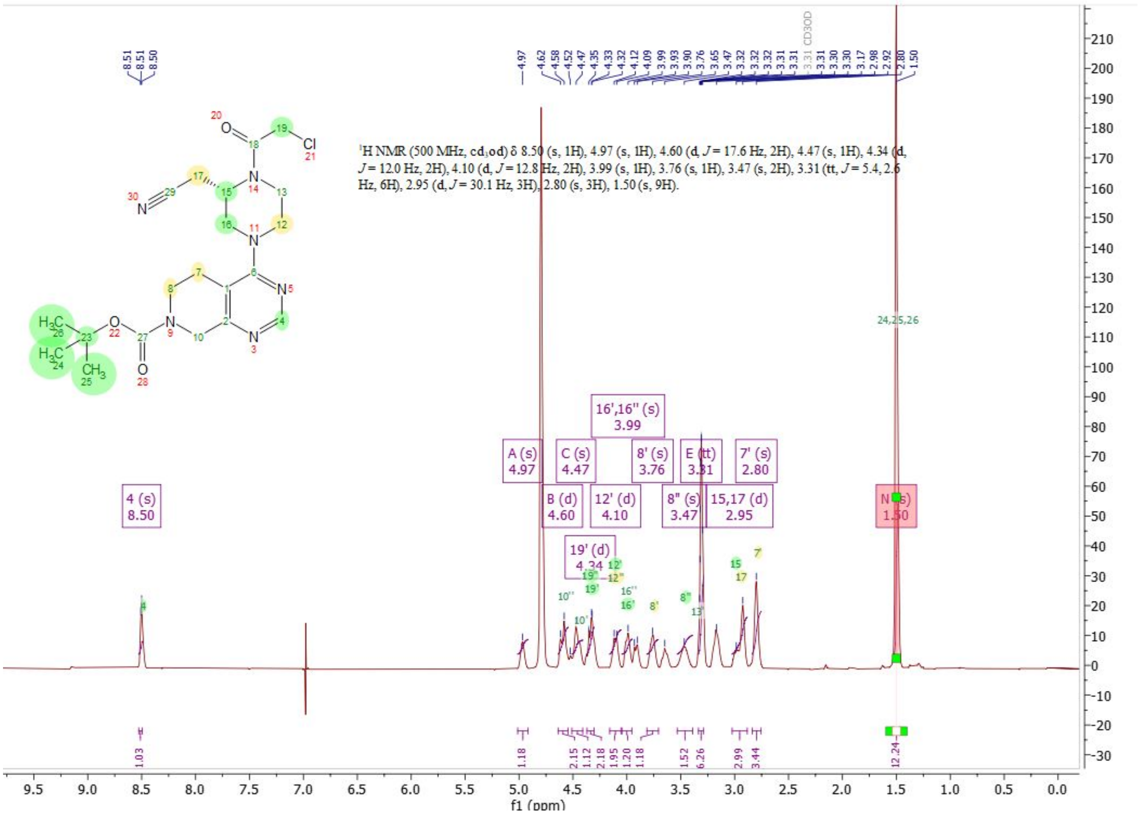

^{1}H NMR (500 MHz, cd$_3$od) δ 8.50 (s, 1H), 4.97 (s, 1H), 4.60 (d, J = 17.6 Hz, 2H), 4.47 (s, 1H), 4.34 (d, J = 12.0 Hz, 2H), 4.10 (d, J = 12.8 Hz, 2H), 3.99 (s, 1H), 3.76 (s, 1H), 3.47 (s, 2H), 3.31 (tt, J = 5.4, 2.6 Hz, 6H), 2.95 (d, J = 30.1 Hz, 3H), 2.80 (s, 3H), 1.50 (s, 9H).
f1 (ppm)

AD-D2

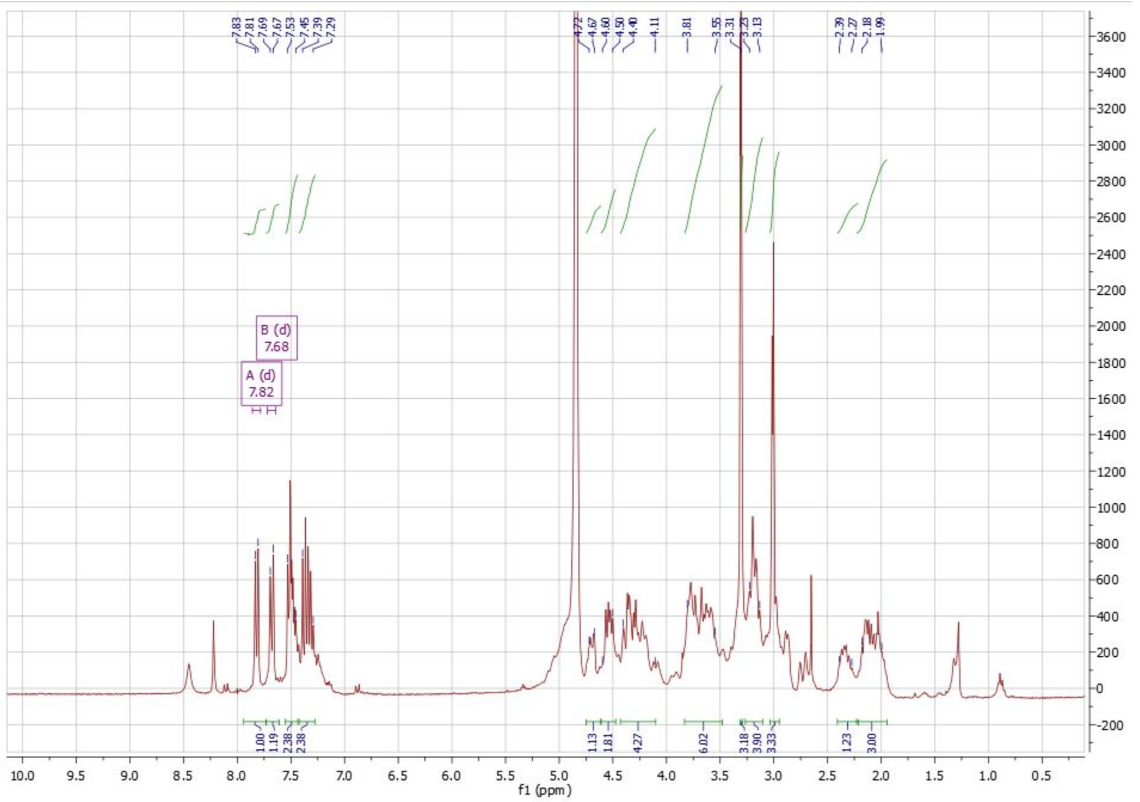

A (d) 7.82
B (d) 7.68
f1 (ppm)

AC-int

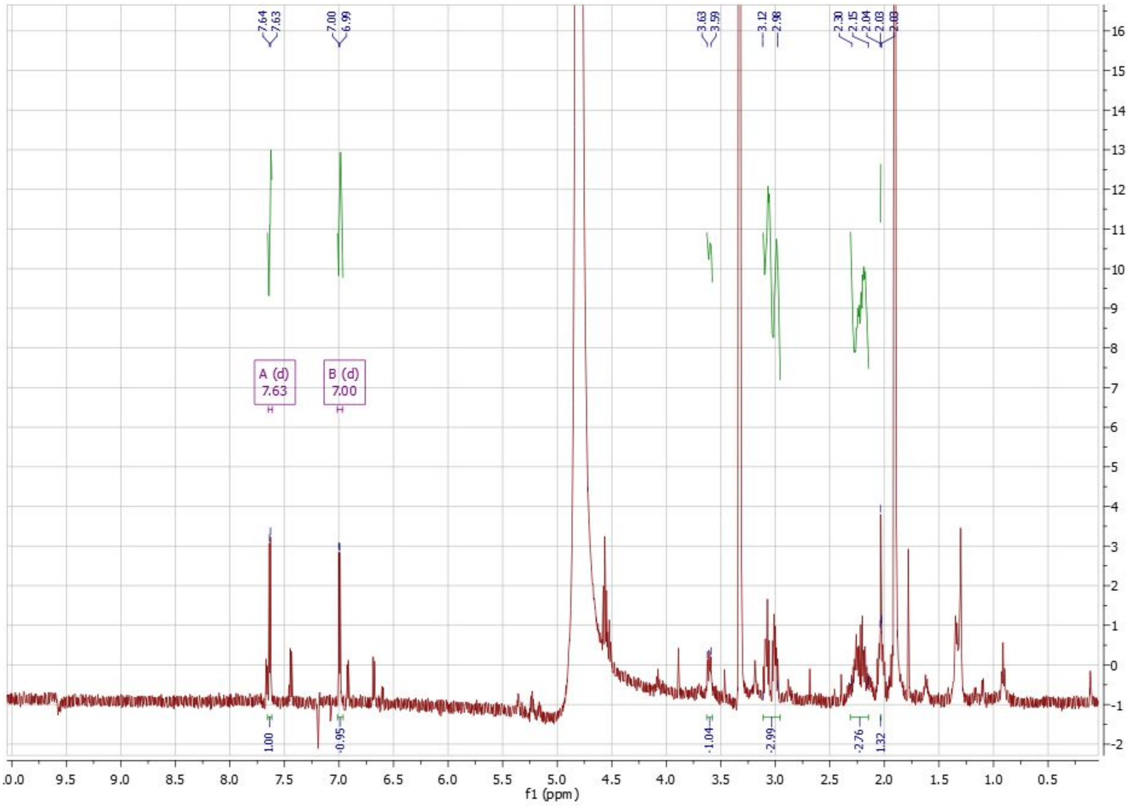
A (d)
7.63
B (d)
7.00
f1 (ppm)

AD-int

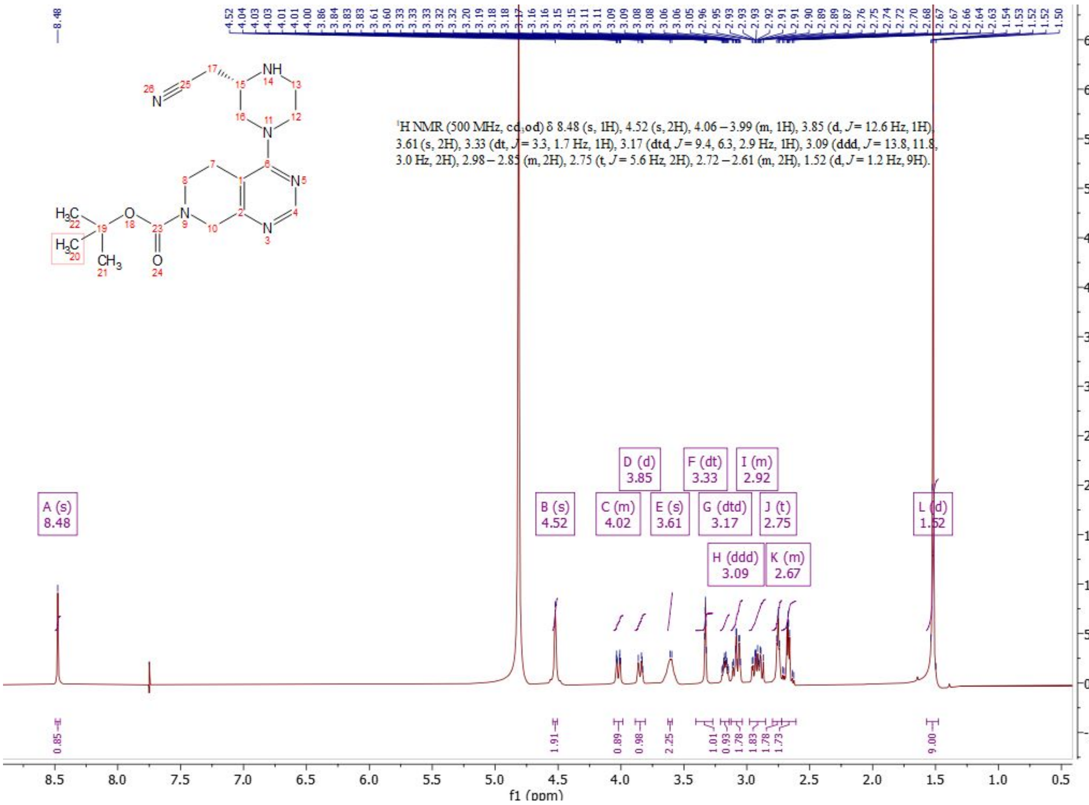
^{1}H NMR (500 MHz, cd3od) δ 8.48 (s, 1H), 4.52 (s, 2H), 4.06 – 3.99 (m, 1H), 3.85 (d, J = 12.6 Hz, 1H), 3.61 (s, 2H), 3.33 (dt, J = 3.3, 1.7 Hz, 1H), 3.17 (dtd, J = 9.4, 6.3, 2.9 Hz, 1H), 3.09 (ddd, J = 13.8, 11.8, 3.0 Hz, 2H), 2.98 – 2.85 (m, 2H), 2.75 (t, J = 5.6 Hz, 2H), 2.72 – 2.61 (m, 2H), 1.52 (d, J = 1.2 Hz, 9H).
A (s)
8.48
B (s)
4.52
C (m)
4.02
D (d)
3.85
E (s)
3.61
F (dt)
3.33
G (dtd)
3.17
H (ddd)
3.09
I (m)
2.92
J (t)
2.75
K (m)
2.67
L (d)
1.52
f1 (ppm)

14. References

(1) Strelow, J. M. A Perspective on the Kinetics of Covalent and Irreversible Inhibition. *SLAS Discovery* **2017**, *22* (1), 3–20. https://doi.org/10.1177/1087057116671509.

(2) Maurer, T. S.; Tabrizi-Fard, M. A.; Fung, H.-L. Impact of Mechanism-Based Enzyme Inactivation on Inhibitor Potency: Implications for Rational Drug Discovery. *J Pharm Sci* **2000**, *89* (11), 1404–1414. https://doi.org/10.1002/1520-6017(200011)89:11<1404::aid-jps4>3.0.co;2-#.

(3) Thorarensen, A.; Balbo, P.; Banker, M. E.; Czerwinski, R. M.; Kuhn, M.; Maurer, T. S.; Telliez, J.-B.; Vincent, F.; Wittwer, A. J. The Advantages of Describing Covalent Inhibitor in Vitro Potencies by IC50 at a Fixed Time Point. IC50 Determination of Covalent Inhibitors Provides Meaningful Data to Medicinal Chemistry for SAR Optimization. *Bioorg Med Chem* **2021**, *29*, 115865. https://doi.org/10.1016/j.bmc.2020.115865.

(4) Krippendorff, B.-F.; Neuhaus, R.; Lienau, P.; Reichel, A.; Huisinga, W. Mechanism-Based Inhibition: Deriving K I and k Inact Directly from Time-Dependent IC 50 Values. *J Biomol Screen* **2009**, *14* (8), 913–923. https://doi.org/10.1177/1087057109336751.

(5) Mader, L. K.; Keillor, J. W. Fitting of Kinact and KI Values from Endpoint Pre-Incubation IC50 Data. *ACS Med Chem Lett* **2024**, *15* (5), 731–738. https://doi.org/10.1021/acsmedchemlett.4c00054.

(6) Resnick, E.; Bradley, A.; Gan, J.; Douangamath, A.; Krojer, T.; Sethi, R.; Geurink, P. P.; Aimon, A.; Amitai, G.; Bellini, D.; Bennett, J.; Fairhead, M.; Fedorov, O.; Gabizon, R.; Gan, J.; Guo, J.; Plotnikov, A.; Reznik, N.; Ruda, G. F.; Díaz-Sáez, L.; Straub, V. M.; Szommer, T.; Velupillai, S.; Zaidman, D.; Zhang, Y.; Coker, A. R.; Dowson, C. G.; Barr, H. M.; Wang, C.; Huber, K. V. M.; Brennan, P. E.; Ovaa, H.; Von Delft, F.; London, N. Rapid Covalent-Probe Discovery by Electrophile-Fragment Screening. *J Am Chem Soc* **2019**, *141* (22), 8951–8968. https://doi.org/10.1021/jacs.9b02822.

(7) Keeley, A. B.; Kopranovic, A.; Di Lorenzo, V.; Ábrányi-Balogh, P.; Jänsch, N.; Lai, L. N.; Petri, L.; Orgován, Z.; Pölöske, D.; Orlova, A.; Németh, A. G.; Desczyk, C.; Imre, T.; Bajusz, D.; Moriggl, R.; Meyer-Almes, F. J.; Keserü, G. M. Electrophilic MiniFrags Revealed Unprecedented Binding Sites for Covalent HDAC8 Inhibitors. *J Med Chem* **2024**, *67* (1), 572–585. https://doi.org/10.1021/ACS.JMEDCHEM.3C01779.

(8) Ábrányi-Balogh, P.; Keeley, A.; Ferenczy, G. G.; Petri, L.; Imre, T.; Grabrijan, K.; Hrast, M.; Knez, D.; Ilaš, J.; Gobec, S.; Keserű, G. M. Next-Generation Heterocyclic Electrophiles as Small-Molecule Covalent MurA Inhibitors. *Pharmaceuticals* **2022**, *15* (12), 1484. https://doi.org/10.3390/PH15121484/S1.

(9) Kuljanin, M.; Mitchell, D. C.; Schweppe, D. K.; Gikandi, A. S.; Nusinow, D. P.; Bulloch, N. J.; Vinogradova, E. V.; Wilson, D. L.; Kool, E. T.; Mancias, J. D.; Cravatt, B. F.; Gygi, S. P. Reimagining High-Throughput Profiling of Reactive Cysteines for Cell-Based Screening of Large Electrophile Libraries. *Nature Biotechnology 2021 39:5* **2021**, *39* (5), 630–641. https://doi.org/10.1038/s41587-020-00778-3.

(10) Backus, K. M.; Correia, B. E.; Lum, K. M.; Forli, S.; Horning, B. D.; González-Páez, G. E.; Chatterjee, S.; Lanning, B. R.; Teijaro, J. R.; Olson, A. J.; Wolan, D. W.; Cravatt, B. F. Proteome-Wide Covalent Ligand Discovery in Native Biological Systems. *Nature* **2016**, *534*, 570. https://doi.org/10.1038/nature18002.

(11) Abbasov, M. E.; Kavanagh, M. E.; Ichu, T. A.; Lazear, M. R.; Tao, Y.; Crowley, V. M.; am Ende, C. W.; Hacker, S. M.; Ho, J.; Dix, M. M.; Suciu, R.; Hayward, M. M.; Kiessling, L. L.; Cravatt, B. F. A Proteome-Wide Atlas of Lysine-Reactive Chemistry. *Nature Chemistry 2021 13:11* **2021**, *13* (11), 1081–1092. https://doi.org/10.1038/s41557-021-00765-4.

(12) Thorarensen, A.; Dowty, M. E.; Banker, M. E.; Juba, B.; Jussif, J.; Lin, T.; Vincent, F.; Czerwinski, R. M.; Casimiro-Garcia, A.; Unwalla, R.; Trujillo, J. I.; Liang, S.; Balbo, P.; Che, Y.; Gilbert, A. M.; Brown, M. F.; Hayward, M.; Montgomery, J.; Leung, L.; Yang, X.; Soucy, S.; Hegen, M.; Coe, J.; Langille, J.; Vajdos, F.; Chrencik, J.; Telliez, J.-B. Design of a Janus Kinase 3 (JAK3) Specific Inhibitor 1-((2 S ,5 R)-5-((7 H -Pyrrolo[2,3- d]Pyrimidin-4-Yl)Amino)-2-Methylpiperidin-1-Yl)Prop-2-En-1-One (PF-06651600) Allowing for the Interrogation of JAK3 Signaling in Humans. *J Med Chem* **2017**, *60* (5), 1971–1993. https://doi.org/10.1021/acs.jmedchem.6b01694.

(13) Huynh, M. V.; Parsonage, D.; Forshaw, T. E.; Chirasani, V. R.; Hobbs, G. A.; Wu, H.; Lee, J.; Furdui, C. M.; Poole, L. B.; Campbell, S. L. Oncogenic KRAS G12C: Kinetic and Redox Characterization of Covalent Inhibition. *Journal of Biological Chemistry* **2022**, *298* (8), 102186. https://doi.org/10.1016/j.jbc.2022.102186.

(14) Li, K. S.; Quinn, J. G.; Saabye, M. J.; Guerrero, J. F. S.; Nonomiya, J.; Lian, Q.; Phung, W.; Izrayelit, Y.; Walters, B. T.; Gustafson, A.; Endres, N. F.; Beresini, M. H.; Mulvihill, M. M. High-Throughput Kinetic Characterization of Irreversible Covalent Inhibitors of KRASG12C by Intact Protein MS and Targeted MRM. *Anal Chem* **2022**, *94* (2), 1230–1239. https://doi.org/10.1021/ACS.ANALCHEM.1C04463.
